# SKA3-mediated hypoxia tolerance and metabolic reprogramming promote liver metastasis in lung adenocarcinoma

**DOI:** 10.1038/s41419-025-08270-z

**Published:** 2025-11-26

**Authors:** Zedong Sun, Minmin Zhou, Ningdan Song, Can Wang, Mingrui Liu, Simian He, Feifan Ji, Jiayu Chen, Lin Xu, Xiufen Zheng, Binhui Ren

**Affiliations:** 1 Department of Thoracic Surgery, Nanjing Medical University Affiliated Cancer Hospital & Jiangsu Cancer Hospital & Jiangsu Institute of Cancer Research, Nanjing, PR China; 2 Jiangsu Key Laboratory of Innovative Cancer Diagnosis & Therapeutics, Cancer Institute of Jiangsu Province, Nanjing, PR China; 3 The Fourth Clinical College of Nanjing Medical University, Nanjing, PR China; 4 China Pharmaceutical University, Nanjing, PR China; 5 Jiangsu Key Laboratory of Innovative Cancer Diagnosis & Therapeutics, Department of Thoracic Surgery, Nanjing Medical University Affiliated Cancer Hospital & Jiangsu Cancer Hospital & Jiangsu Institute of Cancer Research, Nanjing, PR China; 6 Key Laboratory of Emergency and Trauma of Ministry of Education, Department of Pharmacy and Engineering Research Center of Tropical Medicine Innovation and Transformation, The First Affiliated Hospital, Hainan Medical University, Haikou, PR China

**Keywords:** Cancer metabolism, Metastasis, Oncogenes, Ubiquitylation, Transcriptional regulatory elements

## Abstract

Late-stage lung adenocarcinoma (LUAD) frequently results in distant metastasis, with liver metastasis indicating the poorest prognosis. To successfully colonize the liver, metastatic LUAD cells must overcome its relatively hypoxic microenvironment. This study explores the metabolic adaptations that facilitate LUAD liver metastasis, identifying Spindle and Kinetochore Associated Protein 3 (SKA3) as a critical mediator. Under hypoxic conditions, SKA3 expression is significantly upregulated, driving glucose metabolic reprogramming in LUAD cells to enable survival within the liver’s hypoxic niche. Mechanistically, SKA3 competitively binds to prolyl hydroxylase domain-containing protein 2 (PHD2), disrupting its interaction with hypoxia-inducible factor 1-alpha (HIF-1α). Consequently, stabilized HIF-1α further enhances glycolytic enzyme transcription, amplifying glycolysis and enabling adaptation to liver hypoxia. Furthermore, hypoxia upregulates the E3 ubiquitin ligase MDM2, promoting p53 ubiquitination and degradation, thereby relieving p53-mediated repression of SKA3 and further reinforcing the SKA3/HIF-1α axis. Interestingly, HIF-1α directly binds to the hypoxia response element (HRE) in the SKA3 promoter, creating a positive feedback loop to maintain high SKA3 expression. Thus, SKA3-mediated metabolic reprogramming significantly contributes to LUAD cells colonization and proliferation in the liver. Finally, our findings demonstrated that the SKA3/HIF-1α axis was critical for establishing hypoxia tolerance in LUAD cells, underscoring its potential as a therapeutic target for treating liver metastasis in LUAD.

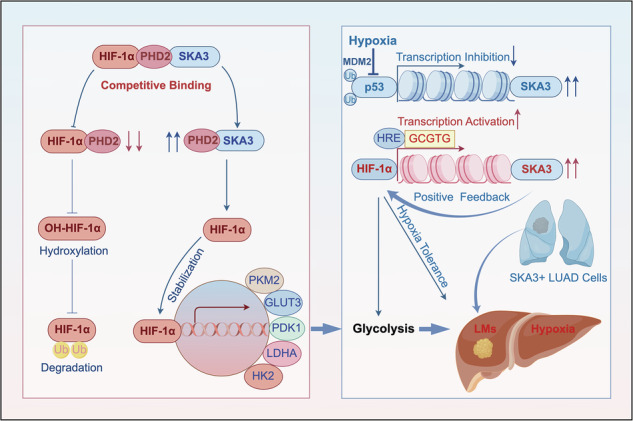

## Introduction

LUAD remains one of the leading causes of cancer-related mortality worldwide, with distant metastasis significantly reducing patient survival rates [1, 2]. Liver metastasis occurs in approximately 4% of non-small cell lung cancer (NSCLC) patients and is associated with a particularly poor prognosis, often resulting in median survival times of only 4 months [3, 4]. Epidemiological data from the Surveillance, Epidemiology, and End Results database further highlight the severe impact of liver metastasis on LUAD patient outcomes [5]. Simultaneously, a series of evidence indicates that the liver’s inherent immunosuppressive microenvironment complicates the clinical treatment of liver metastases [6]. Therefore, exploring the underlying mechanisms of liver metastasis in LUAD and identifying novel key biomolecules is of significant importance.

As a critical metabolic organ, the liver regulates the body’s energy balance [7]. To efficiently perform its various metabolic functions, the liver is divided into different metabolic zones, each corresponding to varying oxygen concentration gradients, creating an inhospitable environment for metastatic tumor cells [8]. Research has indicated that the liver microenvironment is more conducive to tumor cells with high glycolytic metabolism and those adapted to hypoxic conditions. For instance, primary hepatocellular carcinoma preferentially engages in glycolytic metabolism, facilitating its proliferation in the liver [9, 10]. In contrast, LUAD originates from a highly vascularized and oxygenated microenvironment. Thus, it is essential to investigate how LUAD cells overcome the liver’s relatively hypoxic barrier to establish colonization, as well as to determine whether hypoxia-tolerant LUAD cells are more prone to liver metastasis.

Tumor cells must undergo organ-specific metabolic adaptations to overcome the obstacles of metastasis to specific organs [11]. Metabolic reprogramming is considered a hallmark of tumor cells, primarily manifesting as the Warburg effect, where tumor cells rely on anaerobic glycolysis for energy production even under aerobic conditions [12]. This reprogramming is crucial for the metastatic process, as changes in metabolism affect not only the energy state of cells but also influence signaling networks and gene expression patterns through the modification of post-translational substrates and the utilization of cofactors [13, 14]. Additionally, metabolites secreted by cells can act as signaling molecules to alter the microenvironment and promote intercellular signaling [15–17]. Thus, it is logical that oncogenes promote tumor metastasis through metabolic alterations. For example, HIF-1α-mediated PDK1 expression plays a crucial role in facilitating liver metastasis [18]. To adapt to the varying oxygen requirements, energy sources, and nutritional demands of different environments, tumor cells require flexible metabolic adaptability. In solid tumors, when cells undergo internal infiltration and detach from their primary site to metastasize, the first step is metabolic reprogramming [11, 19]. HIF-1α plays a central role in tumor adaptation to hypoxic conditions by regulating key glycolytic enzymes, thus driving metabolic reprogramming essential for metastasis. Recent evidence suggests that HIF-1α-mediated pathways significantly contribute to LUAD liver metastasis. However, the precise regulatory mechanisms and critical molecular players involved in LUAD metabolic adaptation to liver hypoxia remain unclear.

Two pairs of clinical samples from patients with late-stage LUAD and their corresponding liver metastases were collected to establish a representative cohort. Furthermore, a liver metastasis model was established by injecting A549 cells into the spleen of nude mice, followed by screening for liver metastasis cells (A549-LMs). Considering that LUAD needs to overcome the hypoxic barrier during liver-specific metastasis, A549 cells were also cultured under hypoxic conditions in vitro. These models were then subjected to RNA sequencing (RNA-seq), which, through a combined screening approach, ultimately identified SKA3 as a key gene involved in liver metastasis of LUAD. As a subunit of the SKA complex, SKA3 is located at the outer kinetochore and interacts with the NDC80 complex. This interaction not only regulates and facilitates the exit from mitosis during mitotic division but also stabilizes the spindle during meiotic spindle migration and anaphase [20, 21]. Subsequent studies demonstrate that SKA3 promotes LUAD metastasis by binding to and activating the EGFR pathway, which induces the expression of matrix metalloproteinases-2, -7, and -9 [22]. Additionally, SKA3 plays a role in regulating tumor cell metabolism. For instance, SKA3 can enhance fatty acid metabolism, promoting malignant progression in pancreatic cancer [23]. Based on these studies, we propose that SKA3 is a pivotal gene regulating tumor cell metabolism and serves as a potential promoter of LUAD metastasis.

In this study, we found that SKA3 was highly expressed in LUAD liver metastasis cells; overexpression of SKA3 enhances glycolysis in LUAD cells, promoting their survival under hypoxic conditions and thereby driving liver metastasis of LUAD. Mechanistically, SKA3 interacted with PHD2, reducing the hydroxylation and ubiquitination of HIF-1α, thereby stabilizing HIF-1α protein levels and activating downstream glycolytic key enzymes to overcome the hypoxic barrier of the liver. Furthermore, we explored the reasons for the high expression of SKA3 in LUAD liver metastasis cells from the perspective of transcriptional regulation. Under relative hypoxia conditions of the liver, the transcriptional inhibition of SKA3 by p53 was reduced, while the transcriptional activation of SKA3 by HIF-1α was enhanced, forming a positive feedback loop for high SKA3 expression. Finally, we established hypoxia-tolerant LUAD cells, in which SKA3-mediated stabilization of HIF-1α enhanced the proliferation of hypoxia-tolerant cells. The combined inhibition of SKA3 and HIF-1α significantly impaired the survival of LUAD cells under hypoxic conditions. The combined inhibition of SKA3 and HIF-1α effectively suppresses LUAD liver metastasis, indicating that it may represent a promising therapeutic target.

## Materials and methods

### Clinical data and samples

Paired LUAD tissue microarrays were obtained from Nanjing You Meng Biotech Co., Ltd. (Cat. No. LucSur2202), and 80 pairs of paraffin-embedded LUAD sections were analyzed for SKA3 expression. All samples were confirmed by experienced pathologists. This study was approved by the Life Sciences Ethics Committee of Changsha Yaxiang Biotechnology Co., LTD. The Ethics report is available online at *yxswll.ccrl.cn*. The query code is QFWLZZJ04H8EMQ.

### Cell culture

A549 and PC9 cell lines were obtained from the Cell Bank of the Chinese Academy of Sciences (Shanghai, China) and were tested routinely for mycoplasma (last tested 2021.05). A549, A549-LMs, and PC9 cells were cultured in RPMI 1640 medium (KeyGene, Nanjing, China), both supplemented with 100 units/mL penicillin, 100 units/mL streptomycin, and 10% FBS (Thermo Fisher Scientific, MA, USA). All cells were cultured at 37 °C in an incubator containing 5% CO_2_ (Thermo Fisher Scientific, Waltham, MA, USA).

### Transfection and transduction of tumor cells

The siRNAs targeting SKA3 and HIF-1α were constructed and purchased from KeyGene, China. The full-length cDNA of human SKA3 and p53 plasmids was prepared by cloning into the expression vector pENTER (KeyGene, Nanjing, China). The siRNAs were transfected into LUAD cells using Imax (Invitrogen), and the plasmid vectors were transfected with Lipofectamine 3000 (Invitrogen, Carlsbad, CA, USA) according to the manufacturer’s instructions. For transduction of tumor cells, cells were plated at 5 × 10^4^ cells per mL in 24-well plates and transduced with lentiviral particles (multiplicity of infection of 10) with 5 μg/mL Polybrene. The oligonucleotide sequences of siRNAs and shRNAs are listed in Supplementary Table 1.

### Cell proliferation assays

For the 5-ethynyl-2′-deoxyuridine (EdU) assay (EdU Apollo®488 In Vitro Imaging Kit, RiboBio, Guangzhou, China), 10,000 cells/100 μL were plated in 96-well plates. Then the cells were incubated with 100 μL 50 μM EdU solution for 2 h and fixed with 50 μL 4% paraformaldehyde. And 50 μL 2 mg/mL glycine was added to neutralize paraformaldehyde, and the cells were washed with 100 μL 0.5% TritonX-100. After that, the cells were stained with 100 μL 1×Apollo solution and 100 μL 1×Hoechst33342 solution. After washing with 100 μL PBS three times, images were obtained from a fluorescence microscope for further calculation of proliferation rates.

For real-time cell analysis (RTCA), cells were seeded in C-plates, and then the plates were placed into the RTCA DP device and incubated at 37 °C with 5% CO_2_ to monitor the cell index value every 15 min for at least 40 h.

For the colony formation assay, 500 cells were seeded into 6-well plates in triplicate. Subsequently, the cells were stained with 0.1% crystal violet solution after 14 days. Cells were seeded into 96-well plates at a density of 2000 cells/100 μL in triplicate. Subsequently, they were Cell viability was assessed using the Cell Counting Kit-8 kit, and the absorbance at 450 nm was measured every 24 h.

### Transwell and migration assay

For transwell and matrigel assays, 5 × 10^4^ cells were seeded in the upper wells of 24-well chambers in medium without serum, and the lower wells were filled with medium containing 10% serum. After incubation for 24 h, the cells that migrated out onto the lower surface of the membranes were scored by staining with 1% crystal violet after fixing the cells in 4% paraformaldehyde.

### Glucose uptake, Lactate secretion, and Pyruvate production

Cells were cultured to ~40% confluency and then changed with fresh culture medium. After 24 h, the culture medium was collected, and measurement of glucose uptake, lactate secretion, and pyruvate production was performed using kits from Abcam (catalog nos. ab136955, ab65330, and ab65342) according to the manufacturer’s instructions. Cell counting was performed using a cell counter.

### Seahorse assay

An XF96 Extracellular Flux Analyzer (Agilent) was used to determine the extracellular acidification rates (ECAR). Briefly, 1 × 10^5^ cells per well with indicated treatments were seeded into 96-well Seahorse XF96 culture plates with 10% fetal bovine serum DMEM overnight. Cells were washed and incubated with base medium with 2 mM-glutamine for 1 h at 37 °C. Incubations were performed in a CO_2_-free incubator to ensure accurate measurements of extracellular pH. ECAR measurements were performed according to the manufacturer’s instructions. After every three measurements in 8-min intervals, glucose, oligomycin, or 2-DG was added into the wells at the indicated time points to a final concentration of 10 mM, 1 μM, or 50 mM, respectively.

### RNA extraction and qRT‒PCR

Total RNA was extracted from cultured cells or tissue samples with TRIzol reagent (Invitrogen) according to the manufacturer’s instructions. qRT‒PCR was performed using Fast SYBR® Green Master Mix (Thermo Fisher Scientific) on a QuantStudio 6 (Applied Biosystems, CA, USA) according to the manufacturer’s instructions. Primers are shown in Supplementary Table 2.

### Western blotting (WB)

Cultured cells were collected and lysed with lysis buffer (RIPA, Invitrogen) containing protease and phosphatase inhibitors. Protein extracts were resolved in a 10% SDS‒PAGE gel and transferred to a PVDF membrane. After blocking in 5% nonfat dry milk, the PVDF membranes were cut and incubated with diluted Primary antibodies shown in Supplementary Table 3 overnight. After washing in PBS-Tween 0.1% three times for 5 min, the PVDF membrane was incubated for 2 h with IRDye 800CW goat anti-mouse or IRDye 680CW goat anti-rabbit (Li-Cor Biosciences, NE, USA) secondary antibody. Finally, the signal was detected using an Odyssey scanner (Li-Cor Biosciences).

### Nuclear and cytoplasmic protein extraction

The nuclear and cytoplasmic fractions were isolated using the Cytoplasmic and Nuclear Protein Extraction kit (P0028, Beyotime). Western blotting was performed as described previously.

### Immunoprecipitation

Cells were lysed in IP lysis buffer and protease inhibitors. For immunoprecipitation, antibodies against PHD2 (1:200; 4835, CST), SKA3 (1:200; 186003, Abcam) were added to the lysates and incubated overnight at 4 °C, with rabbit IgG (1–5 μg) as the control antibody. Then Dynabeads Protein G (10007D, Invitrogen) was added and then incubated for 1 h at room temperature. An immunoblot was carried out using antibodies as indicated in Supplementary Table 3.

### Chromatin immunoprecipitation (ChIP)

Chromatin immunoprecipitation (ChIP) assays were performed using the Chromatin Immunoprecipitation Kit (26157, Invitrogen) according to the manufacturer’s protocol. A549 cells (1 × 10^7^) were collected and incubated with 4% paraformaldehyde for 10 min at room temperature for crosslinking, followed by incubation with 10× glycine for 5 min. Samples were sonicated, and DNA was sheared to an average length of ≈250–450 bp. DNA–protein complexes were immunoprecipitated using antibodies against p53 (5 μg; 1101, Abcam), HIF (5 μg; 08433, Abcam), or with polyclonal IgG control at 4 °C overnight. ChIP DNA samples were subjected to PCR amplification with primers specific to the SKA3 promoter region. The primer sequences are shown in Supplementary Table 2.

### Dual-luciferase reporter assays

SKA3 promoters were cloned into the pGL3 vector to generate pGL3-SKA3. Subsequently, HEK293T cells were transfected with 50 ng of pRL-TK Renilla luciferase expression vector and 1 μg of either pGL3 basic vector or pGL3-SKA3 plasmids. Luciferase activity was assayed 48 h after transfection using a Dual-Luciferase reporter assay system (Promega) according to the manufacturer’s instructions. Firefly luciferase activity was normalized to Renilla luciferase activity for each sample.

### CHX and MG132 assays

Cycloheximide (CHX; MedChemExpress, HY-12320) and MG132 (MedChemExpress, HY-13259) stock solutions were prepared in DMSO. For protein stability assays, A549 and A549-LM3 cells were incubated with CHX at a final concentration of 200 μg/mL for 0, 10, 20, 40, or 60 min, after which whole-cell lysates were harvested and subjected to Western blotting. To assess proteasome-dependent degradation, cells were treated with 25 μM MG132 for 24 h prior to lysis and immunoblot analysis.

### Immunofluorescence co-localization experiment

After sterilizing and cleaning the climbing slides, place them in a 24-well plate. Count 5 × 10^4^ cells and add them to the wells for culture in an incubator. When the cell density reaches approximately 60%–70%, discard the culture medium and fix the cells with 4% paraformaldehyde. Discard the fixative and wash the slides three times with pre-cooled PBS. Treat the cells with 100 μM digitonin at room temperature for 10 min. Block the slides with PBST containing 10% serum for 30 min. Incubate the slides overnight in a humid chamber at 4 °C with the primary anti-SKA3 (1:200; CSB-PA816899LA01HU, CUSABIO) antibody. Incubate with Alexa Fluor 488-conjugated anti-rabbit secondary antibody (dilution 1:250-1:1000) at room temperature in the dark for 1 h. Wash three times with PBS, each wash for 5 min. Incubate the slides overnight in a humid chamber at 4 °C with the primary anti-PHD2 (1:200; 66589-1-lg, Proteintech) antibody. Incubate with Alexa Fluor 488-conjugated anti-mouse secondary antibody (dilution 1:250-1:1000) at room temperature in the dark for 1 h. Wash three times with PBS, each wash for 5 min. Stain the slides with DAPI (dilution 1:1000, 1 mg/mL) at room temperature for 5 min. Capture images using a laser confocal microscope (OLYMPUS FV3000) at 600× magnification. Analyze co-localization using ImageJ software.

### Multiplex TSA immunofluorescence staining

Multiplex TSA immunofluorescence was performed on FFPE TMA sections to detect p53, HIF-1α, and SKA3. Slides were deparaffinized, rehydrated, and subjected to antigen retrieval in citrate buffer (pH 6.0) at 95 °C for 20 min. Endogenous peroxidases were quenched with 3% H₂O₂, and nonspecific binding was blocked in 5% normal goat serum for 30 min. Sections were incubated with anti-p53 overnight at 4 °C, followed by HRP-conjugated secondary antibody (30 min) and TSA amplification (Opal 650) for 10 min. After heat-mediated antibody stripping, the same steps were repeated for HIF-1α (Opal 520) and SKA3 (Opal 570). Nuclei were counterstained with DAPI, and slides were mounted in anti-fade medium. Images were acquired on a multispectral fluorescence microscope under identical exposure settings.

### Spleen injection liver metastasis model

A total of 30 six-week-old female BALB/c nude mice, weighing 18–22 g, were selected and divided into groups according to the experimental purpose: a PX-478 (HIF-1α inhibitor; HY-1023, MedChemExpress) treatment group and a DMSO treatment group as a control. Within each group, mice were randomly assigned to subgroups with one million A549 or PC9 cells in 100 μL PBS with SKA3 overexpressing or knocking down. These cells were injected into the spleen of the mice. Seven days post-injection, the mice received PX-478 treatment administered orally at a dose of 50 mg/kg/day for two consecutive weeks. Four weeks later, in vivo imaging was conducted to observe the metastatic lesions. The licensing committee of Nanjing Medical University authorized the animal studies. Nanjing Jichu Biomedical Technology Company (Nanjing, China) provided the mice.

### Metabolic profiling analysis

A549 (Ctrl-NC), A549-LMs, and A549 (Sh-SKA3) cells were cultured in 10-cm dishes with six replicates per group. Upon reaching 80%–90% confluence, the cells were promptly washed three times with ice-cold PBS, harvested with 1 mL of methanol/water solution (4:1, v/v), and treated by vortexing. The nontarget metabolomics analysis was performed by Biotree Biomedical Technology Co. (Shanghai, China).

### Statistical analysis

Statistical analyses were performed using GraphPad Prism 9.0 and SPSS 16.0 software. Quantitative data are expressed as mean ± standard deviation from a minimum of three independent experiments. Differences between the two groups were analyzed using Student’s *t*-test. Statistical analyses are detailed in the figure legends. A *p* value of <0.05 was considered statistically significant (ns: not significant, *p < 0.05, **p < 0.01, ***p < 0.001).

## Results

### SKA3 enabled LUAD cells to overcome hepatic hypoxic barriers and facilitated liver Metastasis

**Fig. 1:**
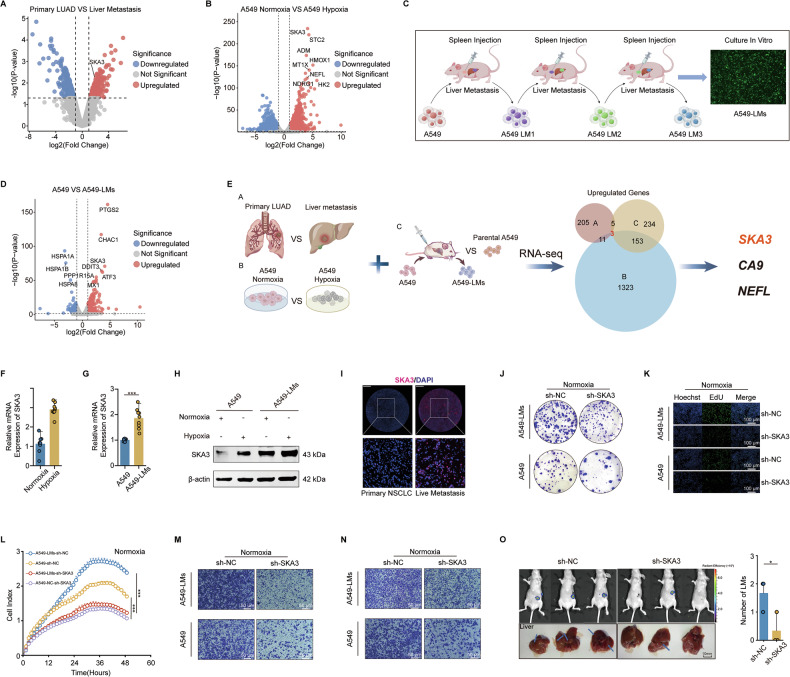
SKA3 overcame the hepatic hypoxia barrier to promote liver metastasis in LUAD. **A** Volcano plot of differentially expressed genes between LUAD and liver metastasis samples revealed elevated expression of SKA3 in liver metastasis. **B** Volcano plot of differentially expressed genes that were identified through transcriptome sequencing of A549 cells under normal and hypoxic conditions. **C** Schematic diagram illustrating the establishment of a LUAD liver metastasis model by splenic injection in mice, followed by the isolation of liver-metastatic A549 cells (A549-LMs). **D** Volcano plot of differentially expressed genes identified by transcriptome sequencing between parental A549 cells and A549-LMs cells. **E** Flowchart for identifying key genes associated with liver metastasis in LUAD. **F**–**H** qRT-PCR and WB analysis indicated that the expression of SKA3 in the A549-LMs cell line was higher compared to A549, and SKA3 expression increased under hypoxic conditions. **I** Immunofluorescence staining of SKA3 (red) and DAPI (blue) in primary NSCLC and live metastatic tissues, with images magnified 100 times; Scale bar: 2 mm. **J** Colony formation assay showed colony numbers of the sh-SKA3 group were less than control group. **K** EdU assay showed the positive signal of the sh-SKA3 group was weaker than the control group; Scale bar: 100 μm. **L** Real-time cellular analysis (RTCA) assay showed knockdown of SKA3 inhibited the proliferation of A549 and A549-LMs cells. Transwell (**M**) and Matrigel (**N**) assays revealed that knockdown of SKA3 inhibited the migration and invasion of A549 and A549-LMs cells; Scale bar: 50 μm. **O** The mouse spleen injection model demonstrated that the knockdown of SKA3 effectively reduced the number of liver metastases (*n* = 3). *p < 0.05; **p < 0.01; ***p < 0.001; ns no significance.

To identify critical factors involved in the liver metastasis of LUAD, we collected two pairs of primary LUAD tissues and matched liver metastasis samples (Fig. 1A). Previous studies have demonstrated that cancer cells metastasizing to the liver must adapt to the organ’s hypoxic microenvironment to successfully colonize and proliferate [11]. Given that LUAD cells must similarly overcome hypoxic stress during liver metastasis, we cultured A549 LUAD cells under normoxic and hypoxic conditions in vitro to simulate this environment (Fig. 1B). Additionally, we established a liver metastasis model in vivo by injecting A549 cells stably expressing pEGFP-N1 into the spleens of nude mice. After 21 days, liver-metastatic tissues were harvested, dissociated into single-cell suspensions, and GFP-positive cells were sorted. This procedure yielded a liver-metastatic LUAD cell line, designated A549-LM1. These cells were repeatedly sorted to establish a stable, liver-metastatic subline (A549-LMs) suitable for further experiments (Fig. 1C, D). Next, we performed RNA-seq to screen for genes significantly associated with liver metastasis (Supplementary Fig. 1A). Based on the sequencing results, we selected three potential genes, SKA3, CA9 (Carbonic Anhydrase IX), and NEFL (Neurofilament Light Chain), that may promote liver metastasis in LUAD (Fig. 1E). Compared with parental A549 cells, SKA3 was the only gene among the screened candidates that showed high expression A549-LMs cells, while CA9 and NEFL did not exhibit such an elevation. Furthermore, we confirmed that hypoxic conditions elevated both SKA3 mRNA and protein expression (Fig. 1F–H; Supplementary Fig. 1B–D). Immunofluorescence staining of 5 cases of LUAD and their liver metastasis samples revealed that SKA3 was highly expressed in the liver-metastatic foci of LUAD (Fig. 1I; Supplementary Fig. 1E). Additionally, analysis of public databases showed no expression difference of SKA3 in brain and bone metastases of NSCLC (Supplementary Fig. 1F, G). Taken together, SKA3 was a potential candidate gene that promotes liver-specific metastasis in LUAD.

To elucidate the functional role of SKA3, we conducted a series of in vitro assays. Colony formation, EdU incorporation, RTCA, transwell migration, and matrigel invasion assays consistently showed that SKA3 knockdown significantly impaired the proliferation, migration, and invasion capabilities of both A549 and A549-LMs cells (Fig. 1I–N; Supplementary Fig. 1H). Similar inhibitory effects were observed under hypoxic culture conditions (Supplementary Fig. 1I–N), indicating that SKA3 contributes significantly to LUAD cell aggressiveness in hypoxic microenvironments. To further confirm the role of SKA3 in promoting liver metastasis, we performed spleen injections of A549 cells transduced with a non-targeting control (sh-NC) or an SKA3-specific shRNA (sh-SKA3) into mice. Notably, liver metastases were observed, with significantly fewer metastatic lesions in the SKA3 knockdown group compared to the control group (Fig. 1O).

We further assessed the clinical significance of SKA3 in LUAD, mIHC staining of 80 pairs of LUAD and adjacent normal tissues indicated that SKA3 expression levels were significantly higher in LUAD tissues (Supplementary Fig. 1O, P). We further analyzed the TCGA database and found that SKA3 expression was higher in LUAD tissues compared to normal tissues (Supplementary Fig. 1Q). Moreover, SKA3 expression was higher in stages III–IV LUAD than in stages I–II (Supplementary Fig. 1R). Additionally, Kaplan-Meier Plotter online survival analysis revealed that LUAD patients with high SKA3 expression had significantly shorter overall survival (Supplementary Fig. 1S, log-rank test, *P* < 0.001). Collectively, these data indicated that SKA3 was a critical mediator of LUAD liver metastasis by facilitating cellular adaptation to hypoxic hepatic microenvironments, ultimately enhancing metastatic colonization and proliferation.

### SKA3 enhances glycolytic metabolism to overcome hypoxic barriers and facilitate liver metastasis in LUAD

**Fig. 2:**
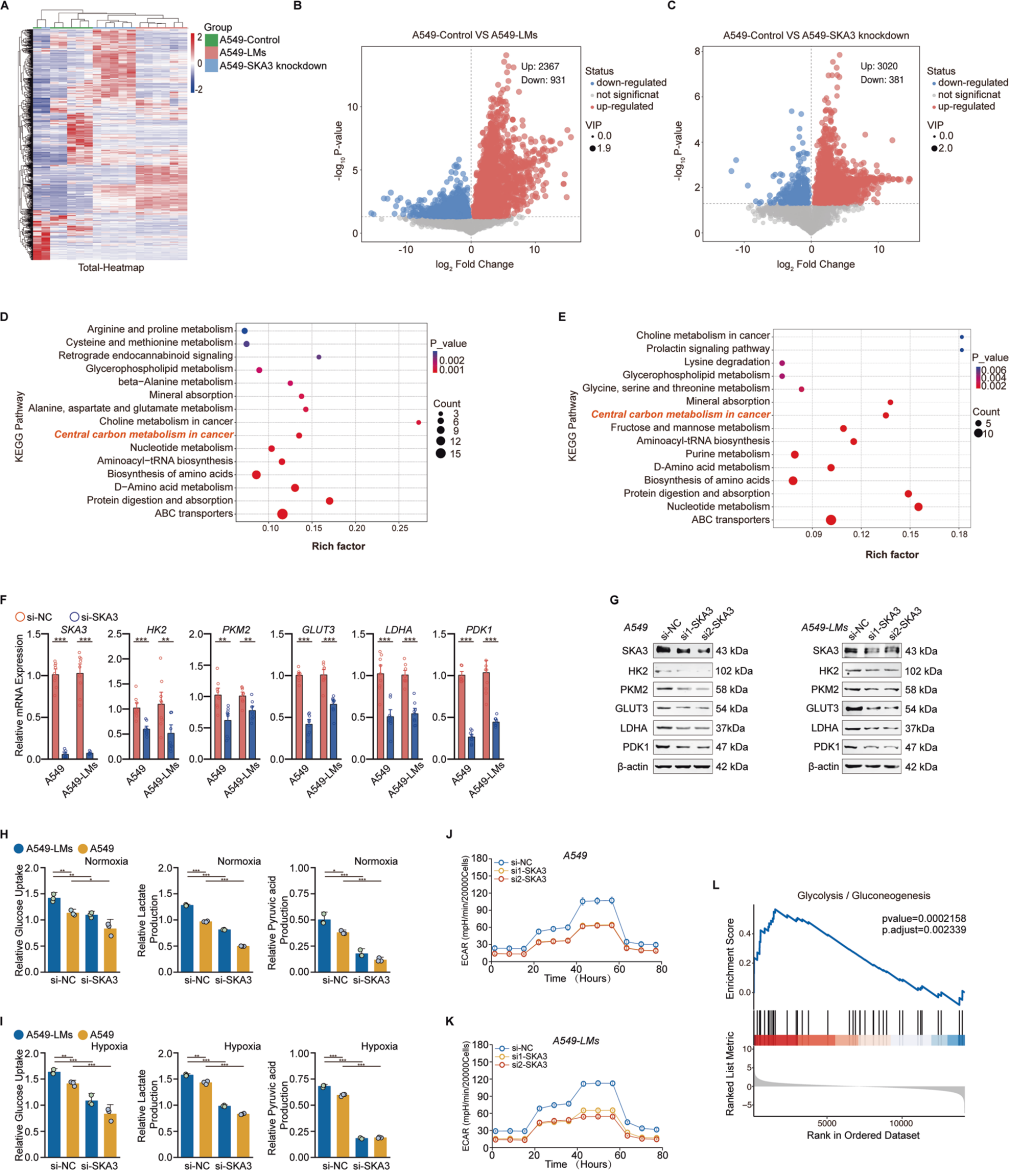
SKA3-mediated enhancement of glycolysis facilitated overcoming hypoxic barriers and promoted liver metastasis in LUAD. **A** Heatmap of hierarchical clustering analysis across all sample groups—including A549-Control, A549-LMs, and A549-SKA3 knockdown cells (*n* = 6)—showing transcriptome-wide expression patterns. A549-SKA3 knockdown cells were generated by stable lentiviral transduction of shRNA targeting SKA3. **B** Volcano plot of differential metabolites between A549 and A549-LM cells; **C** volcano plot of differential metabolites between A549-Control and A549-SKA3 knockdown cells. **D** Pathway analysis of differential metabolites between the A549-Control and A549-LMs groups showed a high correlation with the “central carbon metabolism in cancer” pathways. **E** KEGG pathway analysis of differential metabolites between the A549-Control and A549-SKA3 knockdown groups indicated that SKA3 may be involved in “central carbon metabolism in cancer.” **F**, **G** qRT-PCR and WB analysis indicated the expression levels of key glycolytic enzymes, including HK2, PKM2, GLUT3, LDHA, and PDK1, were significantly reduced following SKA3 knockdown in A549 and A549-LM cells. **H**, **I** After SKA3 knockdown, glucose uptake, lactate secretion, and pyruvate production were reduced in A549 and A549-LM cells under both normoxia and hypoxia conditions, as detected by ELISA kits. **J**, **K** Seahorse assay indicated that knockdown of SKA3 inhibited glycolytic capacity in A549 and A549-LMs cells. **L** GSEA of RNA-seq data comparing A549-LMs to A549-Control cells revealed significant positive enrichment of the glycolysis gene set in A549-LMs. *p* < 0.05; **p < 0.01; ***p < 0.001; ns no significance.

Cancer cells must undergo organ-specific metabolic adaptation to overcome the barriers of metastasis to specific organs, with the initial step being metabolic reprogramming [24]. Therefore, from a metabolic perspective, it is crucial to explore the adaptive metabolic changes that enable LUAD cells to withstand the liver’s relatively hypoxic environment and to elucidate the biological role of SKA3 in this process. Based on this premise, we selected human cell samples, grouped into A549-Control, A549-LMs, and A549-SKA3 knockdown cells, with six biological replicates per group, totaling 18 samples for metabolomic analysis (Fig. 2A). Metabolite data were normalized, and clustering heatmap analysis was performed. Based on the Variable Importance in the Projection > 1 and *P* value < 0.05 from the first principal component of the OPLS-DA model, differential metabolites were selected and visualized using volcano plots for the A549-Control vs. A549-LMs and A549-Control vs. A549-SKA3 knockdown groups (Fig. 2B, C). Subsequent metabolic pathway analysis indicated that A549-LMs cells are more actively involved in central carbon metabolism in cancer, including glycolysis, the tricarboxylic acid cycle, and oxidative phosphorylation (Fig. 2D). Further KEGG pathway analysis of differential metabolites in the A549-Control vs. A549-SKA3 knockdown groups revealed similar results (Fig. 2E). These results suggested that SKA3 may promote liver metastasis by modulating glucose metabolism in LUAD cells.

To assess the impact of SKA3 on glycolysis in LUAD cells, we first evaluated the expression levels of key glycolytic enzymes, including hexokinase 2 (HK2), pyruvate kinase M2 (PKM2), glucose transporter 3 (GLUT3), lactate dehydrogenase A (LDHA), and pyruvate dehydrogenase kinase 1 (PDK1), using qRT-PCR and Western blotting. Results indicated that SKA3 knockdown significantly reduced the mRNA and protein levels of these enzymes in both A549 and A549-LMs cells (Fig. 2F, G). Additionally, we employed ELISA kits to measure the impact of SKA3 on the glycolytic profile of LUAD cells under normoxic or hypoxic conditions. SKA3 knockdown led to significant reductions in glucose uptake, lactate secretion, and pyruvate production in A549 and A549-LMs cells (Fig. 2H, I). Further, Seahorse extracellular flux analysis confirmed these results, demonstrating that SKA3 knockdown significantly decreased ECAR in LUAD cells (Fig. 2J, K). Finally, Gene Set Enrichment Analysis (GSEA) further confirmed a significant positive correlation between glycolytic metabolism and liver metastasis propensity in A549-LMs cells (Fig. 2L). Taken together, these results indicated that SKA3 promoted LUAD liver metastasis through metabolic reprogramming that enhanced glycolysis, enabling cancer cells to overcome hypoxic hepatic barriers and colonize the liver more efficiently.

### SKA3 enhances glycolysis in LUAD cells by stabilizing HIF-1α via interaction with PHD2

**Fig. 3:**
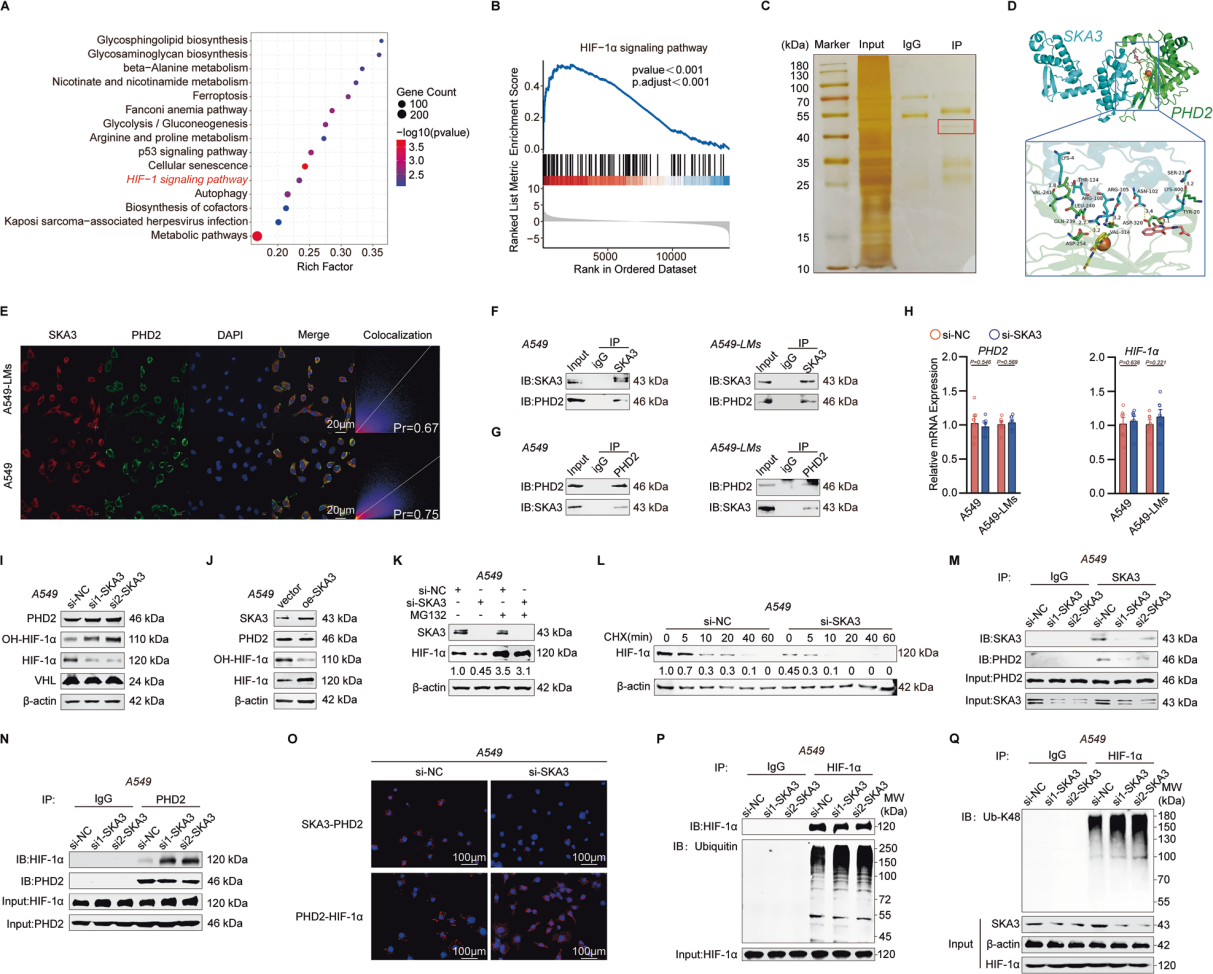
SKA3 enhanced the glycolysis of LUAD cells by stabilizing the HIF-1α protein. **A** Pathway enrichment analysis indicated that the HIF-1α pathway was enriched in A549-LMs. **B** GSEA analyses suggested that the HIF-1α pathway may be activated in A549-LMs. **C** Immunoprecipitation (IP) was performed with SKA3 antibody, and total lysate from A549 cells (input) was used as a positive control, while IgG antibody was used as a negative control. Representative images of silver-stained PAGE gel showed separated proteins. **D** The protein interaction between SKA3 and PHD2 was analyzed using HDOCK software. **E** Immunofluorescence co-localization analysis showed SKA3 (Red) co-localized with PHD2 (Green) in A549 and A549-LMs cells, assessed by Pearson’s coefficient (Pr), nuclei were counterstained with DAPI (blue). Scale bar: 20 μm. **F**, **G** Co-IP experiments verified the interaction between SKA3 and PHD2 in A549 and A549-LMs cells. **H** qRT-PCR results revealed no impact of SKA3 knockdown on PHD2 and HIF-1α mRNA expression in A549 and A549-LMs cells. **I** WB analysis indicated increased OH-HIF-1α and decreased HIF-1α levels, with no significant change in PHD2 and VHL protein levels following SKA3 knockdown in A549 cells. **J** WB analysis indicated decreased OH-HIF-1α and increased HIF-1α levels after SKA3 overexpression, without significant changes in PHD2 and VHL protein levels in A549 cells. **K** Knockdown of SKA3 did not affect HIF-1α protein levels in A549 cells treated with the proteasome inhibitor MG132 (25 μM), the numbers represented relative HIF-1α expression. **L** Knockdown of SKA3 accelerated degradation of HIF-1α protein in A549 cells treated with the transcription inhibitor CHX (200 μg/mL), the numbers represented relative HIF-1α expression. **M** IP assays demonstrated that the interaction of SKA3 and PHD2 was weakened in A549 cells after being transfected with SKA3 siRNA. **N** IP assays demonstrated that the interaction of HIF-1α and PHD2 was strengthened in A549 cells after transfected with SKA3 siRNA. MG132 (25 μM) was added to inhibit HIF-1α degradation. **O** Proximity ligation assay (PLA) showing protein interactions in A549 cells. The upper panel displays the interaction between SKA3 and PHD2 (red puncta), while the lower panel shows the interaction between PHD2 and HIF-1α (red puncta) in both control (si-Ctrl) and SKA3 knockdown (si-SKA3) cells. Nuclei are stained with DAPI (blue); Scale bars: 100 µm. **P** IP assays revealed that HIF-1α ubiquitination levels were increased in A549 cells after SKA3 siRNA transfection. MG132 (25 μM) was added to inhibit HIF-1α degradation. **Q** IP assays followed by immunoblotting (IB) with an antibody specific for K48-linked ubiquitin chains (Ub-K48), SKA3 knockdown increased Ub-K48 of HIF-1α in A549 cells. *p < 0.05; **p < 0.01; ***p < 0.001; ns no significance.

Numerous molecules influence glycolysis by regulating key metabolic enzymes. Pathway enrichment and GSEA analyses suggested that the HIF-1α signaling pathway might be activated in liver-metastatic LUAD cells, potentially due to the role of HIF-1α in promoting glycolytic metabolism (Fig. 3A, B). Given our earlier findings indicating that SKA3 contributed to glucose metabolic reprogramming in LUAD cells, we hypothesized that SKA3 might enhance glycolysis through regulation of the HIF-1α pathway. To explore whether SKA3 promotes LUAD progression through protein-protein interactions, we performed immunoprecipitation (IP) assays for SKA3, followed by silver staining and mass spectrometry analysis, to identify candidate interacting proteins (Fig. 3C). Among the identified proteins, Prolyl Hydroxylase Domain-containing protein 2 (PHD2) emerged as a potential binding partner. PHD2 is known to mediate HIF-1α hydroxylation (OH-HIF-1α), facilitating its subsequent recognition by von Hippel-Lindau (VHL) protein and leading to ubiquitination-dependent proteasomal degradation [25, 26]. The PHD2/HIF-1α-mediated oxygen-dependent signaling pathway plays a crucial role in glycolysis regulation [27]. Protein structure analysis revealed stable hydrogen bonding interactions between SKA3 and PHD2 (Fig. 3D; Supplementary Fig. 2A). We subsequently validated the interaction between SKA3 and PHD2 using co-immunoprecipitation (Co-IP) assays and confirmed their co-localization in A549 and A549-LMs cells via immunofluorescence (Fig. 3E–G). Notably, under both normoxic and hypoxic conditions, no direct interaction between SKA3 and HIF-1α was observed (Supplementary Fig. 2B, C). These findings demonstrated a direct protein-protein interaction specifically between SKA3 and PHD2.

To further elucidate the functional relevance of this interaction, we examined the effects of SKA3 knockdown on HIF-1α protein stability. SKA3 knockdown increased OH-HIF-1α levels while decreasing total HIF-1α protein levels, without affecting the mRNA expression of either PHD2 or HIF-1α, nor altering PHD2 and VHL protein expression levels (Fig. 3H, I; Supplementary Fig. 2D). Conversely, SKA3 overexpression resulted in reduced OH-HIF-1α and increased total HIF-1α protein expression (Fig. 3J; Supplementary Fig. 2E). Treatment with the proteasome inhibitor MG132 revealed that HIF-1α protein levels remained unchanged following SKA3 knockdown, indicating that SKA3 did not regulate the synthesis of HIF-1α (Fig. 3K; Supplementary Fig. 2F). Moreover, treatment with cycloheximide (CHX), a protein synthesis inhibitor, significantly reduced the half-life of HIF-1α in SKA3-knockdown A549 and A549-LMs cells compared to control cells, indicating enhanced protein degradation (Fig. 3L, Supplementary Fig. 2G). Experiments involving CHX and MG132 demonstrated that HIF-1α protein degradation accelerated after SKA3 knockdown. Given these results, we hypothesized that SKA3 interaction with PHD2 might regulate HIF-1α hydroxylation status. IP assays further demonstrated that SKA3 knockdown reduced SKA3-PHD2 binding affinity (Fig. 3M; Supplementary Fig. 2H), and concurrently increased PHD2-HIF-1α binding interactions (Fig. 3N; Supplementary Fig. 2I). Proximity ligation assays (PLAs) corroborated these findings (Fig. 3O; Supplementary Fig. 2J). Additionally, ubiquitination levels of HIF-1α increased following SKA3 knockdown (Fig. 3P**;** Supplementary Fig. 2K). Since K48-linked ubiquitination targets HIF-1α for proteasomal degradation, we next evaluated the extent of its K48-linked ubiquitination. Compared with si-NC, both SKA3 siRNAs markedly increased the smear of K48-linked ubiquitinated HIF-1α in A549 and A549-LMs cells (Fig. 3Q; Supplementary Fig. 3L). These findings suggested that SKA3 competitively bound to PHD2, disrupting the PHD2-mediated hydroxylation of HIF-1α and potentially affecting its subsequent ubiquitination and degradation.

HIF-1α enters the nucleus and forms a dimer with the HIF-1β subunit, which binds to hypoxia response elements (HREs) in target genes to activate transcription [28]. Previous studies have identified HK2, PKM2, GLUT3, LDHA, and PDK1 as HIF-1 target genes [29]. Therefore, to validate whether SKA3 influences nuclear accumulation of HIF-1α, we conducted nuclear-cytoplasmic fractionation and immunofluorescence assays. These assays revealed decreased nuclear localization of HIF-1α following SKA3 knockdown (Supplementary Fig. 3A–C). In conclusion, our data suggested that SKA3 stabilized HIF-1α protein by competitively inhibiting PHD2-mediated hydroxylation and subsequent degradation, thereby enhancing nuclear HIF-1α accumulation and transcriptional activation of glycolytic genes. This molecular mechanism elucidated how SKA3 promoted glycolytic metabolism in LUAD cells, facilitating adaptation to hypoxia and liver metastasis progression.

### SKA3-mediated HIF-1α stabilization promoted liver metastasis in LUAD

**Fig. 4:**
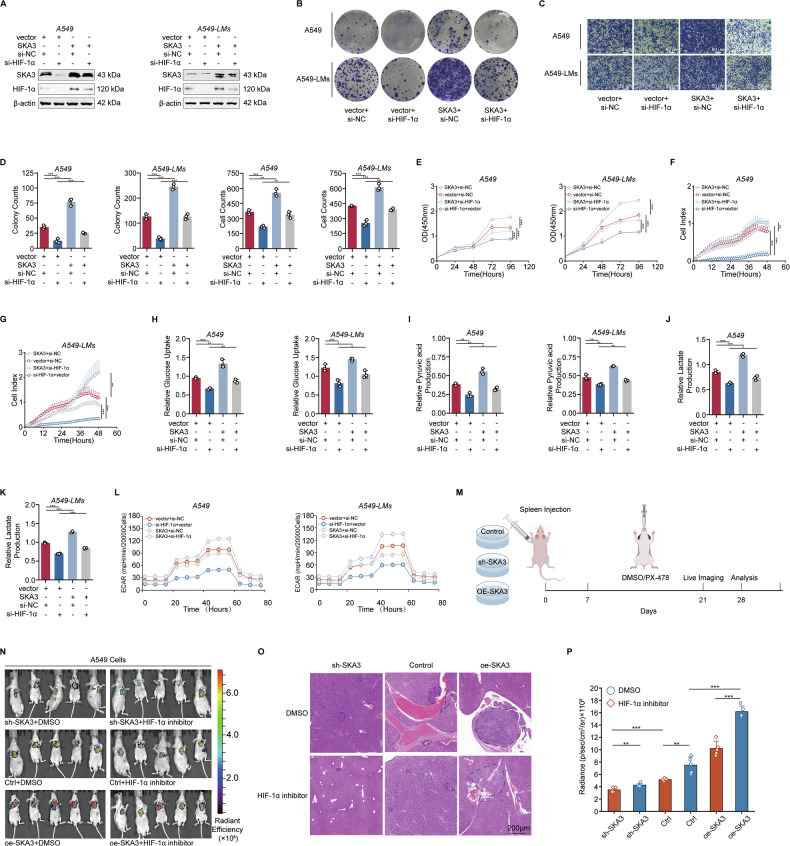
SKA3-mediated HIF-1α stabilization promoted liver metastasis in LUAD. **A** WB analysis demonstrated that SKA3 overexpression could inhibit the effects caused by treatment of HIF-1α siRNA in A549 and A549-LMs cells. **B**–**G** Colony formation (**B**), transwell (**C**), CCK-8 (**E**), and RTCA (**F**) assays revealed that SKA3 overexpression rescued the inhibition of cell proliferation or migration ability caused by HIF-1α silencing in A549 and A549-LMs cells, and corresponding quantitative results were shown (**D**). Overexpression of SKA3 could reverse the decrease in glucose uptake (**H**), pyruvate production (**I**), lactate secretion (**J**, **K**) levels, and glycolytic capacity (**L**) in A549 and A549-LMs cells caused by HIF-1α knockdown. **M** Schematic diagram of an in vivo liver-metastatic model. **N**–**P** LUAD liver metastasis models were constructed using SKA3 knockdown, overexpression, or control A549 cells. The mice were administered the HIF-1α inhibitor (PX-478) by gavage, while the control group received DMSO. Bioluminescence imaging was performed after 4 weeks (**N**), HE staining (**O**). The dashed lines indicate the boundaries of H&E-stained liver-metastatic lesions and the corresponding fluorescence quantification. **P** Results were shown. *p < 0.05; **p < 0.01; ***p < 0.001; ns no significance.

SKA3 promoted liver metastasis of LUAD by enhancing glycolysis to overcome the hepatic hypoxic barrier. Therefore, it was valuable to further investigate whether SKA3 exerted these effects by acting on HIF-1α. We conducted rescue experiments by overexpressing SKA3 on the basis of HIF-1α knockdown in LUAD cells. WB experiments showed that the overexpression of SKA3 could reverse the reduction in HIF-1α protein expression caused by HIF-1α knockdown (Fig. 4A). Additionally, colony formation, transwell, CCK-8, and RTCA assays demonstrated that HIF-1α knockdown reduced the proliferation and migration abilities of LUAD cells, and overexpression of SKA3 could reverse these effects (Fig. 4B–G). Further analysis of glucose metabolism in tumor cells showed similar results. Inhibition of HIF-1α significantly reduced glucose uptake, pyruvate production, lactate secretion, and glycolysis levels in LUAD cells, while overexpression of SKA3 reversed the reduction in glycolysis levels caused by HIF-1α knockdown (Fig. 4H–L).

To further verify in vivo that SKA3 promoted liver metastasis of LUAD through HIF-1α-mediated reprogramming of glucose metabolism, we injected stable SKA3 knockdown and overexpression A549 cells into the spleens of nude mice to establish a liver metastasis model of LUAD, with control groups set up simultaneously. On this basis, we administered the HIF-1α inhibitor (PX-478) for in vivo rescue experiments [30]. After 4 weeks, tumor growth was observed via bioluminescence imaging, and tumor size was quantified by fluorescence (Fig. 4M, Supplementary Fig. 4A, B). The results showed that SKA3 promotes liver metastasis of LUAD, while the HIF-1α inhibitor PX-478 significantly inhibited liver metastasis. Overexpression of SKA3 reversed the reduction in liver metastasis caused by HIF-1α inhibition. Notably, the combined inhibition of SKA3 and HIF-1α resulted in the least liver metastasis (Fig. 4N–P). Similar results were also observed in PC9 cells (Supplementary Fig. 4C–E). Proteins were extracted from mouse liver metastases to assess in vivo the impact of SKA3 on HIF-1α expression and its downstream regulation of key glycolytic enzymes. Compared with the vector control group, SKA3-overexpressing metastases exhibited markedly elevated protein levels of HIF-1α and its downstream glycolytic enzymes (Supplementary Fig. 4F). Based on these findings, SKA3-mediated HIF-1α stabilization enhanced glycolysis, enabling tumor cells to tolerate hypoxia and thereby promoting liver metastasis in LUAD.

### Hypoxia-induced p53 dysfunction strengthened the SKA3–HIF-1α axis

**Fig. 5:**
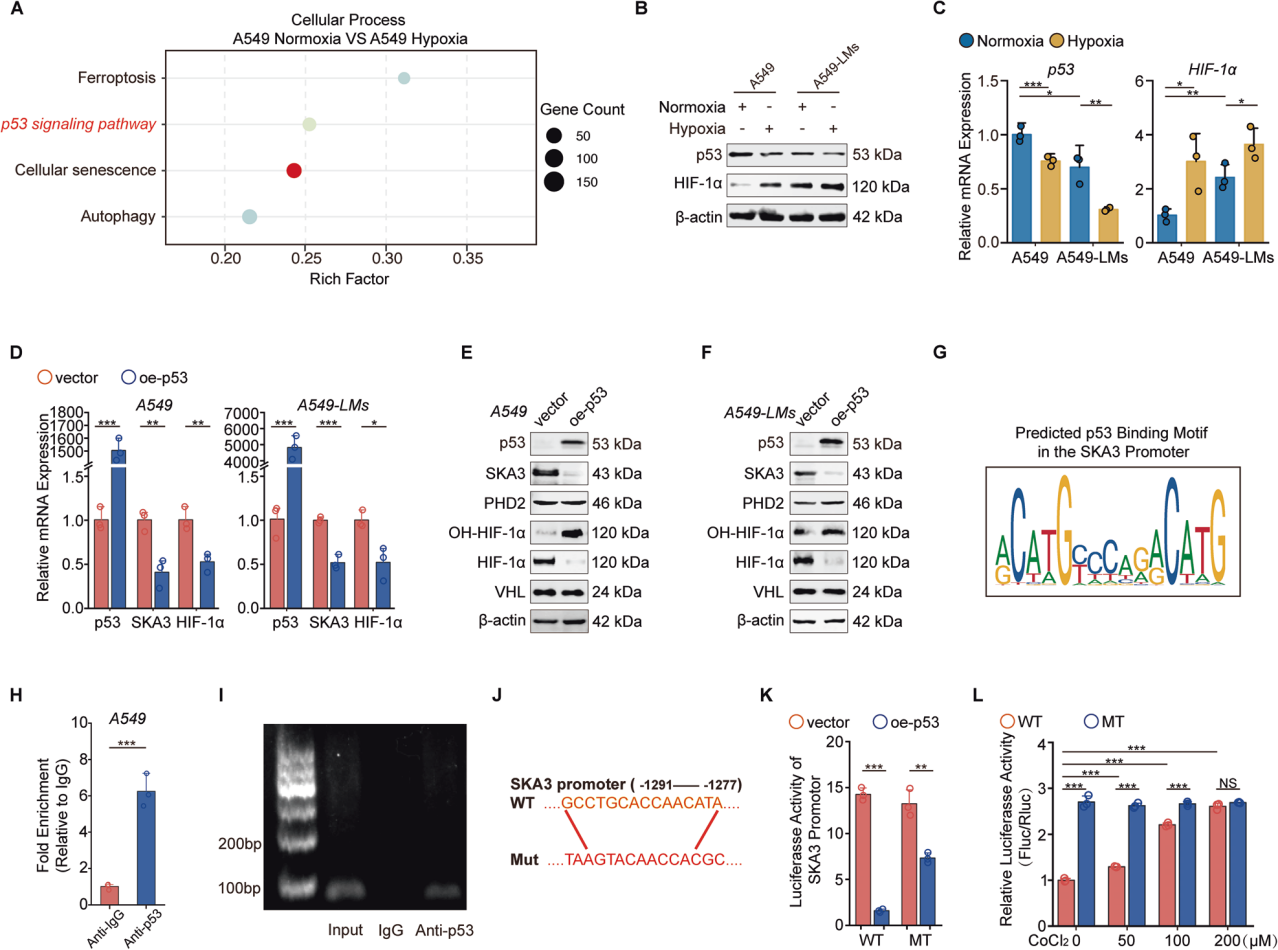
Hypoxia-induced p53 dysfunction strengthened the SKA3–HIF-1α axis. **A** Cellular process analysis indicated dysregulation of the p53 signaling pathway under hypoxic conditions. **B**, **C** WB and qRT-PCR analysis showed hypoxia suppressed p53 expression levels and promoted HIF-1α expression. **D**–**F** qRT-PCR and WB experiments showed the SKA3/HIF-1α pathway was downregulated after transfected with the p53 overexpressed plasmid; empty vector pcDNA3.1 was transfected as a control. **G** JASPAR website (https://jaspar.genereg.net/) predicted binding sites of p53 in the SKA3 promoter region; **H** ChIP-qPCR experiments validated the binding of p53 to the SKA3 promoter region, IgG was used as a negative control. **I** DNA gel electrophoresis confirmed the PCR products as SKA3. **J** SKA3 wild-type (WT: GCCTGCACCAACATA)/mutant-type (MT: TAAGTACAACCACGC) promoter reporter plasmids were constructed based on predicted binding regions. **K** Dual-luciferase reporter plasmid experiments confirmed p53 transcriptional inhibition of SKA3. **L** Dual-luciferase reporter gene experiments showed that with increasing hypoxia severity, p53’s transcriptional inhibitory function on SKA3 was attenuated. A549 cells with CoCl_2_ treatment (0, 50, 100, 200, 250 µM) were transfected with SKA3 promoter-WT or Mut reporters for 24 h. *p < 0.05; **p < 0.01; ***p < 0.001; ns no significance.

To further elucidate the mechanisms underlying elevated SKA3 expression under hypoxic conditions and during liver metastasis, we investigated factors inducing SKA3 upregulation and the regulatory pathways controlling its expression in hypoxia-tolerant LUAD cells. Cellular process analysis indicated dysregulation of the p53 signaling pathway under hypoxia (Fig. 5A). As a crucial tumor suppressor and transcription factor, p53 interacts with hypoxic signals and the HIF family, selectively transcribing target genes that regulate angiogenesis, the tumor microenvironment, dormancy, metastasis, and recurrence, thus influencing cancer progression [31, 32]. Initially, we observed reduced p53 mRNA and protein levels and increased HIF-1α expression under hypoxic conditions. Moreover, liver-metastatic A549-LMs cells exhibited decreased p53 and elevated HIF-1α expression compared with parental A549 cells (Fig. 5B, C). Overexpression of p53 significantly suppressed SKA3 mRNA and protein expression, concurrently increasing OH-HIF-1α and reducing total HIF-1α protein levels, with no significant alterations in PHD2 or pVHL levels (Fig. 5D–F). To investigate whether p53 directly regulated SKA3 transcription, particularly under hypoxic conditions, we conducted bioinformatic predictions (JASPAR) and identified a potential p53-binding site (GCCTGCACCAACATA) within the SKA3 promoter (Fig. 5G). ChIP and electrophoretic mobility shift assays confirmed p53 binding to this region (Fig. 5H, I). Dual-luciferase reporter assays using wild-type and mutant SKA3 promoter constructs demonstrated significant repression of luciferase activity by p53 with the wild-type SKA3 promoter, but not with the mutant variant (Fig. 5J, K). Collectively, these results indicated that p53 transcriptionally represses SKA3. Additionally, we examined whether p53 has a specific regulatory relationship with SKA3 under hypoxic conditions. Studies have shown that under certain hypoxic conditions, p53’s transcriptional activity is inhibited [33–37]. To validate this possibility, A549 cells were exposed to varying hypoxic conditions induced by different concentrations of CoCl_2_ (0, 50, 100, and 200 μM). Dual-luciferase reporter assays indicated that increasing hypoxia progressively attenuated the inhibitory effect of p53 on the wild-type SKA3 promoter, whereas the effect on the mutant promoter remained unchanged (Fig. 5L).

Studies demonstrate that HIF-1α binds to HREs in the MDM2 promoter to increase MDM2 levels. MDM2 is an E3 ubiquitin ligase that specifically recognizes and ubiquitinates p53, marking it for proteasome-dependent degradation [33]. Exposure of A549 and A549-LMs cells to hypoxia markedly upregulated MDM2 mRNA levels compared with normoxia (Supplementary Fig. 5A), and this was mirrored at the protein level in both lines (Supplementary Fig. 5B). Similarly, SKA3 overexpression in A549 and A549-LMs cells elevated MDM2 protein relative to vector controls (Supplementary Fig. 5C), and this SKA3-driven induction was abolished by HIF-1α knockdown (Supplementary Fig. 5D, E), indicating that SKA3 acted via HIF-1α to regulate MDM2. We next assessed p53 ubiquitination by immunoprecipitating p53 and blotting for ubiquitin. Hypoxic treatment strongly enhanced the ubiquitin smear on p53 in A549 and A549-LMs cells (Supplementary Fig. 5F, G), reflecting increased p53 targeting for degradation. Similarly, SKA3 overexpression promoted p53 ubiquitination in both lines, and this effect was reversed by HIF-1α depletion (Supplementary Fig. 5H, I). Collectively, these data establish that SKA3 overexpression drives HIF-1α–dependent upregulation of MDM2, leading to increased p53 ubiquitination and degradation. In turn, hypoxia impairs p53-mediated repression of SKA3, resulting in elevated SKA3 expression. This feed-forward loop enables tumor cells to better tolerate hypoxic stress, thereby promoting liver metastasis progression.

### The biological function of SKA3 was regulated by p53 and depended on HIF-1α-mediated metabolic reprogramming

**Fig. 6:**
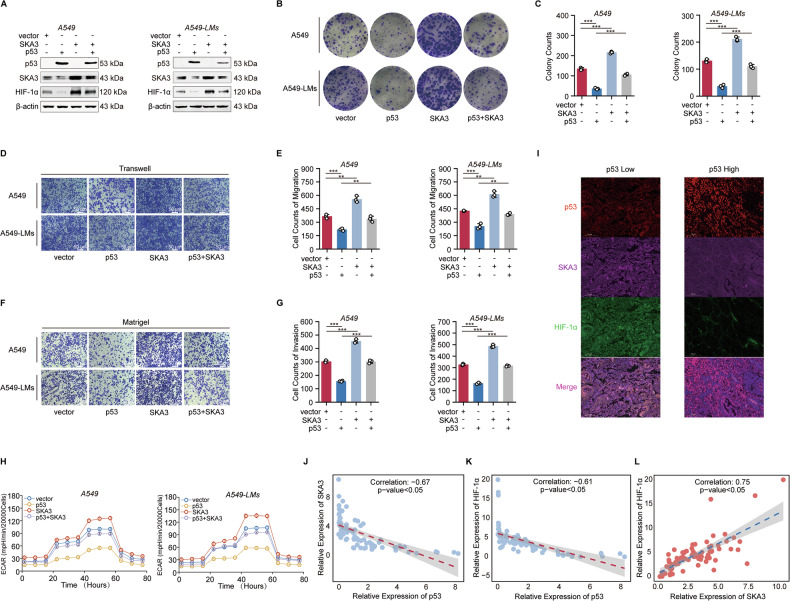
The biological function of SKA3 was regulated by p53 and depended on HIF-1α-mediated metabolic reprogramming. **A** WB analysis demonstrated that overexpression of p53 repressed the expression levels of SKA3 and HIF-1α, while DLX6 upregulation reversed the effects in A549 and A549-LMs cells. **B**, **C** Colony formation assays revealed that SKA3 overexpression rescued the inhibition of cell proliferation caused by p53 upregulation in A549 and A549-LMs cells. **D**–**G** Transwell and Matrigel experiments showed that overexpression of SKA3 could reverse the decreased migration and invasion ability of A549 and A549-LMs cells caused by p53 upregulation, and corresponding quantitative results were shown; Scale bar: 50 μm. **H** Overexpression of SKA3 could reverse the decrease in glycolytic capacity in A549 and A549-LMs cells caused by p53 upregulation. **I** Multiplex immunofluorescence staining of p53, SKA3, and HIF-1α in LUAD specimens. **J**–**L** Correlation analysis of p53, SKA3, and HIF-1α expression in 80 LUAD samples. *p < 0.05; **p < 0.01; ***p < 0.001; ns no significance.

To further clarify the regulatory relationship between p53 and SKA3 and its implications for metabolic reprogramming, we conducted rescue experiments. We overexpressed SKA3 and p53 individually or simultaneously in A549 and A549-LMs cell lines, alongside appropriate control groups. Western blot analysis demonstrated that SKA3 overexpression reversed the inhibitory effects of p53 on SKA3 and HIF-1α protein levels (Fig. 6A). Additionally, colony formation, transwell migration, and Matrigel invasion assays confirmed that SKA3 overexpression rescued the reduction in proliferation and invasive abilities induced by p53 overexpression (Fig. 6B–G). Seahorse assays and Lactate production further indicated that SKA3 overexpression reversed p53-mediated suppression of glycolytic metabolism in LUAD cells, along with the protein expression of key glycolytic enzymes (Fig. 6H; Supplementary Fig. 6A–D). Thus, the expression and biological function of SKA3 were regulated by p53. We subsequently performed multiplex immunohistochemistry to systematically assess p53, SKA3, and HIF-1α expression levels in 80 paired LUAD tissue samples. Compared with tissues showing high p53 expression, samples with low p53 expression exhibited significantly elevated levels of SKA3 and HIF-1α (Fig. 6I). Further correlation analyses revealed a significant negative correlation between p53 expression and SKA3/HIF-1α expression, while a significant positive correlation was identified between SKA3 and HIF-1α (Fig. 6J–L). In summary, our findings indicated that p53 exerts a negative regulatory effect on the SKA3/HIF-1α axis, thereby modulating tumor cell proliferation, invasion, and glycolytic metabolism in LUAD.

### A SKA3/PHD2/HIF-1α positive feedback loop reinforced hypoxia tolerance in LUAD

**Fig. 7:**
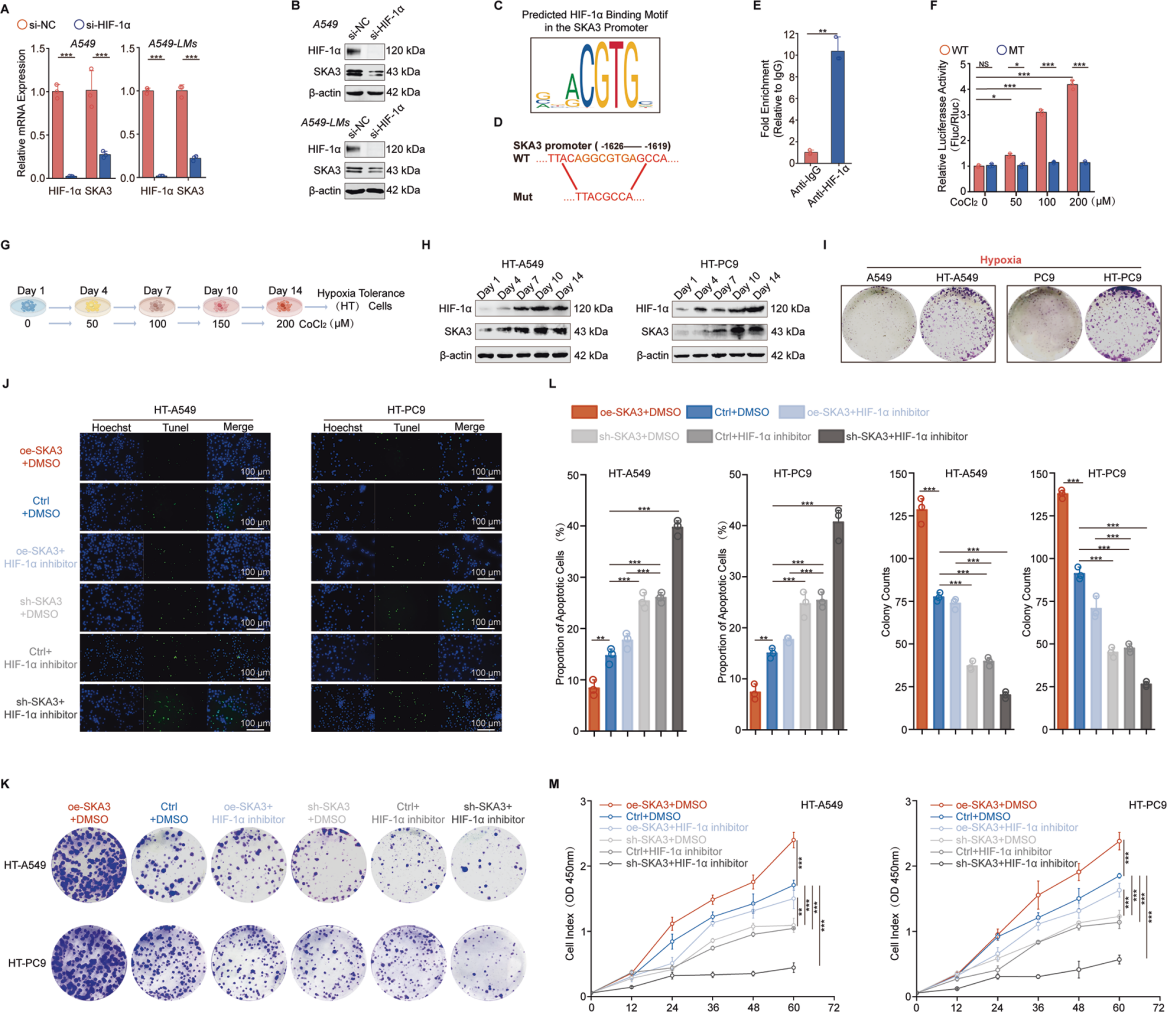
A SKA3/PHD2/HIF-1α positive feedback loop reinforced hypoxia tolerance in LUAD. **A**, **B** qRT-PCR and WB experiments demonstrated that knockdown of HIF-1α decreased SKA3 mRNA and protein expression. **C** JASPAR website (https://jaspar.genereg.net/) predicted HIF-1α binding sites in the SKA3 promoter region. **D** SKA3 wild-type (WT: TTACAGGCGTGAGCCA)/mutant-type (MT: TTACGCCA) promoter reporter plasmids were constructed based on predicted binding regions. **E** ChIP-qPCR experiments validated HIF-1 binding to the SKA3 promoter region; **F** dual-luciferase reporter gene experiments indicated enhanced transcriptional activation of SKA3 by HIF-1α with increasing severity of hypoxia. A549 cells with CoCl_2_ treatment (0, 50, 100, 200, 250 µM) were transfected with SKA3 promoter-WT or Mut reporters for 24 h. **G** Schematic diagram illustrating the establishment of hypoxia-tolerant (HT) cells. **H** WB analysis demonstrated that with increasing hypoxic severity, the expression levels of SKA3 and HIF-1α progressively increased in HT-A549 and HT-PC9 cells. **I** The established HT- cells and parental cells were cultured under hypoxic conditions. **J**–**M** HT cells with either SKA3 knockdown or overexpression, along with the control groups, were treated with an HIF-1α inhibitor (PX-478) or DMSO as a control. Subsequently, apoptosis levels were assessed via TUNEL staining (**J**), and cell proliferation was evaluated using colony formation (**K**) and CCK-8 (**M**) assays, and corresponding quantitative results were shown (**L**). *p < 0.05; **p < 0.01; ***p < 0.001; ns no significance.

HIF-1, as the functional complex form of nuclear HIF-1α, acts on the HREs of target genes, exerting transcriptional activation and inducing high expression of target genes under hypoxic conditions [38]. Hypoxia-tolerant LUAD cells exhibit a higher propensity for liver metastasis, accompanied by increased HIF-1α activation. We observed elevated expression levels of both SKA3 and HIF-1α under hypoxic conditions, suggesting that SKA3 might be a downstream target of HIF-1α. To explore their regulatory relationship, we utilized siRNA targeting HIF-1α, resulting in significant reductions in SKA3 mRNA and protein levels (Fig. 7A, B). Consistently, qRT-PCR showed that hypoxia markedly elevated HIF-1α mRNA and concomitantly upregulated SKA3 mRNA levels compared with normoxic controls (Supplementary Fig. 7A). Besides, pharmacological inhibition of HIF-1α with PX-478 (20 µM) during hypoxia markedly suppressed induction of both HIF-1α and SKA3 mRNA and protein levels (Supplementary Fig. 7B, C).

Subsequent transcription factor prediction analysis using JASPAR identified potential HIF-1α binding sites within the SKA3 promoter, specifically the GCGTGC sequence (Fig. 7C), which aligned with the typical HRE sequence (A/G) CGTG [39]. Further ChIP experiments confirmed that HIF-1α bound to the SKA3 promoter region (Fig. 7E). Therefore, we constructed luciferase reporter vectors containing either the wild-type or mutant SKA3 promoter regions targeting this interaction (Fig. 7D). Dual-luciferase assays showed that the transcriptional activity of plasmids with the wild-type SKA3 promoter increased with CoCl_2_ treatment, while the mutant group failed to induce luciferase activity (Fig. 7F).

Thus, our findings confirmed SKA3 as a novel hypoxia-inducible gene transcriptionally activated by HIF-1α, particularly under intensified hypoxic conditions. Consequently, the hypoxic tumor microenvironment promoted SKA3 upregulation through HIF-1α-mediated transcriptional activation. Importantly, hypoxia-induced SKA3 upregulation further stabilized HIF-1α protein, reinforcing the nuclear presence and transcriptional function of HIF-1α. This interaction formed a positive feedback loop, significantly enhancing glycolytic metabolism, particularly in hypoxia-tolerant LUAD cells with liver-metastatic potential.

To further investigate this mechanism, we established hypoxia-tolerant LUAD cell models in PC9 and A549 cells using a gradual hypoxic adaptation method. (Fig. 7G). Given the distinct p53 mutational backgrounds of A549 and PC9 cells, we further confirmed p53-mediated transcriptional repression of SKA3 in PC9 cells (Supplementary Fig. 7D–G). WB analysis demonstrated that SKA3 and HIF-1α levels increased in a time-dependent manner throughout the adaptation process, eventually stabilizing at higher expression levels (Fig. 7H). Hypoxia-tolerant cells exhibited significantly increased viability under hypoxic conditions compared to parental cells (Fig. 7I; Supplementary Fig. 7H–K). Inhibition of either SKA3 or HIF-1α markedly increased apoptosis and reduced proliferation in hypoxia-tolerant cells. Notably, ectopic overexpression of SKA3 partially restored hypoxia tolerance in cells where HIF-1α was suppressed. Furthermore, combined inhibition of SKA3 and HIF-1α nearly abolished cell viability under hypoxic conditions (Fig. 7J–M). Collectively, these findings suggested that the SKA3/PHD2/HIF-1α positive feedback loop significantly contributed to hypoxia tolerance, enabling LUAD cells to overcome hypoxic barriers and thereby facilitating liver metastasis.

## Discussion

Current research on LUAD liver metastasis primarily focuses on the interactions between tumor cells and the tumor microenvironment, particularly concerning tumor energy metabolism [40]. SKA3, recognized as an important oncogene, has been extensively studied in various primary tumors. SKA3 promotes the malignant progression of cervical cancer cells through the activation of the PI3K/AKT signaling pathway. Multiple studies have shown that SKA3 is closely related to the malignant progression and poor prognosis of various cancers, including melanoma, bladder cancer, and breast cancer [41–43]. Additionally, as a tumor metabolic regulatory gene, SKA3 can inhibit the ubiquitination and degradation of polo-like kinase 1 (PLK1) protein, resulting in the upregulation of glycolytic enzymes and enhanced glycolysis in laryngeal squamous cell carcinoma [44]. However, there are no studies on how SKA3 regulates tumor cell metabolism to promote cancer metastasis. This study confirmed that SKA3 acted as an oncogene in LUAD, significantly enhancing the proliferation and invasion of LUAD cells and being closely associated with poor clinical prognosis. For cancer cells to transition from colonization to an established metastatic niche, ensuring survival in the new microenvironment [19, 45]. In the context of liver metastasis, tumor cells that can efficiently utilize available resources to meet their energy demands are more likely to successfully colonize the liver [46]. Moreover, these cells must overcome the relatively hypoxic environment of the liver to thrive [11, 47, 48]. Further research has shown that knocking down SKA3 expression significantly inhibited LUAD overcoming the liver’s hypoxic barrier and facilitating the metabolic reprogramming of metastatic tumors to promote their colonization in the liver.

One of the most important features of metabolic reprogramming in tumors is increased reliance on glycolysis for energy production. High glycolysis not only provides energy but also supplies various nutrients for biosynthesis. Increased aerobic glycolysis facilitates tumor progression by conferring growth advantages, a drug-resistant phenotype, and enhanced metastatic potential to cancer cells [49]. HIF-1α signaling, as a typical response to hypoxia, is a key regulator under hypoxic conditions, increasing glycolysis and driving tumor development. HIF-1α is the main regulator of glucose metabolism, with its transcription upregulating glycolytic enzymes and membrane transport proteins, thereby increasing glucose flux and promoting glycolysis [50, 51]. SKA3 could stabilize HIF-1α protein expression, which in turn promoted glucose uptake, lactate secretion, and pyruvate production in LUAD cells, thereby increasing glycolysis and facilitating liver metastasis.

HIF-1α expression is associated with increased distant metastasis and decreased survival rates in various types of tumors [52]. HIF-1α interacts with VHL protein, activating the ubiquitin-proteasome system, leading to proteasomal degradation of HIF-1α. In renal clear cell carcinoma, Trim21 acts as an E3 ubiquitin ligase for HIF-1α, promoting its ubiquitination and degradation, thereby inhibiting glycolysis and suppressing the growth and metastasis of renal clear cell carcinoma [53]. BNIP3 deficiency induces NCOA4-mediated ferritinophagy, with PHD2-mediated HIF-1α downregulation inhibiting tumor glycolysis and growth in BNIP3-silenced melanoma cells [54]. PHD2 hydroxylates HIF-1α, which is necessary for VHL binding, leading to HIF-1α ubiquitination and degradation. The stability of HIF-1α is crucial for its sustained function. Although previous studies have reported that SKA3 interacted with PARP1/HIF-1α to promote cholangiocarcinoma proliferation [23], our findings did not reveal a direct interaction between SKA3 and HIF-1α in LUAD. We found that SKA3 inhibited PHD2 binding to HIF-1α, reducing OH-HIF-1α formation, preventing HIF-1α ubiquitination and degradation, thereby promoting HIF-1α transcriptional activation of downstream glycolytic enzymes and increasing glycolysis levels. Animal models showed that HIF-1α promoted LUAD liver metastasis. Reduced aerobic glycolysis in SKA3-silenced cells was secondary to decreased HIF-1α levels and the suppression of the overall transcriptional glycolytic program downstream. Further rescue experiments indicated that the biological function of SKA3 in promoting LUAD liver metastasis depended on HIF-1α-mediated metabolic reprogramming. Beyond metabolic reprogramming, our data reveal consistent enrichment of autophagy and cellular senescence pathways in metastatic LUAD cells and under hypoxia. This suggests that SKA3 and/or HIF-1α may modulate these stress-adaptive programs—either indirectly through metabolic stress or via parallel signaling branches—to promote metastatic cell survival and therapy resistance. Consequently, targeting autophagy or senescence alongside the SKA3/HIF-1α–glycolysis axis may offer a synergistic therapeutic approach in LUAD liver metastasis.

p53, a pivotal tumor suppressor and transcription factor, plays a crucial role in tumor suppression by responding to diverse stress signals, including hypoxia [55]. Upon exposure to such stressors, the half-life of the p53 protein significantly increases [56]. However, studies have indicated that hypoxia and HIF signaling may exert negative regulatory effects on both the levels and transcriptional activity of p53 [35–37]. Moreover, varying degrees of hypoxia can modulate p53 accumulation and its functional activity [57]. This investigation revealed that p53 strongly suppressed the transcription of SKA3. Under hypoxic conditions, there was a downregulation of p53 expression. With deeper hypoxia, p53’s transcriptional repression of SKA3 was weakened, inducing high expression of SKA3 under hypoxia. There is also a complex interaction between p53 and tumor hypoxia signals. Some studies have shown that p53 can downregulate HIF-1α protein levels via the E3 ubiquitin ligase Parkin [58]. Besides, hypoxia can activate CK2, which increases HIF-1α activity by reducing p53 levels [59]. High p53 expression inhibited SKA3 and HIF-1α levels, and rescue experiments further indicated that SKA3’s stabilizing effect on HIF-1α was regulated by p53.

HIF plays a critical role in cellular oxygen sensing by acting as a key transcription factors that stimulate the expression of downstream genes in response to hypoxic conditions. Upon accumulation, HIF-1α forms an active complex with ARNT (HIF-1β), which binds to HREs in gene promoters, recruiting other transcription factors and initiating a series of hypoxic adaptation responses in tissues and cells. For example, under hypoxic microenvironments, HIF-1α acts on NAT10 HRE sequences, activating its expression and promoting glucose metabolic reprogramming in gastric cancer cells [39]. Similarly, SKA3 exhibited heightened expression under hypoxia, facilitated by HIF-1α binding to HRE sequences within the SKA3 promoter region, thereby enhancing its transcriptional activity. Notably, increased SKA3 expression stabilized HIF-1α protein levels. Deeper hypoxia intensified this positive feedback loop, further enhancing HIF-1α‘s transcriptional activation of SKA3. Hypoxia-tolerant LUAD cells exhibited an increased propensity for liver metastasis. HIF-1α, the most critical gene activated under hypoxic conditions, was found to significantly reduce the liver metastasis of LUAD upon inhibition. This finding was consistent with our observation of elevated HIF-1α expression in liver-metastatic cells. Knocking down SKA3 combined with HIF-1α inhibitors (PX-478) suppressed tumor cell glucose metabolic reprogramming, weakened hypoxia tolerance, and synergistically inhibited LUAD liver metastasis.

This study presented compelling evidence supporting SKA3 as a potential biomarker for liver metastasis in LUAD, demonstrating its heightened expression as a hypoxia-inducible gene. Hypoxia-tolerant LUAD cells enhanced glycolysis through SKA3-mediated mechanisms, thereby facilitating their colonization in the liver. It elucidated the SKA3/PHD2/HIF-1α axis in driving metabolic reprogramming in LUAD and confirmed the synergistic therapeutic effect of silencing SKA3 combined with HIF-1α inhibitors on liver metastasis of LUAD. Furthermore, the research investigated the mechanism behind elevated SKA3 expression under hypoxic conditions, thereby offering promising avenues for targeted therapies against SKA3 in managing liver metastasis of LUAD.

## Supplementary information

Supplementary Figure 1

Supplementary Figure 2

Supplementary Figure 3

Supplementary Figure 4

Supplementary Figure 5

Supplementary Figure 6

Supplementary Figure 7

Supplementary Figure Legends

Supplementary Table 1

Supplementary Table 2

Supplementary Table 3

Supplementary Table 4

Original Western Blot

## Data Availability

The datasets used and analyzed during the current study are available from the corresponding author on reasonable request.
