# Supplementary material for: SKA3-mediated hypoxia tolerance and metabolic reprogramming promote liver metastasis in lung adenocarcinoma
Source: Cell Death Dis. 2025 Nov 26;17(1):65. doi: 10.1038/s41419-025-08270-z (PMC12827483; doi:10.1038/s41419-025-08270-z)
Supplement: Supplementary file 8 — Supplementary Figure Legends [file 41419_2025_8270_MOESM8_ESM.docx]

**Supplementary Figure Legends**

**Supplementary Figure 1 | SKA3 knockdown inhibited the function of LUAD cells under hypoxic conditions.** (A) Transcriptome sequencing clustering of A549 vs. A549-hypoxia and A549 vs. A549-LMs was shown. (B-D) q-PCR and WB analysis showing no significant difference in the relative mRNA or protein expression of NEFL and CA9 between A549 and A549-LMs cells. (E) SKA3 expression is significantly higher in liver metastatic NSCLC tissues compared to primary NSCLC. (F) No significant difference in SKA3 expression is observed between primary NSCLC and brain metastatic tissues in the GSE200563 dataset. (G) No significant difference in SKA3 expression is found between primary NSCLC and bone metastatic tissues in the GSE225208 dataset. (H) Quantitative results of Colony formation, EdU staining, Transwell, and Matrigel assays were obtained from A549 and A549-LMs cells following SKA3 knockdown. (I) RTCA assay showed knockdown of SKA3 inhibited the proliferation ability of A549 and A549-LMs cells under hypoxic conditions. (J) Colony formation assay showed colony numbers of sh-SKA3 group were less than control group under hypoxic conditions, and corresponding quantitative results were shown (N). (K) EdU assay showed the positive signal of sh-SKA3 group were less than control group under hypoxic conditions, and corresponding quantitative results were shown (N); Scale bar: 100 μm. (L-M) Transwell (L) and Matrigel (M) assays revealed that knockdown of SKA3 inhibited the migration and invasion of A549 and A549-LMs cells under hypoxic conditions, quantitative results were shown (N); Scale bar: 50 μm. (O-P) Tissue microarray analysis (TMA) of 80 pairs of LUAD clinical samples indicated that SKA3 was highly expressed in tumor tissues. (Q) SKA3 was highly expressed in LUAD tumor tissue compared to the paired normal tissue in TCGA dataset. (R) SKA3 was significantly upregulated in T3-T4 tumors when compared with T1-T2 tumors. (S) Kaplan-Meier (KM) plots showed high SKA3 expression level associated with the poor overall survival of patients with LUAD. **p* < 0.05; ***p* < 0.01; ****p* < 0.001; ns, no significance.

**Supplementary Figure 2 | SKA3 enhanced glycolysis of A549-LMs cells by stabilizing HIF-1α protein.** (A) Schematic representation of the amino acid interaction sites between SKA3 and PHD2. (B-C) IP experiments using anti-SKA3 antibodies were performed under both normal and hypoxic conditions, followed by immunoblotting to verify the binding of SKA3 to HIF-1α. (D) WB analysis indicated increased OH-HIF-1α and decreased HIF-1α levels, with no significant change in PHD2 and VHL protein levels following SKA3 knockdown in A549-LMs cells. (E) WB analysis indicated decreased OH-HIF-1α and increased HIF-1α levels after SKA3 overexpression, without significant changes in PHD2 and VHL protein levels in A549-LMs cells. (F) Knockdown of SKA3 did not affect HIF-1α protein levels in A549-LMs cells treated with the proteasome inhibitor MG132 (25 μM), the numbers represented relative HIF-1α expression. (G) Knockdown of SKA3 accelerated degradation of HIF-1α protein in A549-LMs cells treated with the transcription inhibitor CHX (200 μg/mL), the numbers represented relative HIF-1α expression. (H) IP assays demonstrated the interaction of SKA3 and PHD2 was weakened in A549-LMs cells after transfected with SKA3 siRNA. (I) IP assays demonstrated the interaction of HIF-1α and PHD2 was strengthened in A549-LMs cells after transfected with SKA3 siRNA. MG132 (25 μM) was added to inhibit HIF-1α degradation. (J) PLA showing protein interactions in A549-LMs cells. The upper panel displays the interaction between SKA3 and PHD2 (red puncta), while the lower panel shows the interaction between PHD2 and HIF-1α (red puncta) in both control (si-Ctrl) and SKA3 knockdown (si-SKA3) cells. Nuclei are stained with DAPI (blue). Scale bars represent 100 µm. (K) IP assays revealed that HIF-1α ubiquitination levels were increased in A549-LMs cells after SKA3 siRNA transfection. MG132 (25 μM) was added to inhibit HIF-1α degradation. (L) IP assays followed by immunoblotting (IB) with an antibody specific for K48‐linked ubiquitin chains (Ub‐K48), SKA3 knockdown increased Ub‐K48 of HIF‐1α in A549‐LM cells.

**Supplementary Figure 3 |** **Knockdown of SKA3 Suppressed Nuclear HIF-1α Levels.** (A) Nuclear-cytoplasmic fractionation experiments indicated that SKA3 knockdown led to a reduction in nuclear HIF-1α protein levels, with β-Tubulin used as the nuclear reference and Lamin B as the cytoplasmic reference. (B-C) Immunofluorescence indicated that knockdown of SKA3 (Green) resulted in a decrease in nuclear HIF-1α (Red) in A549 and A549-LMs cells, nuclei were counterstained with DAPI (blue); Scale bar: 20 μm.

**Supplementary Figure 4 |** **Validation of SKA3 knockdown and overexpression in A549 cells and impact of SKA3 overexpression on glycolytic enzyme expression in mouse liver metastases.** (A) Relative SKA3 mRNA expression in A549 cells stably expressing SKA3 shRNA (sh-SKA3) or non-targeting control shRNA (sh-Ctrl), and in cells stably overexpressing SKA3 (SKA3) or empty vector control (vector). (B) WB analysis of SKA3 protein in the same stable knockdown and overexpression lines. (C-E) LUAD liver metastasis models were constructed using SKA3 knockdown, overexpression or control PC9 cells. The mice were administered the HIF-1α inhibitor (PX-478) by gavage, while the control group received DMSO. Bioluminescence imaging was performed after 4 weeks (C), and corresponding fluorescence quantitative (D), HE staining results were shown. *p < 0.05; **p < 0.01; ***p < 0.001; ns, no significance. (F) Immunoblot of HIF-1α and downstream glycolytic enzymes (HK2, PKM2, GLUT3, PDK1, LDHA) in protein lysates from mouse liver metastases generated by vector-control (vec-1 to vec-5) or SKA3-overexpressing (oe-1 to oe-5) cells. **p* < 0.05; ***p* < 0.01; ****p* < 0.001; ns, no significance.

**Supplementary Figure 5 | Hypoxia induced MDM2 expression and promoted p53 ubiquitination in a HIF-1α-dependent manner.** (A) qRT-PCR showing a significant increase in MDM2 mRNA under hypoxia versus normoxia in both A549 and A549-LMs cells. (B) WB of whole-cell lysates from A549 and A549-LMs cells cultured under normoxia or hypoxia, demonstrating elevated MDM2 protein levels upon hypoxic treatment. (C) SKA3 overexpression markedly increased MDM2 protein compared with vector control in A549 and A549-LMs cells. (D-E) A549 and A549-LMs cells were transfected with empty vector or SKA3 expression plasmid, together with non-targeting siRNA (si-NC) or HIF-1α siRNA (si-HIF-1α). Immunoblotting showed that SKA3 overexpression markedly increased MDM2 protein levels compared to vector control, and this increase was abolished when HIF-1α was knocked down. (F-G) Immunoprecipitation of p53 followed by immunoblotting for ubiquitin in A549 and A549-LMs cells under normoxia or hypoxia. (H-I) In SKA3-overexpressing A549 and A549-LMs cells, HIF-1α knockdown (si-HIF-1α) reduces the SKA3-driven increase in p53 ubiquitination. Input controls confirm MDM2 induction and equal p53 and β-actin loading. Data are representative of at least three independent experiments. (H-I) A549 and A549-LMs cells were transfected with empty vector or SKA3 expression plasmid, together with si-NC or si-HIF-1α. Cell lysates were immunoprecipitated with anti-p53 and blotted for ubiquitin. SKA3 overexpression markedly increased p53 ubiquitination, which was abolished by HIF-1α knockdown. Input controls showed MDM2 induction, unchanged total p53, and equal β-actin loading. **p* < 0.05; ***p* < 0.01; ****p* < 0.001; ns, no significance.

**Supplementary Figure 6 |** **p53 Inhibited SKA3-Mediated Enhancement of Glycolysis.** (A-B) Lactate production assays in A549 and A549-LMs cells under different conditions: vector, SKA3 overexpression, and p53 expression. (C-D) WB analysis of glycolytic enzymes in A549 and A549-LMs cells with stable expression of SKA3 and p53. The expression levels of HK2, PKM2, GLUT3, LDHA, and PDK1 are shown. Actin was used as a loading control. **p* < 0.05; ***p* < 0.01; ****p* < 0.001; ns, no significance.

**Supplementary Figure 7 | HIF-1α inhibition abrogated hypoxia-induced upregulation of SKA3 in A549 and A549-LMs cells.** (A) qRT-PCR analysis showing that hypoxic exposure significantly increases HIF-1α and SKA3 mRNA levels relative to normoxia in both A549 and A549-LMs cells. (B) Treatment with the HIF-1α inhibitor PX-478 (20 µM) for 24 h under hypoxia markedly reduces HIF-1α and SKA3 mRNA levels compared with DMSO control in both cell lines. (C) Representative WB of A549 and A549-LMs cell lysates after 24 h of normoxia or hypoxia, with DMSO or PX-478. (D) WB analysis of PC9 cells transfected with empty vector or SKA3 expression plasmid, with or without p53 overexpression. Overexpression of p53 markedly reduced both HIF-1α and SKA3 protein levels. (E) qRT-PCR showed that p53 overexpression increases p53 transcript abundance and concurrently decreases SKA3 and HIF-1α mRNA levels. (F) ChIP using anti-p53 antibody demonstrates ~10-fold enrichment of p53 at the SKA3 promoter relative to IgG control, indicating direct promoter binding. (G) Luciferase reporter assay in PC9 cells co-transfected with wild-type (WT) or p53-response-element-mutant (MT) SKA3 promoter constructs and either vector or p53 expression plasmid. p53 overexpression significantly suppresses WT promoter activity, whereas repression of the MT promoter is substantially attenuated. (H-K) The established HT-cells and parental cells were cultured under hypoxic conditions, CCK-8 assays (H-I) show increased cell proliferation in HT-A549 and HT-PC9 cells compared to their parental counterparts; (J-K) TUNEL staining reveals elevated apoptosis (green) in A549 and PC9 cells, with fewer apoptotic cells observed in HT-A549 and HT-PC9 cells under hypoxia. **p* < 0.05; ***p* < 0.01; ****p* < 0.001; ns, no significance.
