# Supplementary material for: SKA3-mediated hypoxia tolerance and metabolic reprogramming promote liver metastasis in lung adenocarcinoma
Source: Cell Death Dis. 2025 Nov 26;17(1):65. doi: 10.1038/s41419-025-08270-z (PMC12827483; doi:10.1038/s41419-025-08270-z)
Supplement: Supplementary file 9 — Supplementary Table 1 [file 41419_2025_8270_MOESM9_ESM.docx]

**Supplementary Table S1. Sequences of shRNAs, and siRNAs used for experiments in this study**

| Gene | Oligo | Sequences |
| --- | --- | --- |
| SKA3#1 | Sense | CCACGUAGUCCACAACUUUTT |
|  | Antisense | AAAGUUGUGGACUACGUGGTT |
| SKA3#2 | Sense | CCACCUACCAAACAAUCACUA |
|  | Antisense | UAGUGAUUGUUUGGUAGGUGG |
| HIF-1α#1 | Sense | GGAAAUGAGAGAAAUGCUUTT |
|  | Antisense | AAGCAUUUCUCUCAUUUCCTC |
| HIF-1α#2 | Sense | GCUGGAGACACAAUCAUAUTT |
|  | Antisense | AUAUGAUUGUGUCUCCAGCGG |
| Ctrl-NC | Sense | UUCUCCGAACGUGUCACGUdTdT |
|  | Antisense | ACGUGACACGUUCGGAGAAdTdT |
