# Supplementary material for: SKA3-mediated hypoxia tolerance and metabolic reprogramming promote liver metastasis in lung adenocarcinoma
Source: Cell Death Dis. 2025 Nov 26;17(1):65. doi: 10.1038/s41419-025-08270-z (PMC12827483; doi:10.1038/s41419-025-08270-z)
Supplement: Supplementary file 10 — Supplementary Table 2 [file 41419_2025_8270_MOESM10_ESM.docx]

**Supplementary Table S2. Sequences of primers used for experiments in this study**

| Gene name | Primers | Sequecce | Application |
| --- | --- | --- | --- |
| SKA3 | Forward | TACACGAGCAAGAAGCCATTAAC | qRT-PCR |
|  | Reverse | GGATACGATGTACCGCTCAAGT |  |
| p53 | Forward | ATGAAGCTCCCAGAATGC | qRT-PCR |
|  | Reverse | GGGCCGCCGGTGTAG |  |
| PHD2 | Forward | AGGCGATAAGATCACCTGGAT | qRT-PCR |
|  | Reverse | TTCGTCCGGCCATTGATTTTG |  |
| HIF-1α | Forward | TCACCACAGGACAGTACAGGATGC | qRT-PCR |
|  | Reverse | CCAGCAAAGTTAAAGCATCAGGTTCC |  |
| HK2 | Forward | AAGGCTTCAAGGCATCTG | qRT-PCR |
|  | Reverse | CCACAGGTCATCATAGTTCC |  |
| PKM2 | Forward | GTGCGAGCCTCAAGTCACTCCACA | qRT-PCR |
|  | Reverse | TATAAGAAGCCTCCACGCTGCCCA |  |
| GLUT3 | Forward | AACCAGCTGGGCATCGTTGTTGG | qRT-PCR |
|  | Reverse | GCCACAATAAACCAGGGAATGGG |  |
| LDHA | Forward | AGCCCGATTCCGTTACCT | qRT-PCR |
|  | Reverse | CACCAGCAACATTCATTCCA |  |
| PDK1 | Forward | CTGTGATACGGATCAGAAACCG | qRT-PCR |
|  | Reverse | TCCACCAAACAATAAAGAGTGCT |  |
| ACTB | Forward | GAAATCGTGCGTGACATTAA | qRT-PCR |
|  | Reverse | AAGGAAGGCTGGAAGAGTG |  |
| SKA3 | Forward | CTCTGCCTCTGAGGTTCA | ChIP-PCR |
| SKA3 | Reverse | AGGAGTTCAAGACCAGCTT | ChIP-PCR |
