# Supplementary material for: SKA3-mediated hypoxia tolerance and metabolic reprogramming promote liver metastasis in lung adenocarcinoma
Source: Cell Death Dis. 2025 Nov 26;17(1):65. doi: 10.1038/s41419-025-08270-z (PMC12827483; doi:10.1038/s41419-025-08270-z)
Supplement: Supplementary file 11 — Supplementary Table 3 [file 41419_2025_8270_MOESM11_ESM.docx]

**Supplemental Table S3.** Information on antibodies used in this study

| **Antibody** | **WB** | **IHC/IF** | **IP/ChIP** | **Specificity** | **Company (catalog number)** |
| --- | --- | --- | --- | --- | --- |
| SKA3 | 1:2000 | 5 µg/ml | 5µg | Rabbit polyclonal | Abcam (ab186003) |
| PHD2 | 1:1000 | -- | 1:50 | Rabbit monoclonal | Proteintech (66589-1-lg) |
| p53 | 1:1000 | 1 µg/ml | 5µg | Rabbit monoclonal | Abcam (ab1101) |
| HIF-1α | 1:500 | 2 µg/ml | 1:30 | Rabbit monoclonal | Abcam (ab308433) |
| OH-HIF-1α | 1:1000 | -- | -- | Rabbit monoclonal | Abcam (ab308637) |
| Ubiquitin | 1:500 | -- | -- | Mouse monoclonal | CST (3936S) |
| HK2 | 1:5000 | -- | -- | Mouse polyclonal | Proteintech (66974-1-lg) |
| GLUT3 | 1:2000 | -- | -- | Rabbit monoclonal | Proteintech (20403-1-AP) |
| PKM2 | 1:1000 | -- | -- | Rabbit monoclonal | Proteintech (15822-1-AP) |
| PDK1 | 1:1000 | -- | -- | Rabbit monoclonal | Abcam (ab202468) |
| LDHA | 1:2000 | -- | -- | Rabbit monoclonal | Proteintech (19987-1-AP) |
| β-Actin | 1:4000 | -- | -- | Rabbit monoclonal | Proteintech (20536-1-AP) |
| β-Tublin | 1:2000 | -- | -- | Rabbit monoclonal | Proteintech (10094-1-AP) |
| Lamin B1 | 1:5000 | -- | -- | Rabbit monoclonal | Proteintech (12987-1-AP) |
| Ub-K48 | 1:1000 | -- | -- | Rabbit monoclonal | Abclonal (A3606) |
| VHL | 1:1000 | -- | -- | Rabbit monoclonal | Abcam (ab270968) |
| PHD2 | -- | 1:200 | -- | Mouse polyclonal | Proteintech (66589-1-Ig) |

Abbreviations: WB, Western blot; IHC, Immunohistochemistry;

IF, Immunofluorescence；ChIP，Chromatin Immunoprecipitation；

IP，Immunoprecipitation；
