# Supplementary material for: SKA3-mediated hypoxia tolerance and metabolic reprogramming promote liver metastasis in lung adenocarcinoma
Source: Cell Death Dis. 2025 Nov 26;17(1):65. doi: 10.1038/s41419-025-08270-z (PMC12827483; doi:10.1038/s41419-025-08270-z)
Supplement: Supplementary file 12 — Supplementary Table 4 [file 41419_2025_8270_MOESM12_ESM.docx]

**Supplemental Table 4. Identification of SKA3-Associated Proteins by Immunoprecipitation and Mass Spectrometry**

| **SKA3_IP_MS_Screening** | | |
| --- | --- | --- |
| **Accession** | **Gene** | **Mw(kDa)** |
| P05783 | KRT18 | 48.058 |
| P63261 | ACTG1 | 41.793 |
| P35579 | MYH9 | 226.532 |
| Q562R1 | ACTBL2 | 42.003 |
| P19105 | MYL12A | 19.794 |
| P07355 | ANXA2 | 38.604 |
| P60660 | MYL6 | 16.93 |
| O43707 | ACTN4 | 104.854 |
| Q15149 | PLEC | 531.791 |
| P09493 | TPM1 | 32.709 |
| P00367 | GLUD1 | 61.398 |
| P16403 | H1-2 | 21.365 |
| P0DP25 | CALM3 | 16.838 |
| P06753 | TPM3 | 32.95 |
| Q9NYL9 | TMOD3 | 39.595 |
| P01857 | IGHG1 | 36.106 |
| P62424 | RPL7A | 29.996 |
| P35580 | MYH10 | 228.999 |
| P62750 | RPL23A | 17.695 |
| P21333 | FLNA | 280.739 |
| P11142 | HSPA8 | 70.898 |
| Q13813 | SPTAN1 | 284.539 |
| O00159 | MYO1C | 121.682 |
| P38646 | HSPA9 | 73.681 |
| P61247 | RPS3A | 29.945 |
| P07951 | TPM2 | 32.851 |
| P11940 | PABPC1 | 70.671 |
| Q02878 | RPL6 | 32.728 |
| O75369 | FLNB | 278.164 |
| P62899 | RPL31 | 14.463 |
| Q7Z406 | MYH14 | 227.871 |
| P67936 | TPM4 | 28.522 |
| Q9GZT9 | EGLN1/PHD2 | 46.021 |
| P46821 | MAP1B | 270.634 |
| P39019 | RPS19 | 16.061 |
| Q86V81 | ALYREF | 26.888 |
| P42766 | RPL35 | 14.551 |
| P06748 | NPM1 | 32.575 |
| P30050 | RPL12 | 17.819 |
| Q9UPQ0 | LIMCH1 | 121.867 |
| P61353 | RPL27 | 15.798 |
| O43795 | MYO1B | 131.985 |
| P46783 | RPS10 | 18.898 |
| P18077 | RPL35A | 12.538 |
| Q01082 | SPTBN1 | 274.609 |
| Q00610 | CLTC | 191.615 |
| P62979 | RPS27A | 17.965 |
| P62701 | RPS4X | 29.598 |
| Q8WWI1 | LMO7 | 192.696 |
| P62269 | RPS18 | 17.719 |
| P38159 | RBMX | 42.332 |
| P50914 | RPL14 | 23.432 |
| P62888 | RPL30 | 12.784 |
| P47755 | CAPZA2 | 32.949 |
| P35637 | FUS | 53.426 |
| P11021 | HSPA5 | 72.333 |
| P46779 | RPL28 | 15.748 |
| P49207 | RPL34 | 13.293 |
| P47756 | CAPZB | 31.35 |
| Q13347 | EIF3I | 36.502 |
| Q92522 | H1-10 | 22.487 |
| Q9P0K7 | RAI14 | 110.041 |
| Q9Y608 | LRRFIP2 | 82.171 |
| P26373 | RPL13 | 24.261 |
| P0DMV8 | HSPA1A | 70.052 |
| O15372 | EIF3H | 39.93 |
| P52907 | CAPZA1 | 32.923 |
| P19338 | NCL | 76.614 |
| Q6WCQ1 | MPRIP | 116.533 |
| Q00839 | HNRNPU | 90.584 |
| Q16643 | DBN1 | 71.429 |
| P23396 | RPS3 | 26.688 |
| Q9UNX3 | RPL26L1 | 17.256 |
| P12270 | TPR | 267.293 |
| Q05682 | CALD1 | 93.231 |
| P16401 | H1-5 | 22.58 |
| P62081 | RPS7 | 22.127 |
| P46778 | RPL21 | 18.565 |
| P62861 | FAU | 14.39 |
| P46013 | MKI67 | 358.694 |
| Q9Y3U8 | RPL36 | 12.254 |
| P62277 | RPS13 | 17.222 |
| Q13162 | PRDX4 | 30.54 |
| Q12792 | TWF1 | 40.283 |
| P62280 | RPS11 | 18.431 |
| P63010 | AP2B1 | 104.553 |
| P18124 | RPL7 | 29.226 |
| O95425 | SVIL | 247.746 |
| P23246 | SFPQ | 76.149 |
| P09651 | HNRNPA1 | 38.747 |
| Q9ULV4 | CORO1C | 53.249 |
| P05387 | RPLP2 | 11.665 |
| P63173 | RPL38 | 8.218 |
| P40429 | RPL13A | 23.577 |
| P62851 | RPS25 | 13.742 |
| Q14247 | CTTN | 61.586 |
| Q96C19 | EFHD2 | 26.697 |
| P61513 | RPL37A | 10.275 |
| P22626 | HNRNPA2B1 | 37.43 |
| P60903 | S100A10 | 11.203 |
| P07305 | H1-0 | 20.863 |
| Q99880 | H2BC13 | 13.952 |
| P60228 | EIF3E | 52.221 |
| Q16698 | DECR1 | 36.068 |
| P51991 | HNRNPA3 | 39.595 |
| P62273 | RPS29 | 6.677 |
| O00303 | EIF3F | 37.564 |
| P62906 | RPL10A | 24.831 |
| Q9Y262 | EIF3L | 66.727 |
| O15144 | ARPC2 | 34.333 |
| P55884 | EIF3B | 92.482 |
| P61978 | HNRNPK | 50.976 |
| Q92614 | MYO18A | 233.115 |
| Q14152 | EIF3A | 166.569 |
| Q02543 | RPL18A | 20.762 |
| O95793 | STAU1 | 63.182 |
| P15880 | RPS2 | 31.324 |
| P60866 | RPS20 | 13.373 |
| P61158 | ACTR3 | 47.371 |
| P62910 | RPL32 | 15.86 |
| A6NFX1 | MFSD2B | 53.743 |
| P12814 | ACTN1 | 103.058 |
| P62263 | RPS14 | 16.273 |
| P67809 | YBX1 | 35.924 |
| P23528 | CFL1 | 18.502 |
| P08708 | RPS17 | 15.55 |
| P05386 | RPLP1 | 11.514 |
| O95782 | AP2A1 | 107.546 |
| P05388 | RPLP0 | 34.274 |
| P18621 | RPL17 | 21.397 |
| Q14517 | FAT1 | 506.273 |
| Q7L2H7 | EIF3M | 42.503 |
| Q27J81 | INF2 | 135.624 |
| Q9UHB6 | LIMA1 | 85.226 |
| Q9Y224 | RTRAF | 28.068 |
| Q9Y2W1 | THRAP3 | 108.666 |
| Q99613 | EIF3C | 105.344 |
| O75821 | EIF3G | 35.611 |
| Q07666 | KHDRBS1 | 48.227 |
| P62847 | RPS24 | 15.423 |
| Q5SSJ5 | HP1BP3 | 61.207 |
| P06576 | ATP5F1B | 56.56 |
| P26599 | PTBP1 | 57.221 |
| P49458 | SRP9 | 10.112 |
| Q13045 | FLII | 144.751 |
| Q8WVK7 | SKA2 | 14.188 |
| P83731 | RPL24 | 17.779 |
| O15143 | ARPC1B | 40.95 |
| Q69YQ0 | SPECC1L | 124.544 |
| Q8IX90 | SKA3 | 46.359 |
| Q12906 | ILF3 | 95.338 |
| P25705 | ATP5F1A | 59.751 |
| P32969 | RPL9 | 21.863 |
| P25054 | APC | 311.646 |
| P62244 | RPS15A | 14.84 |
| O15145 | ARPC3 | 20.547 |
| P08670 | VIM | 53.652 |
| P07910 | HNRNPC | 33.67 |
| P62917 | RPL8 | 28.025 |
| Q92841 | DDX17 | 80.272 |
| Q8TEM1 | NUP210 | 205.111 |
| P17936 | IGFBP3 | 31.674 |
| Q8NC51 | SERBP1 | 44.965 |
| Q9NZI8 | IGF2BP1 | 63.481 |
| Q5VTE0 | EEF1A1P5 | 50.185 |
| P39023 | RPL3 | 46.109 |
| Q8IVT2 | MISP | 75.357 |
| P62913 | RPL11 | 20.252 |
| O00422 | SAP18 | 17.561 |
| P49411 | TUFM | 49.542 |
| P49757 | NUMB | 70.804 |
| O14974 | PPP1R12A | 115.281 |
| P62753 | RPS6 | 28.681 |
| Q9H307 | PNN | 81.628 |
| Q9UN86 | G3BP2 | 54.121 |
| O15371 | EIF3D | 63.973 |
| P62318 | SNRPD3 | 13.916 |
| P10412 | H1-4 | 21.865 |
| P38919 | EIF4A3 | 46.871 |
| Q9Y239 | NOD1 | 107.691 |
| P62249 | RPS16 | 16.445 |
| Q96PK6 | RBM14 | 69.492 |
| Q06830 | PRDX1 | 22.11 |
| Q96HS1 | PGAM5 | 32.004 |
| P80723 | BASP1 | 22.693 |
| O75955 | FLOT1 | 47.355 |
| P25398 | RPS12 | 14.515 |
| O15231 | ZNF185 | 73.525 |
| Q12905 | ILF2 | 43.062 |
| P09496 | CLTA | 27.077 |
| Q13283 | G3BP1 | 52.164 |
| P62136 | PPP1CA | 37.512 |
| Q9Y618 | NCOR2 | 273.657 |
| Q9Y3I0 | RTCB | 55.21 |
| Q01844 | EWSR1 | 68.478 |
| P35268 | RPL22 | 14.787 |
| P12236 | SLC25A6 | 32.866 |
| P57088 | TMEM33 | 27.978 |
| P09497 | CLTB | 25.19 |
| Q92804 | TAF15 | 61.83 |
| Q96HA7 | TONSL | 150.929 |
| P05976 | MYL1 | 21.145 |
| P31943 | HNRNPH1 | 49.229 |
| P61160 | ACTR2 | 44.761 |
| Q9BUJ2 | HNRNPUL1 | 95.739 |
| P14923 | JUP | 81.745 |
| P84098 | RPL19 | 23.466 |
| P40939 | HADHA | 83 |
| Q9H0D6 | XRN2 | 108.582 |
| Q9BQF6 | SENP7 | 119.658 |
| P62805 | H4C1 | 11.367 |
| Q04637 | EIF4G1 | 175.491 |
| P51114 | FXR1 | 69.721 |
| Q13492 | PICALM | 70.755 |
| P68371 | TUBB4B | 49.831 |
| Q9NZB2 | FAM120A | 121.888 |
| Q09666 | AHNAK | 629.101 |
| P17844 | DDX5 | 69.148 |
| P09669 | COX6C | 8.781 |
| Q96IZ0 | PAWR | 36.568 |
| P16402 | H1-3 | 22.35 |
| Q07020 | RPL18 | 21.634 |
| P62241 | RPS8 | 24.205 |
| Q13151 | HNRNPA0 | 30.841 |
| P14649 | MYL6B | 22.764 |
| Q14011 | CIRBP | 18.648 |
| P46781 | RPS9 | 22.591 |
| P27348 | YWHAQ | 27.764 |
| P62857 | RPS28 | 7.841 |
| P61313 | RPL15 | 24.146 |
| P06730 | EIF4E | 25.097 |
| P63244 | RACK1 | 35.077 |
| Q9Y520 | PRRC2C | 316.911 |
| P59998 | ARPC4 | 19.667 |
| Q9Y2U8 | LEMD3 | 99.997 |
| Q9UNZ5 | C19orf53 | 10.577 |
| E9PRG8 | C11orf98 | 14.234 |
| P36578 | RPL4 | 47.697 |
| Q96CW1 | AP2M1 | 49.655 |
| Q9UJZ1 | STOML2 | 38.534 |
| P42677 | RPS27 | 9.461 |
| Q8WWM7 | ATXN2L | 113.374 |
| O43809 | NUDT21 | 26.227 |
| Q07157 | TJP1 | 195.459 |
| Q9GZT3 | SLIRP | 12.349 |
| Q86W42 | THOC6 | 37.535 |
| P46777 | RPL5 | 34.363 |
| P20042 | EIF2S2 | 38.388 |
| Q9UPN3 | MACF1 | 838.308 |
| P10809 | HSPD1 | 61.055 |
| Q3MHD2 | LSM12 | 21.701 |
| P61421 | ATP6V0D1 | 40.329 |
| P27635 | RPL10 | 24.577 |
| O00571 | DDX3X | 73.243 |
| P46940 | IQGAP1 | 189.252 |
| P52298 | NCBP2 | 18.001 |
| Q9BZF9 | UACA | 162.505 |
| P13073 | COX4I1 | 19.577 |
| P10606 | COX5B | 13.696 |
| Q6P1L8 | MRPL14 | 15.948 |
| P35221 | CTNNA1 | 100.071 |
| P53680 | AP2S1 | 17.018 |
| Q9NXV6 | CDKN2AIP | 61.125 |
| B2RPK0 | HMGB1P1 | 24.238 |
| Q92499 | DDX1 | 82.432 |
| Q15427 | SF3B4 | 44.386 |
| Q6ZS10 | CLEC17A | 42.935 |
| Q15717 | ELAVL1 | 36.092 |
| P63162 | SNRPN | 24.614 |
| P02743 | APCS | 25.387 |
| P62316 | SNRPD2 | 13.527 |
| Q9UQ80 | PA2G4 | 43.787 |
| P42830 | CXCL5 | 11.972 |
| P09661 | SNRPA1 | 28.416 |
| Q9BQE3 | TUBA1C | 49.895 |
| O14639 | ABLIM1 | 87.688 |
| Q9Y4I1 | MYO5A | 215.405 |
| Q15746 | MYLK | 210.715 |
| P55084 | HADHB | 51.294 |
| O00425 | IGF2BP3 | 63.705 |
| O75477 | ERLIN1 | 39.171 |
| Q8NBS9 | TXNDC5 | 47.629 |
| Q2VIR3 | EIF2S3B | 51.229 |
| P62140 | PPP1CB | 37.187 |
| A8TX70 | COL6A5 | 289.926 |
| P35222 | CTNNB1 | 85.497 |
| Q9Y2R9 | MRPS7 | 28.134 |
| Q15390 | MTFR1 | 37.001 |
| Q12797 | ASPH | 85.863 |
| Q96FV9 | THOC1 | 75.666 |
| P62314 | SNRPD1 | 13.282 |
| P35611 | ADD1 | 80.955 |
| Q9BPX5 | ARPC5L | 16.941 |
| Q9ULE4 | FAM184B | 121.044 |
| P05198 | EIF2S1 | 36.112 |
| O75083 | WDR1 | 66.194 |
| P23284 | PPIB | 23.743 |
| O60506 | SYNCRIP | 69.603 |
| Q15233 | NONO | 54.232 |
| P61326 | MAGOH | 17.164 |
| P52597 | HNRNPF | 45.672 |
| Q86XN6 | ZNF761 | 87.716 |
| Q15366 | PCBP2 | 38.58 |
| P62854 | RPS26 | 13.015 |
| Q5JSZ5 | PRRC2B | 242.967 |
| Q15393 | SF3B3 | 135.577 |
| Q9C0C2 | TNKS1BP1 | 181.796 |
| P62266 | RPS23 | 15.808 |
| P46782 | RPS5 | 22.876 |
| P46776 | RPL27A | 16.561 |
| Q8NCA5 | FAM98A | 55.273 |
| P20674 | COX5A | 16.762 |
| Q9Y5L4 | TIMM13 | 10.5 |
| P84090 | ERH | 12.259 |
| Q14677 | CLINT1 | 68.259 |
| Q9UBQ5 | EIF3K | 25.06 |
| Q9NYF8 | BCLAF1 | 106.122 |
| P07437 | TUBB | 49.671 |
| Q9NZT1 | CALML5 | 15.893 |
| Q7RTV0 | PHF5A | 12.405 |
| Q8NB91 | FANCB | 97.726 |
| Q86UE4 | MTDH | 63.837 |
| P00352 | ALDH1A1 | 54.862 |
| P62829 | RPL23 | 14.865 |
| P63241 | EIF5A | 16.832 |
| O75665 | OFD1 | 116.671 |
| Q13310 | PABPC4 | 70.783 |
| P36542 | ATP5F1C | 32.996 |
| P01834 | IGKC | 11.765 |
| Q6YHK3 | CD109 | 161.689 |
| Q6NZI2 | CAVIN1 | 43.476 |
| Q9Y281 | CFL2 | 18.737 |
| Q9Y6R0 | NUMBL | 64.891 |
| P62937 | PPIA | 18.012 |
| O75822 | EIF3J | 29.062 |
| O15020 | SPTBN2 | 271.325 |
| O94973 | AP2A2 | 103.96 |
| P36873 | PPP1CC | 36.984 |
| P60842 | EIF4A1 | 46.154 |
| Q6NXT2 | H3-5 | 15.214 |
| Q14160 | SCRIB | 174.915 |
| P14868 | DARS1 | 57.136 |
| P00390 | GSR | 56.257 |
| Q92747 | ARPC1A | 41.569 |
| Q9NR48 | ASH1L | 332.79 |
| Q9BWJ5 | SF3B5 | 10.135 |
| Q03135 | CAV1 | 20.472 |
| Q9UKV8 | AGO2 | 97.208 |
| P09038 | FGF2 | 30.77 |
| P26232 | CTNNA2 | 105.313 |
| Q08211 | DHX9 | 140.958 |
| P34932 | HSPA4 | 94.331 |
| Q9P2E9 | RRBP1 | 152.456 |
| Q9Y2Q9 | MRPS28 | 20.843 |
| Q9NR30 | DDX21 | 87.344 |
| Q9Y2W7 | KCNIP3 | 29.231 |
| P63104 | YWHAZ | 27.745 |
| Q6IBS0 | TWF2 | 39.548 |
| Q9UKM9 | RALY | 32.463 |
| Q99714 | HSD17B10 | 26.923 |
| P63208 | SKP1 | 18.658 |
| P27816 | MAP4 | 121.005 |
| Q99729 | HNRNPAB | 36.225 |
| Q14444 | CAPRIN1 | 78.366 |
| Q15287 | RNPS1 | 34.208 |
| P47914 | RPL29 | 17.752 |
| Q9Y2R5 | MRPS17 | 14.502 |
| P37108 | SRP14 | 14.57 |
| P26196 | DDX6 | 54.417 |
| P26641 | EEF1G | 50.119 |
| P22695 | UQCRC2 | 48.443 |
| Q9Y5S9 | RBM8A | 19.889 |
| P06702 | S100A9 | 13.242 |
| P83111 | LACTB | 60.694 |
| P13797 | PLS3 | 70.811 |
| P14866 | HNRNPL | 64.133 |
| O43920 | NDUFS5 | 12.518 |
| P38606 | ATP6V1A | 68.304 |
| Q9HC36 | MRM3 | 47.02 |
| Q00059 | TFAM | 29.097 |
| Q9UJV9 | DDX41 | 69.838 |
| O75531 | BANF1 | 10.059 |
| P62304 | SNRPE | 10.804 |
| Q14764 | MVP | 99.327 |
| P16615 | ATP2A2 | 114.757 |
| P56545 | CTBP2 | 48.945 |
| Q6NYC8 | PPP1R18 | 67.943 |
| Q32MZ4 | LRRFIP1 | 89.253 |
| Q9Y230 | RUVBL2 | 51.157 |
| Q13765 | NACA | 23.384 |
| Q96AG4 | LRRC59 | 34.93 |
| P63267 | ACTG2 | 41.877 |
| Q9Y265 | RUVBL1 | 50.228 |
| Q99623 | PHB2 | 33.296 |
| P81605 | DCD | 11.284 |
| Q14103 | HNRNPD | 38.434 |
| P61604 | HSPE1 | 10.932 |
| P22087 | FBL | 33.784 |
| P60953 | CDC42 | 21.259 |
| Q9UJC5 | SH3BGRL2 | 12.326 |
| P56537 | EIF6 | 26.599 |
| P06733 | ENO1 | 47.169 |
| P09012 | SNRPA | 31.28 |
| Q96JP2 | MYO15B | 167.088 |
| P48047 | ATP5PO | 23.277 |
| Q9NRW1 | RAB6B | 23.462 |
| P62258 | YWHAE | 29.174 |
| Q16531 | DDB1 | 126.968 |
| P14854 | COX6B1 | 10.192 |
| Q9UBI6 | GNG12 | 8.006 |
| Q04828 | AKR1C1 | 36.788 |
| Q86U42 | PABPN1 | 32.749 |
| Q92665 | MRPS31 | 45.318 |
| P68133 | ACTA1 | 42.051 |
| O60573 | EIF4E2 | 28.362 |
| Q08495 | DMTN | 45.514 |
| O95994 | AGR2 | 19.979 |
| Q9Y232 | CDYL | 66.482 |
| Q13868 | EXOSC2 | 32.789 |
| Q9Y383 | LUC7L2 | 46.514 |
| P05121 | SERPINE1 | 45.06 |
| P18859 | ATP5PF | 12.588 |
| O75368 | SH3BGRL | 12.774 |
| P62306 | SNRPF | 9.725 |
| P10599 | TXN | 11.738 |
| Q9BRJ6 | C7orf50 | 22.083 |
| O75348 | ATP6V1G1 | 13.758 |
| Q9BQG0 | MYBBP1A | 148.855 |
| P61981 | YWHAG | 28.303 |
| P84103 | SRSF3 | 19.33 |
| Q9UEY8 | ADD3 | 79.155 |
| Q9H7E9 | C8orf33 | 24.993 |
| P08195 | SLC3A2 | 67.994 |
| P55735 | SEC13 | 35.541 |
| Q96DI7 | SNRNP40 | 39.311 |
| O94905 | ERLIN2 | 37.84 |
| P04844 | RPN2 | 69.284 |
| P56385 | ATP5ME | 7.933 |
| Q9Y3D9 | MRPS23 | 21.771 |
| P24534 | EEF1B2 | 24.764 |
| P09234 | SNRPC | 17.394 |
| O60220 | TIMM8A | 10.998 |
| P14406 | COX7A2 | 9.396 |
| Q96BD8 | SKA1 | 29.484 |
| O75380 | NDUFS6 | 13.712 |
| Q14847 | LASP1 | 29.717 |
| Q7Z417 | NUFIP2 | 76.121 |
| P49448 | GLUD2 | 61.434 |
| Q6I9Y2 | THOC7 | 23.743 |
| Q9P035 | HACD3 | 43.16 |
| Q9BRT6 | LLPH | 15.225 |
| Q96J01 | THOC3 | 38.772 |
| Q15007 | WTAP | 44.244 |
| Q5VZF2 | MBNL2 | 40.518 |
| P82664 | MRPS10 | 22.999 |
| O75607 | NPM3 | 19.344 |
| P0DTE2 | IGHV8-51-1 | 13.081 |
| Q9BZK7 | TBL1XR1 | 55.595 |
| O00746 | NME4 | 20.659 |
| Q6P161 | MRPL54 | 15.819 |
| Q96SB3 | PPP1R9B | 89.334 |
| Q9Y295 | DRG1 | 40.542 |
| Q9BY77 | POLDIP3 | 46.089 |
| Q9Y676 | MRPS18B | 29.396 |
| P31689 | DNAJA1 | 44.868 |
| P29401 | TKT | 67.878 |
| P04792 | HSPB1 | 22.783 |
| P11413 | G6PD | 59.257 |
| Q99848 | EBNA1BP2 | 34.852 |
| P21281 | ATP6V1B2 | 56.501 |
| C9JLW8 | MCRIP1 | 10.92 |
| P35080 | PFN2 | 15.046 |
| P51648 | ALDH3A2 | 54.848 |
| Q96EY1 | DNAJA3 | 52.489 |
| P24539 | ATP5PB | 28.909 |
| P0DI83 | RAB34 | 21.118 |
| Q9BY49 | PECR | 32.544 |
| Q8N684 | CPSF7 | 52.05 |
| P31930 | UQCRC1 | 52.646 |
| Q8NI27 | THOC2 | 182.775 |
| Q9NPE3 | NOP10 | 7.706 |
| Q06787 | FMR1 | 71.174 |
| Q9BSD7 | NTPCR | 20.713 |
| Q96BP2 | CHCHD1 | 13.475 |
| O15234 | CASC3 | 76.278 |
| Q9NQT4 | EXOSC5 | 25.249 |
| Q12860 | CNTN1 | 113.32 |
| Q7Z7K6 | CENPV | 29.946 |
| O75947 | ATP5PD | 18.491 |
| P30419 | NMT1 | 56.806 |
| Q9H0U6 | MRPL18 | 20.577 |
| P28331 | NDUFS1 | 79.468 |
| P25205 | MCM3 | 90.981 |
| O43837 | IDH3B | 42.184 |
| Q8N8Y2 | ATP6V0D2 | 40.426 |
| O75643 | SNRNP200 | 244.508 |
| Q9NX58 | LYAR | 43.634 |
| Q9ULJ8 | PPP1R9A | 123.342 |
| P29083 | GTF2E1 | 49.452 |
| P61964 | WDR5 | 36.588 |
| P30838 | ALDH3A1 | 50.395 |
| O95810 | CAVIN2 | 47.173 |
| P50552 | VASP | 39.83 |
| Q96EL3 | MRPL53 | 12.107 |
| P21291 | CSRP1 | 20.567 |
| P08238 | HSP90AB1 | 83.264 |
| Q10713 | PMPCA | 58.253 |
| P62308 | SNRPG | 8.496 |
| P61026 | RAB10 | 22.541 |
| P35232 | PHB1 | 29.804 |
| Q6P2Q9 | PRPF8 | 273.6 |
| Q0VG06 | FAAP100 | 93.433 |
| P04083 | ANXA1 | 38.714 |
| P04406 | GAPDH | 36.053 |
| O95831 | AIFM1 | 66.901 |
| P27824 | CANX | 67.568 |
| P19404 | NDUFV2 | 27.392 |
| Q07065 | CKAP4 | 66.022 |
| O43143 | DHX15 | 90.933 |
| Q5RKV6 | EXOSC6 | 28.235 |
| P11388 | TOP2A | 174.385 |
| P33992 | MCM5 | 82.286 |
| P04843 | RPN1 | 68.569 |
| Q15084 | PDIA6 | 48.121 |
| Q96EY4 | TMA16 | 23.864 |
| Q13148 | TARDBP | 44.74 |
| P39656 | DDOST | 50.801 |
| Q7RTP6 | MICAL3 | 224.295 |
| O75439 | PMPCB | 54.366 |
| Q9P2X7 | DELEC1 | 7.542 |
| O43324 | EEF1E1 | 19.811 |
| P13639 | EEF2 | 95.338 |
| P27695 | APEX1 | 35.555 |
| P50990 | CCT8 | 59.621 |
| P61769 | B2M | 13.715 |
| P78417 | GSTO1 | 27.566 |
| Q08380 | LGALS3BP | 65.331 |
| Q6DKI1 | RPL7L1 | 29.669 |
| Q6WKZ4 | RAB11FIP1 | 137.167 |
| Q7Z7K0 | CMC1 | 12.49 |
| Q86V48 | LUZP1 | 120.275 |
| Q96KQ7 | EHMT2 | 132.37 |
| Q9NUL7 | DDX28 | 59.581 |
| Q9P032 | NDUFAF4 | 20.266 |
| Q9UKD2 | MRTO4 | 27.56 |
| Q9Y6A4 | CFAP20 | 22.774 |
| H7BZ55 | CROCC2 | 185.641 |
| O00203 | AP3B1 | 121.32 |
| O00206 | TLR4 | 95.68 |
| O75691 | UTP20 | 318.385 |
| P04432 | IGKV1D-39 | 12.737 |
| P0CG38 | POTEI | 121.282 |
| P38435 | GGCX | 87.561 |
| P41218 | MNDA | 45.836 |
| P51693 | APLP1 | 72.176 |
| P60709 | ACTB | 41.737 |
| Q05048 | CSTF1 | 48.358 |
| Q07021 | C1QBP | 31.362 |
| Q14643 | ITPR1 | 313.929 |
| Q14721 | KCNB1 | 95.878 |
| Q58F21 | BRDT | 107.954 |
| Q6P4A8 | PLBD1 | 63.255 |
| Q6ZUB1 | SPATA31E1 | 157.136 |
| Q86T65 | DAAM2 | 123.499 |
| Q86TY3 | ARMH4 | 84.173 |
| Q8IUR7 | ARMC8 | 75.509 |
| Q8N2W9 | PIAS4 | 56.504 |
| Q8N392 | ARHGAP18 | 74.977 |
| Q8TES7 | FBF1 | 125.446 |
| Q8TEY7 | USP33 | 106.727 |
| Q92974 | ARHGEF2 | 111.543 |
| Q96MR6 | CFAP57 | 144.961 |
| Q96PX6 | CCDC85A | 59.976 |
| Q9BRZ2 | TRIM56 | 81.488 |
| Q9BZ11 | ADAM33 | 87.739 |
| Q9H7D0 | DOCK5 | 215.309 |
| Q9NP81 | SARS2 | 58.283 |
| Q9NQ32 | C11orf16 | 51.609 |
| Q9NW15 | ANO10 | 76.329 |
| Q9Y5V0 | ZNF706 | 8.498 |
| Q9Y679 | AUP1 | 45.787 |
