# Supplementary material for: SKA3-mediated hypoxia tolerance and metabolic reprogramming promote liver metastasis in lung adenocarcinoma
Source: Cell Death Dis. 2025 Nov 26;17(1):65. doi: 10.1038/s41419-025-08270-z (PMC12827483; doi:10.1038/s41419-025-08270-z)
Supplement: Supplementary file 13 — Original Western Blot [file 41419_2025_8270_MOESM13_ESM.docx]

# Figure 1H

SKA3 43kDa


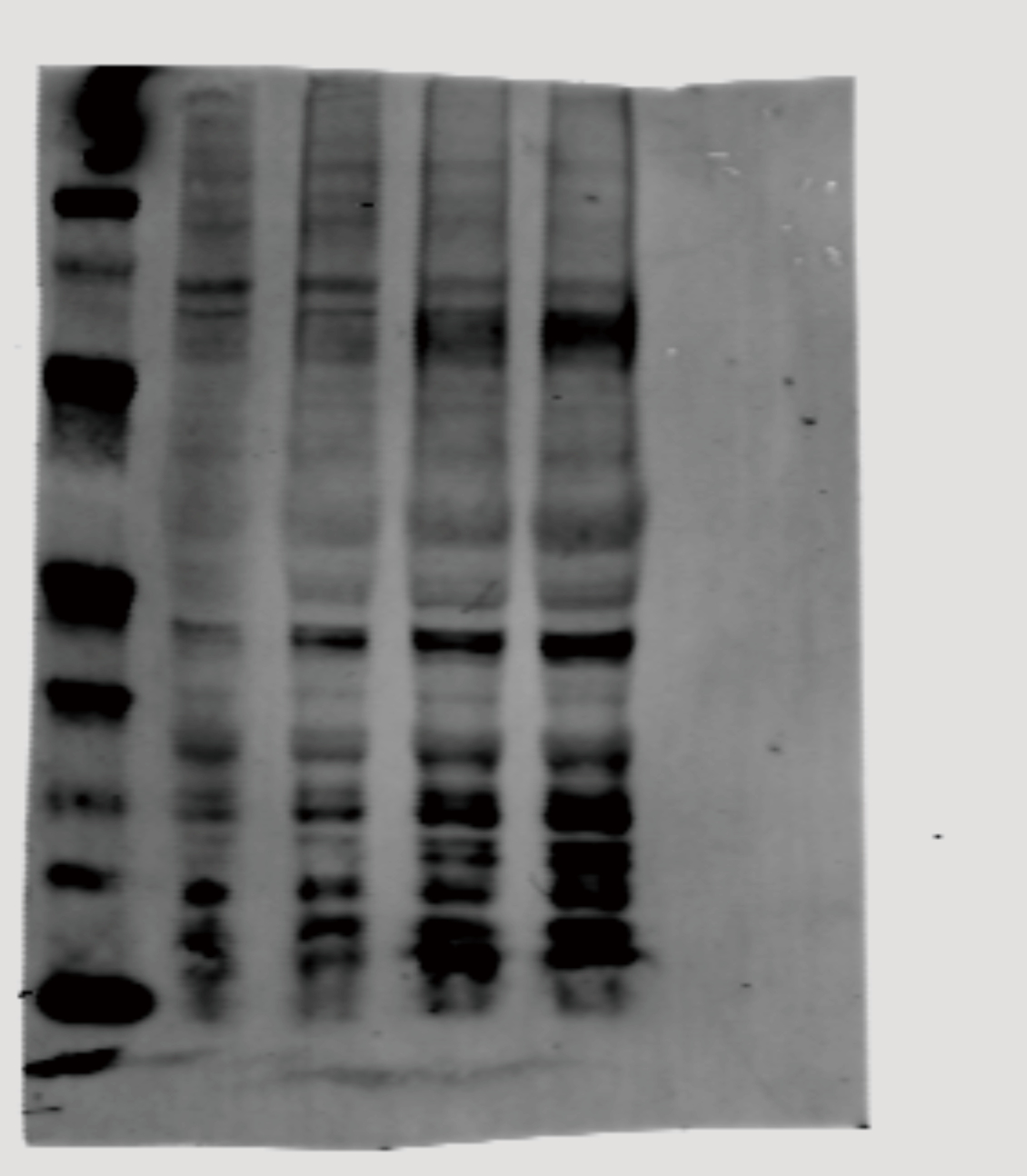

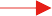


Actin 42 kDa

55 kDa

55 kDa

40 kDa


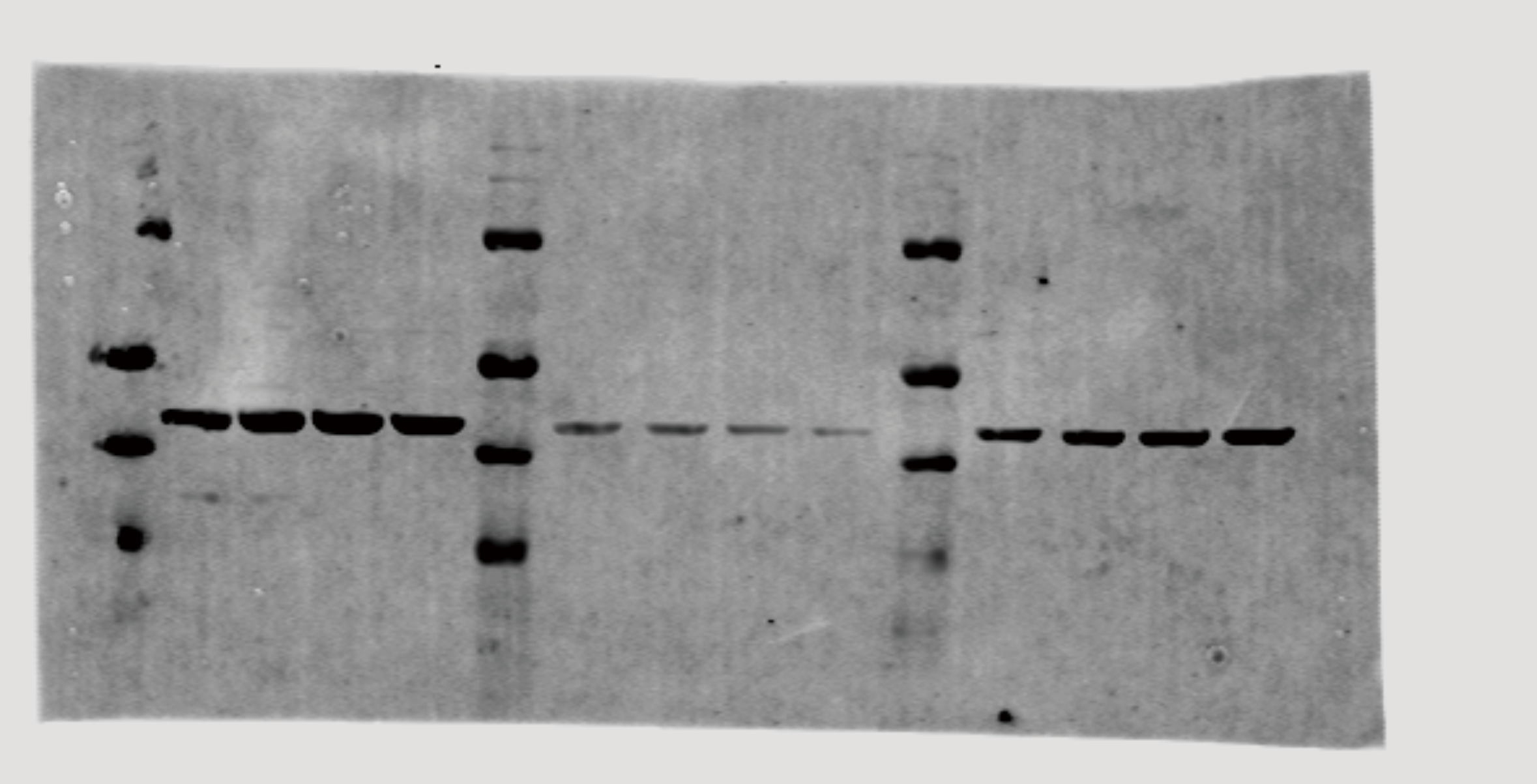

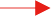

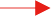


# Figure 2G A549

SKA3 43kDa

HK2 102kDa

PKM2 58kDa GLUT3 54kDa LDHA 37kDa PDK1 47 kDa


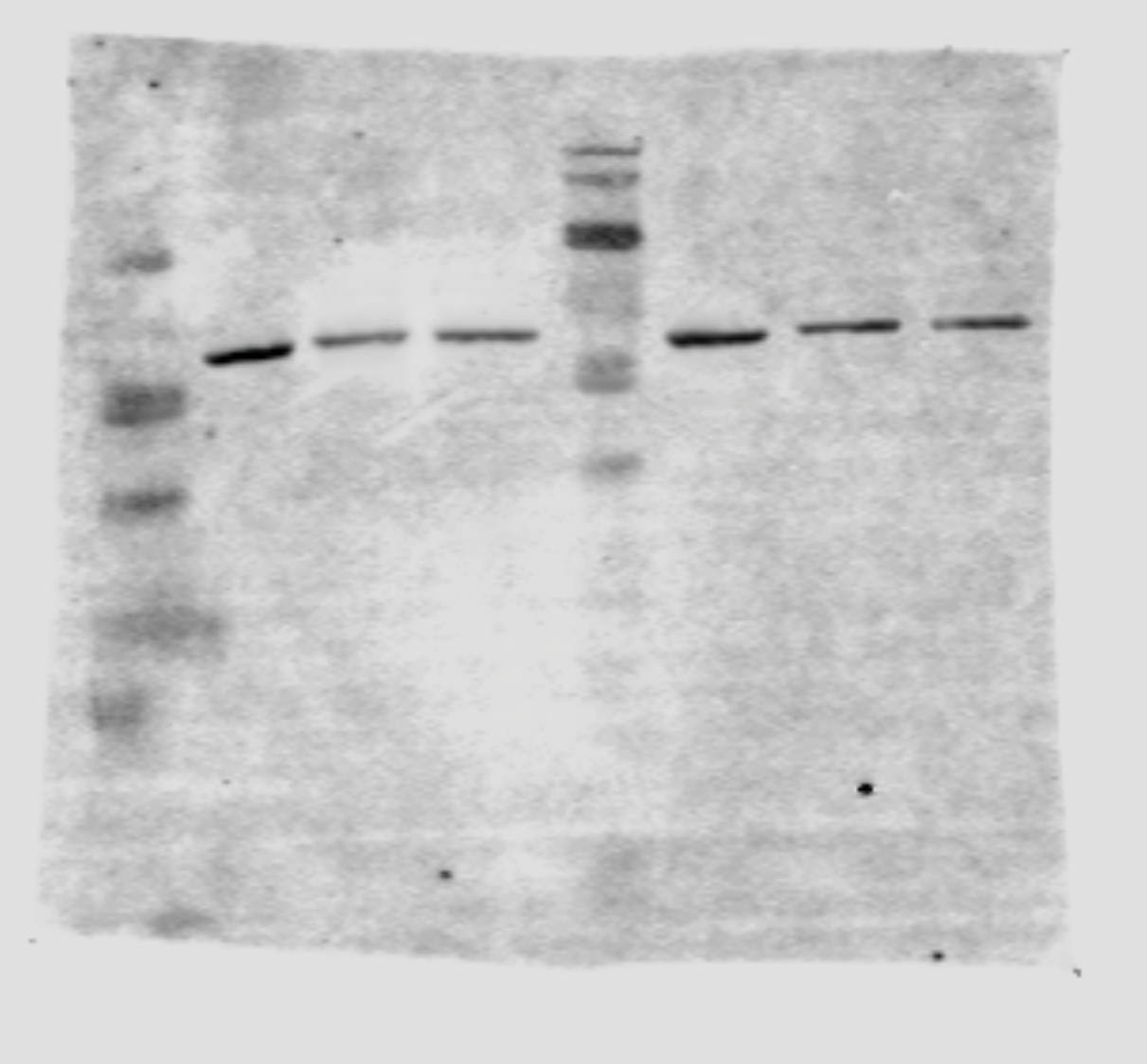

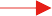

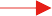


Actin 42 kDa

130 kDa


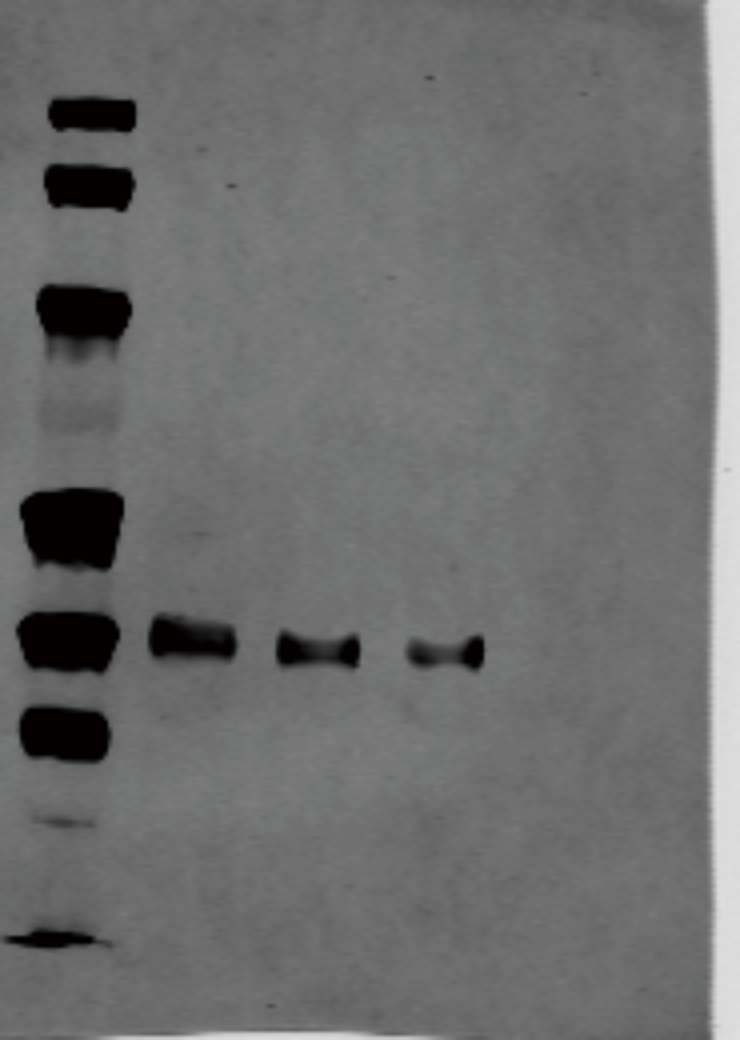

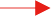

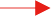

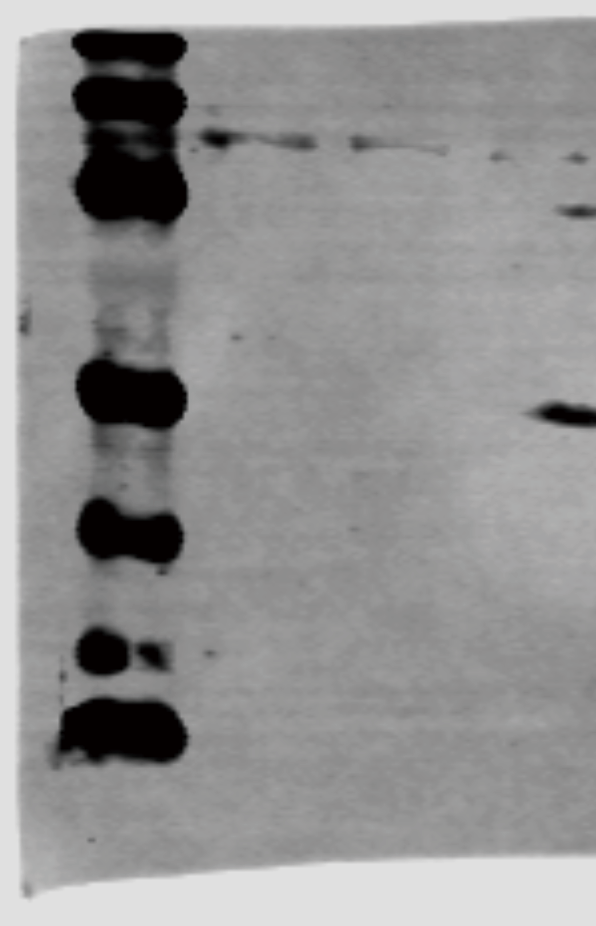

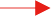

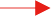


100 kDa

70kDa

70kDa


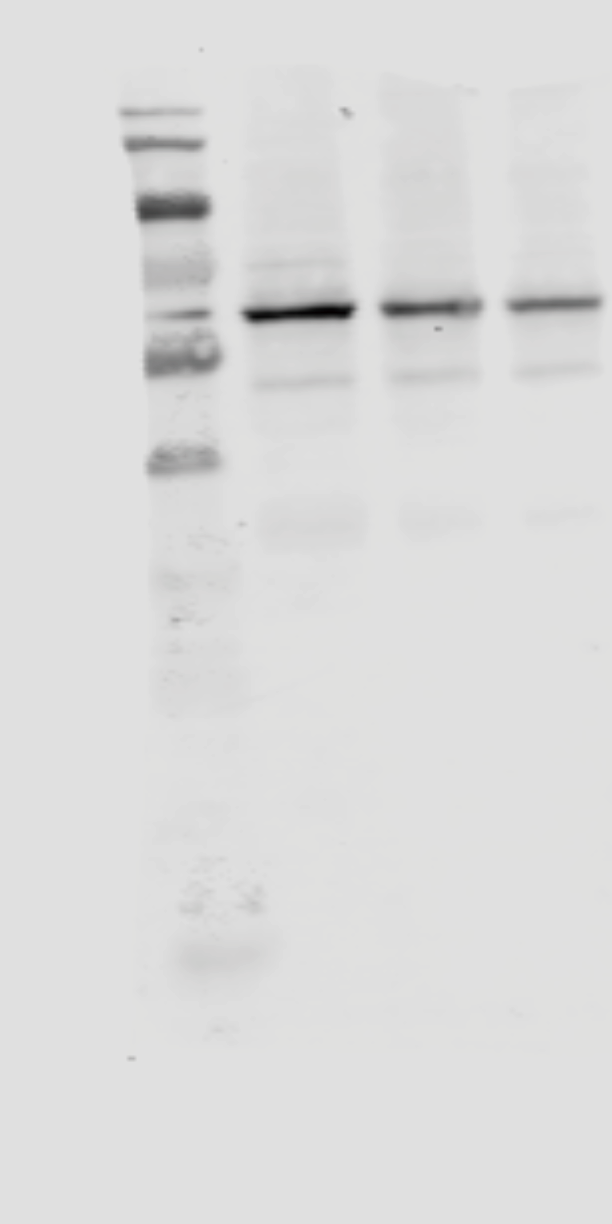

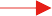

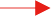

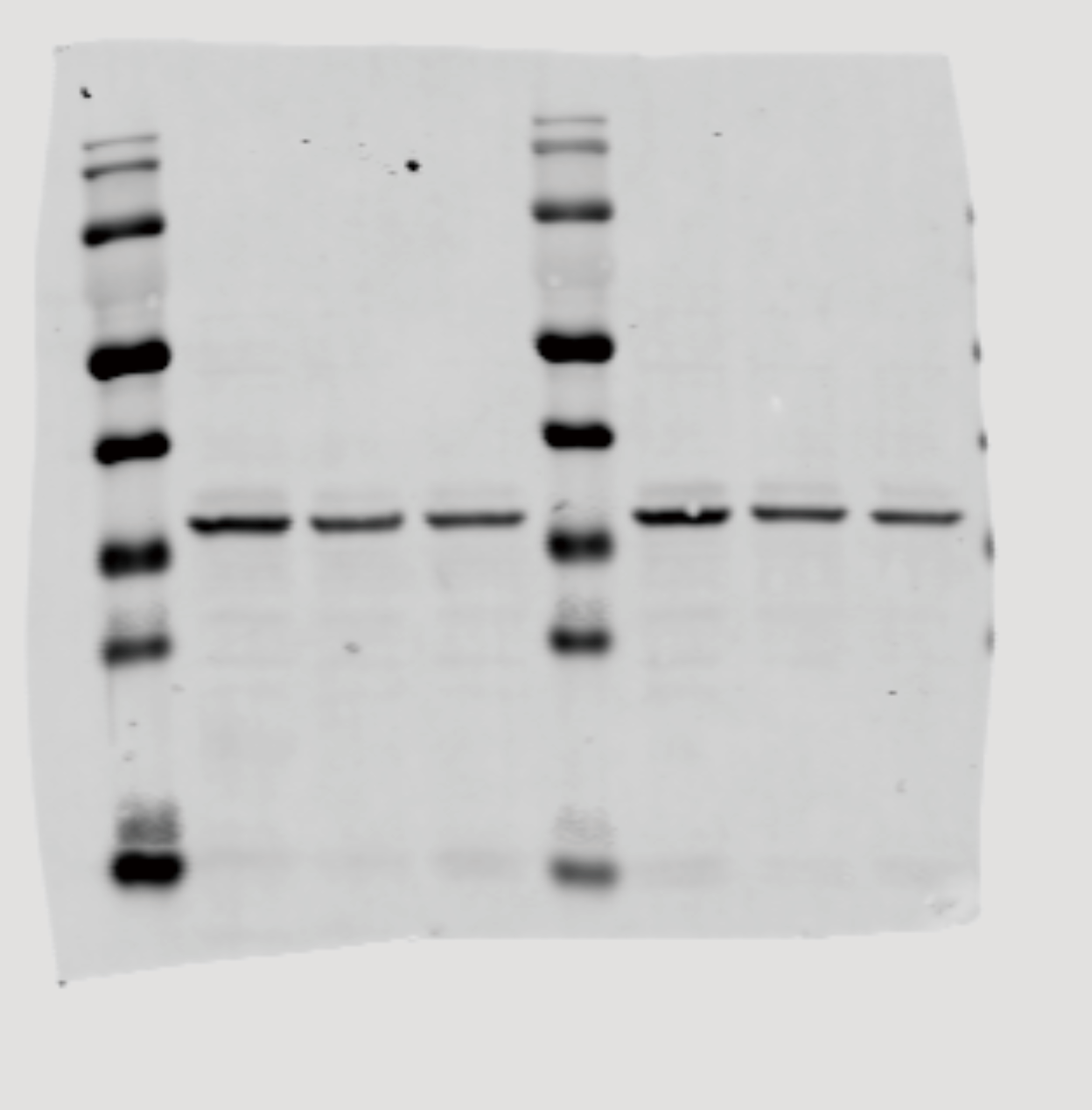

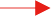

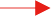

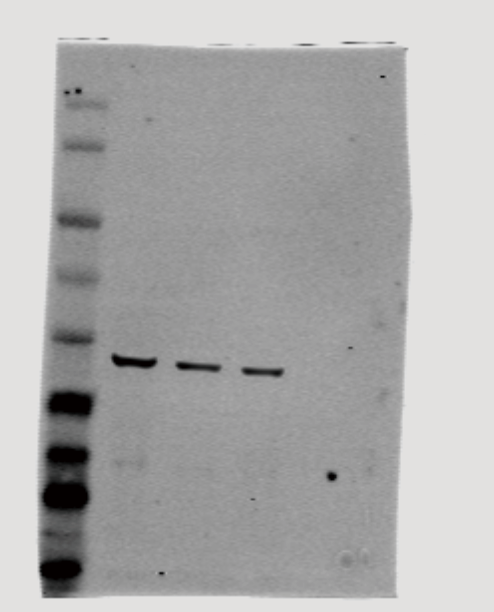

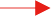

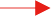

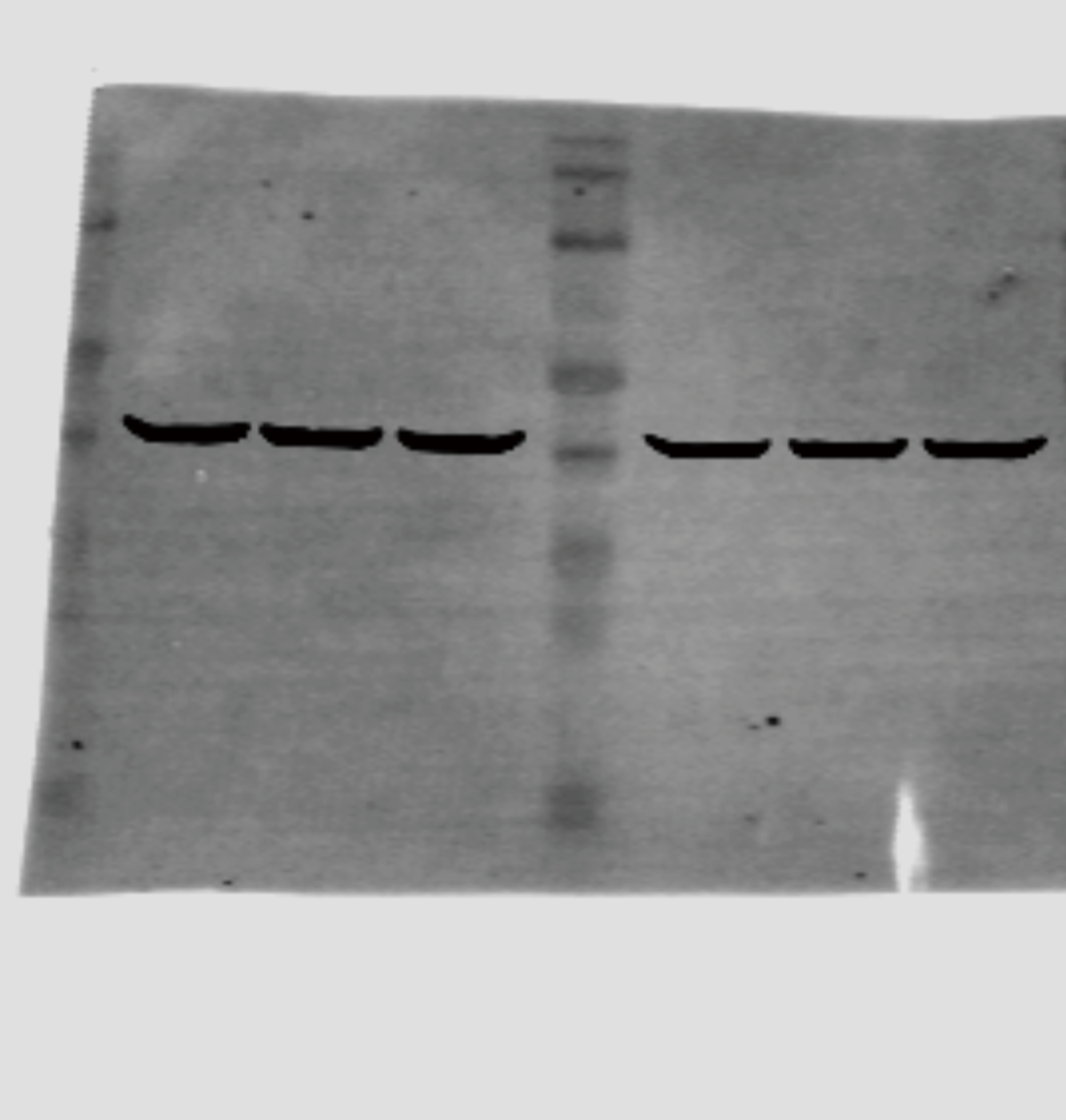

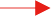

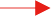


55 kDa

40 kDa

55 kDa

55 kDa

40 kDa

35 kDa 55 kDa

40 kDa

55 kDa

40 kDa

# Figure 2B A549-LMs

SKA3 43kDa

HK2 102kDa PKM2 58kDa GLUT3 54kDa LDHA 37kDa PDK1 47 kDa Actin 42 kDa


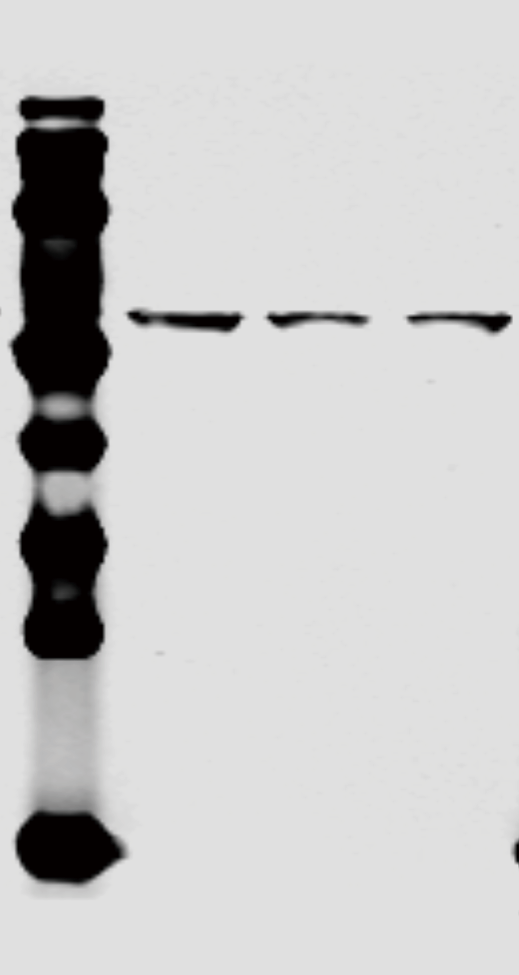

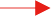

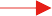


55 kDa

40 kDa

130 kDa

100 kDa


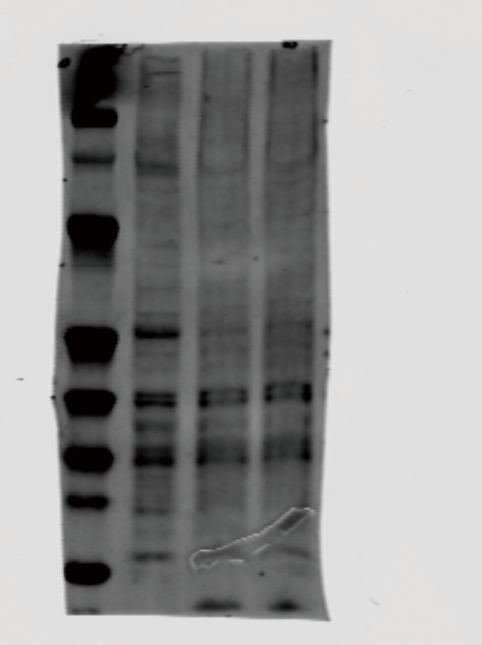

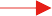

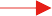

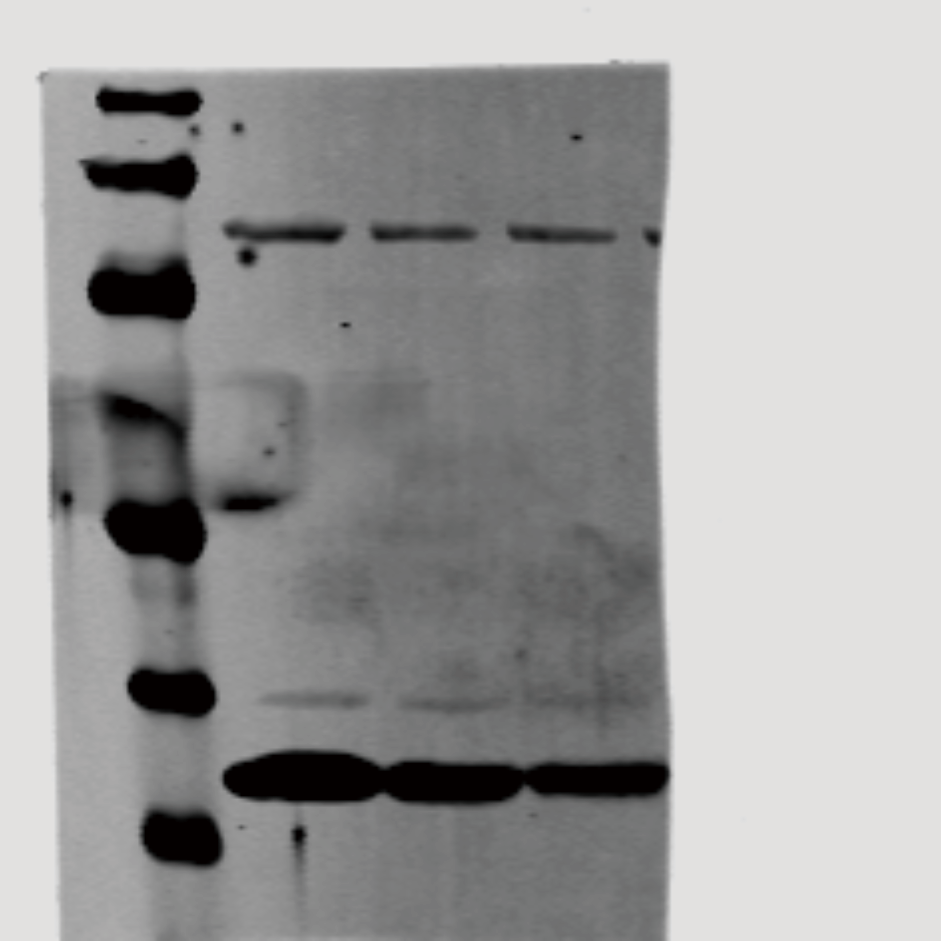

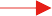

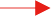


70kDa 55 kDa

70kDa 55 kDa


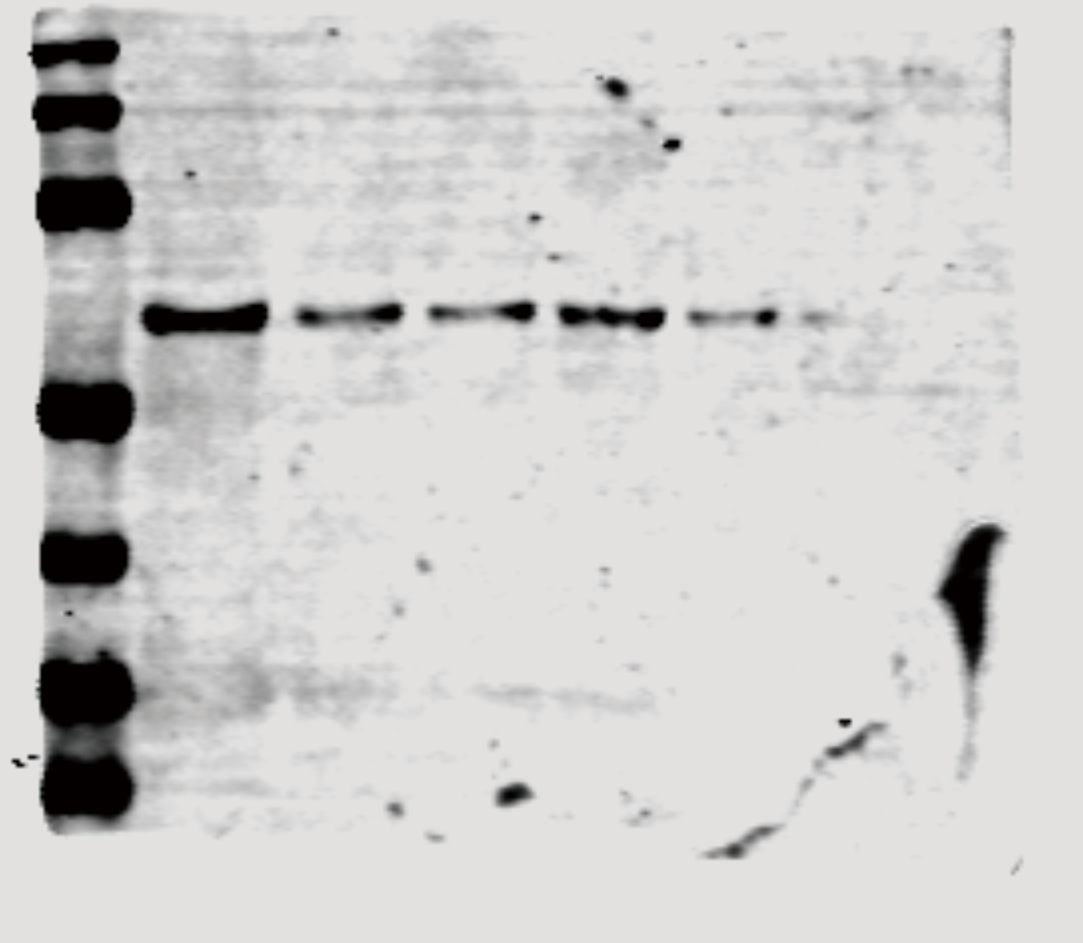

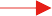

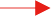


40 kDa


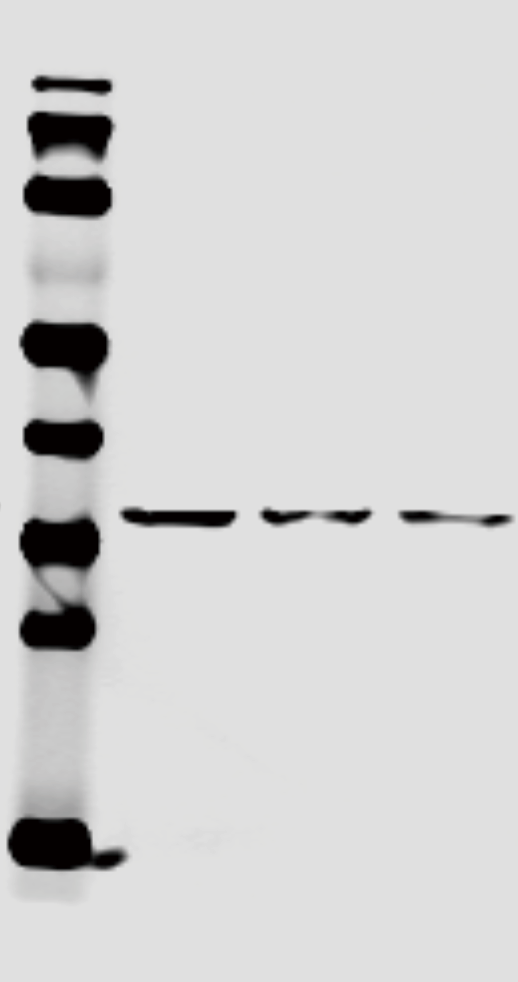

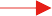

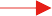


35 kDa

55 kDa

40 kDa


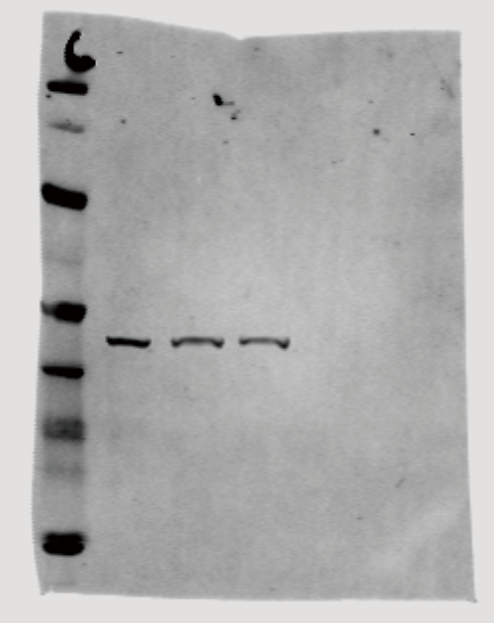

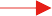

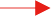


55 kDa

40 kDa


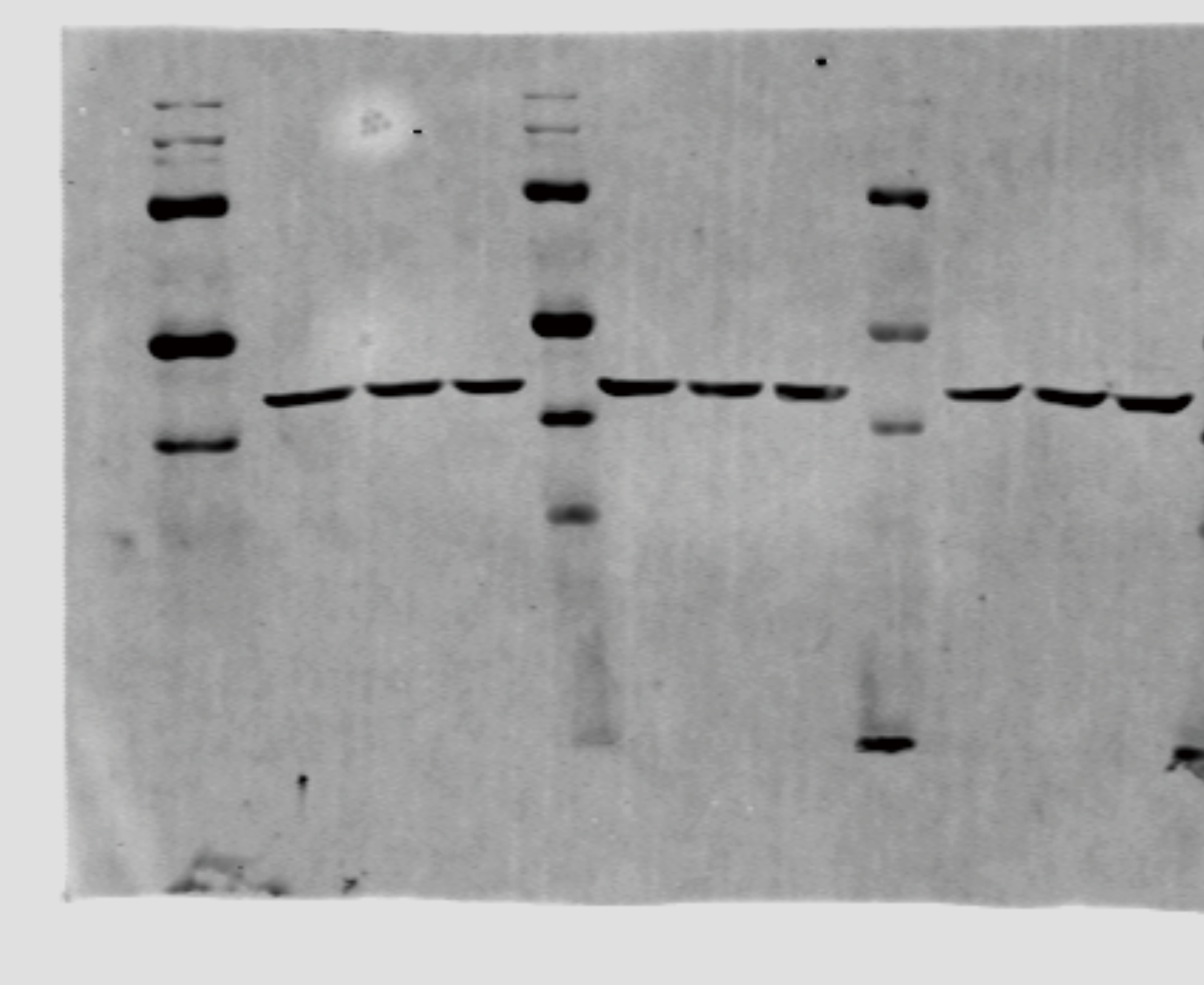

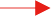

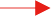


# Figure 3F-G A549

IP

IP

# Figure 3F-G A549-LMs

IP

IP

55 kDa


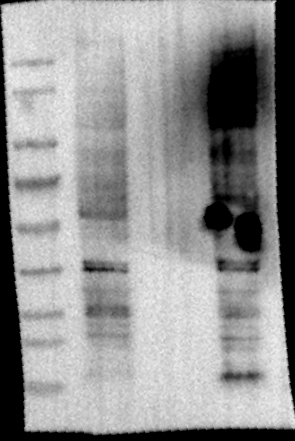

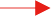

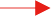

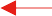


40 kDa

heavy chain IB:SKA3 43kDa

55 kDa

40 kDa IB:PHD2 46kDa


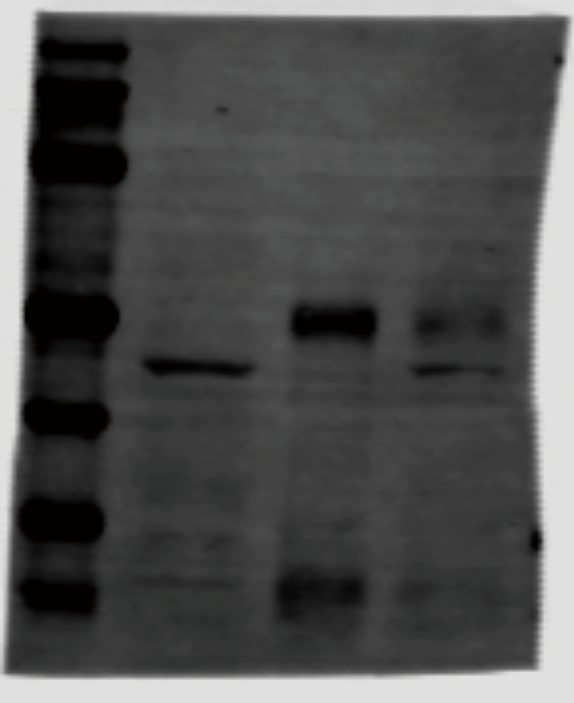

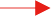

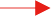


55 kDa

40 kDa IB:SKA3 43kDa


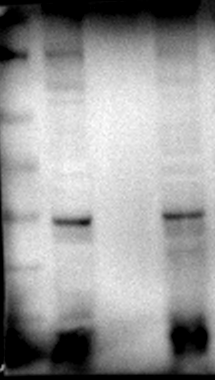

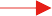

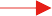


55 kDa

40 kDa IB:PHD2 46kDa


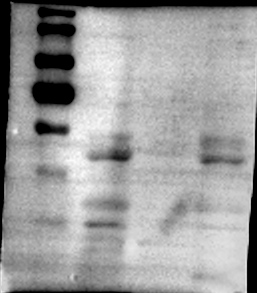

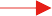

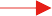


IP

IP

IP

IP

55 kDa


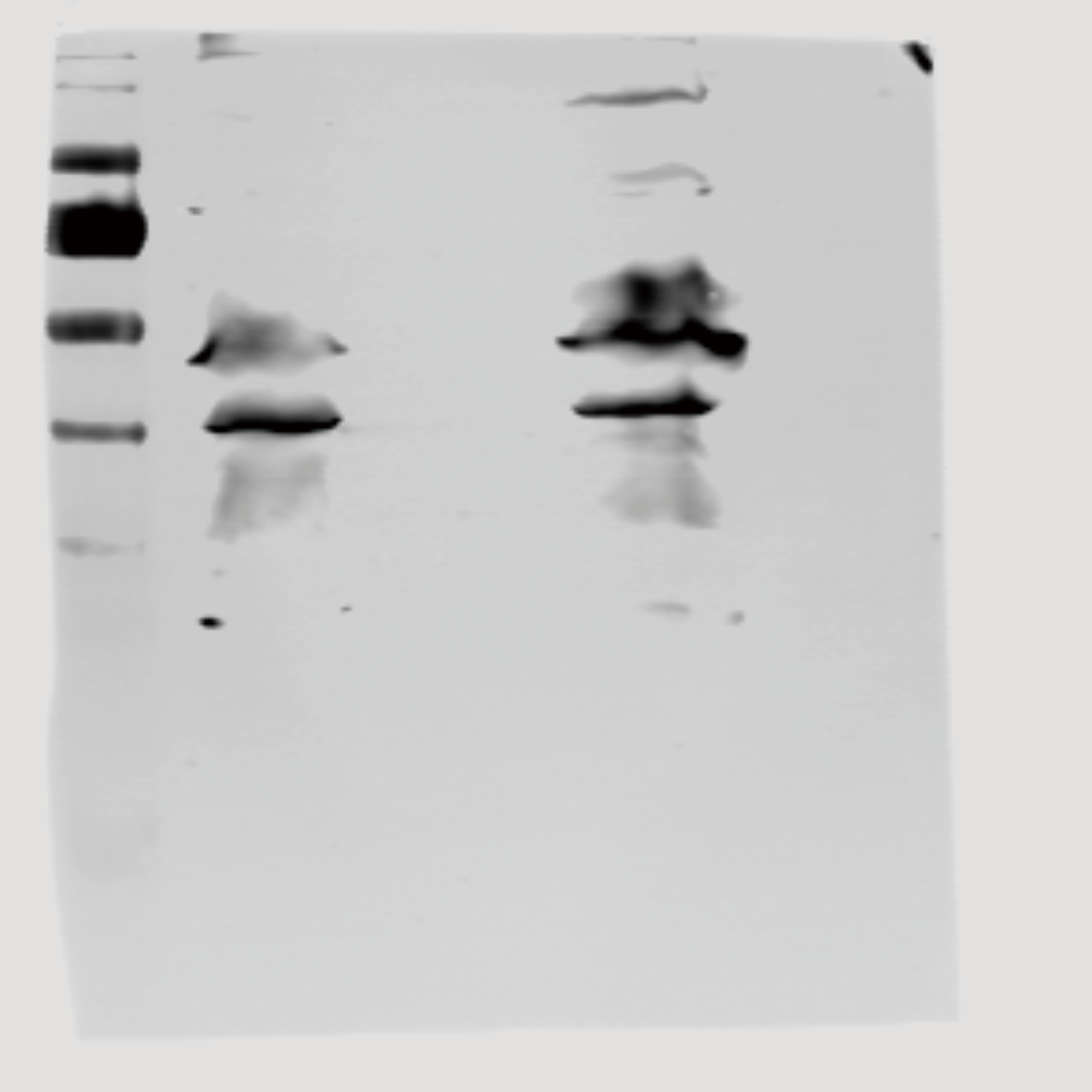

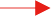

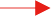


40 kDa

IB:PHD2 46kDa

55 kDa


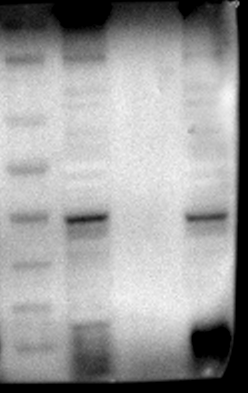

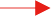

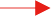

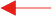


40 kDa IB:SKA3 43kDa

55 kDa

40 kDa


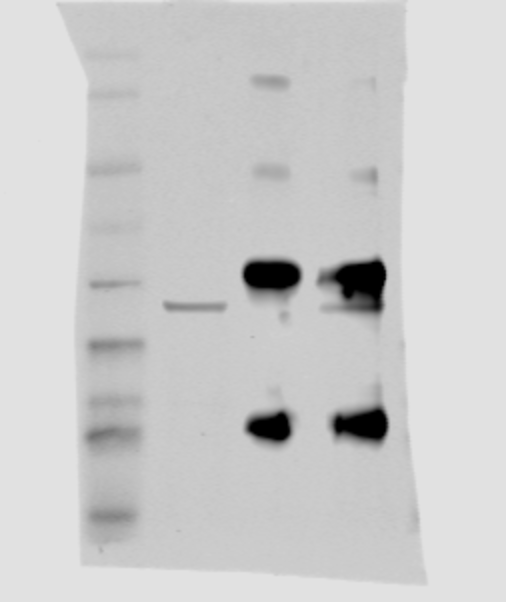

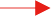

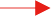


IB:PHD2 46kDa

55 kDa

40 kDa


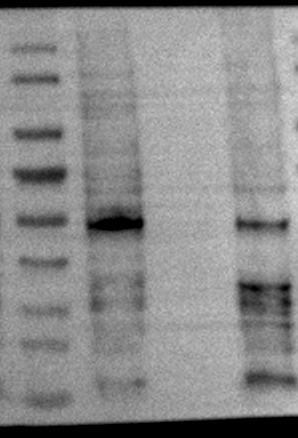

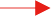

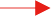


IB:SKA3 43kDa

light chain

# Figure 3I A549

PHD2 46kDa OH-HIF-1α 110 kDa

HIF-1α 120 kDa

VHL 24 kDa

Actin 42 kDa

55 kDa

40 kDa

130 kDa

100 kDa


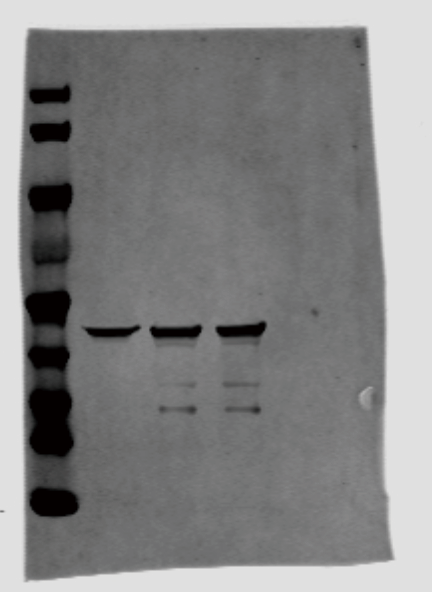

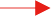

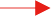


130 kDa

100 kDa


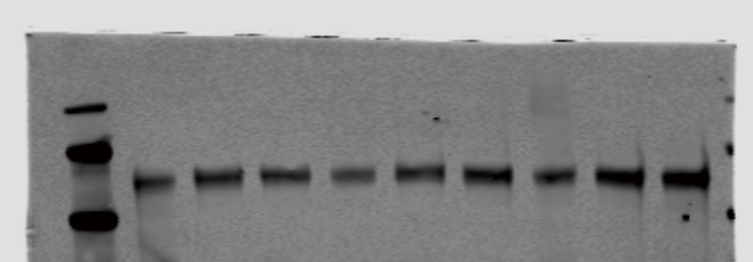

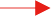

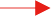

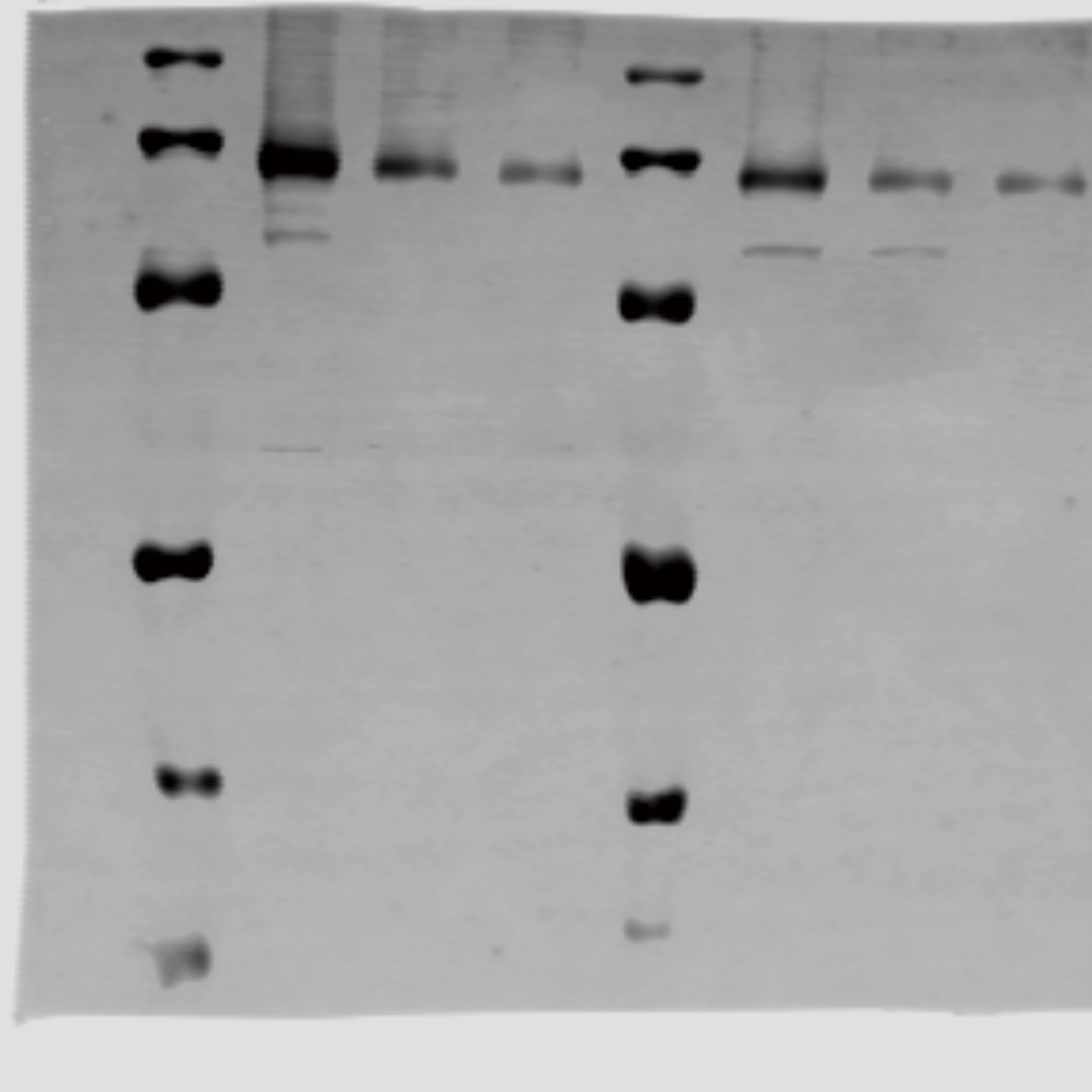

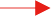

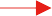


25 kDa

55 kDa


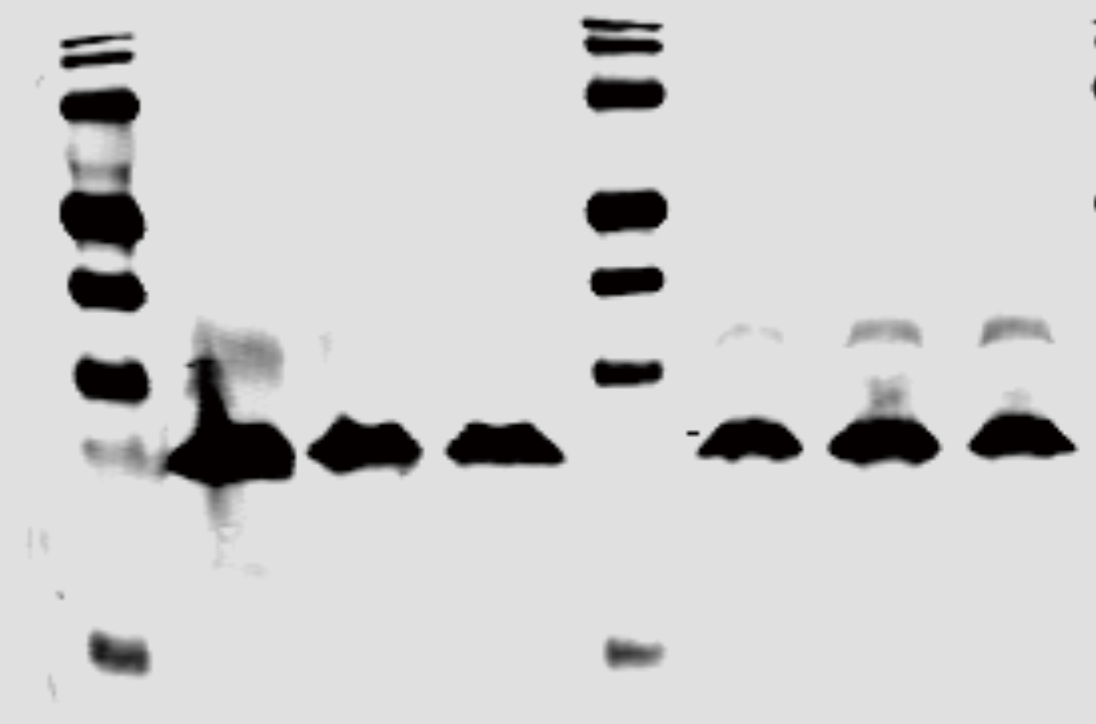

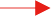

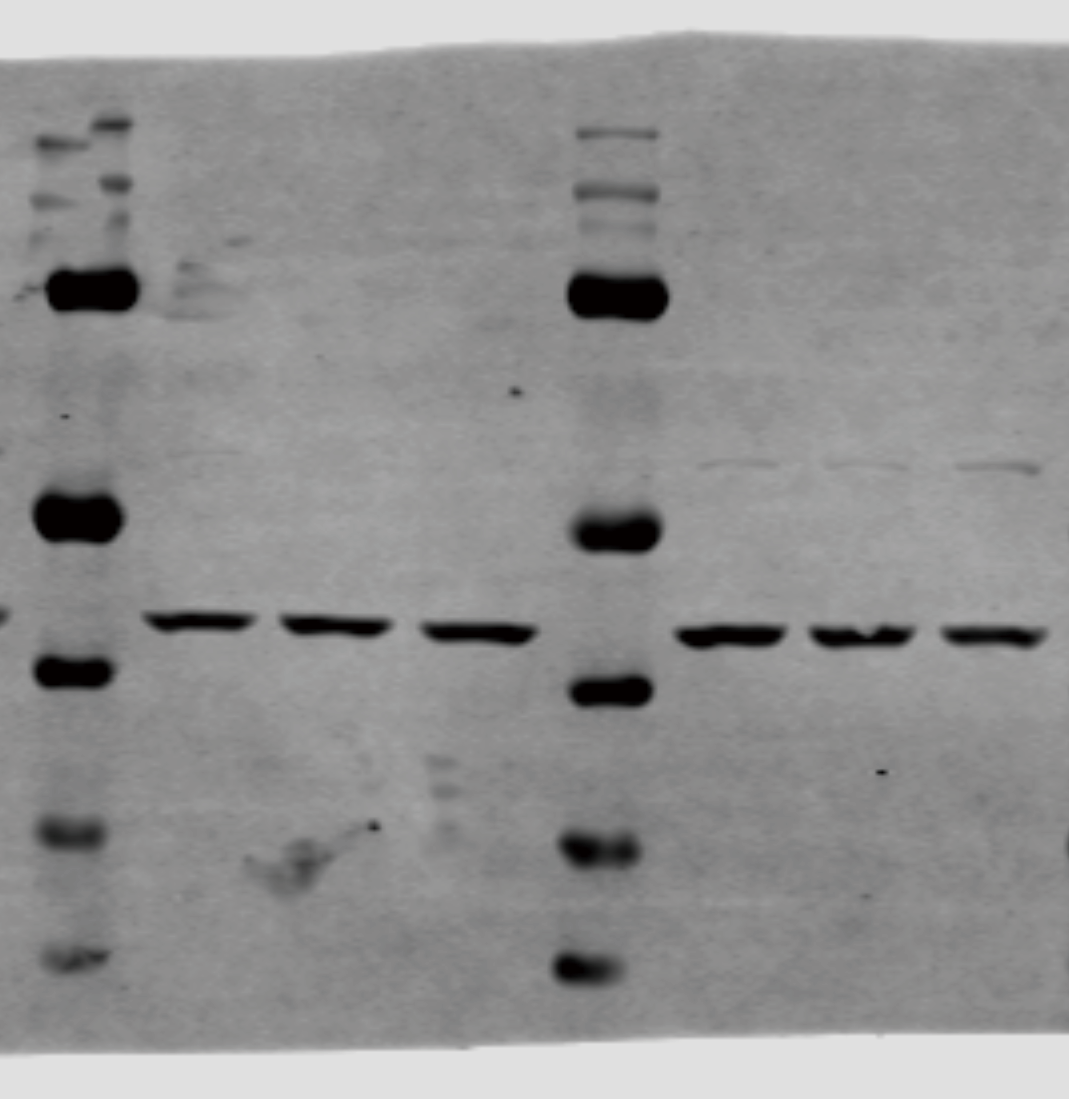

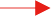

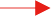


40 kDa

# Supplemental Figure 2D A549-LMs

PHD2 46kDa OH-HIF-1α 110 kDa HIF-1α 120 kDa VHL 24 kDa Actin 42 kDa


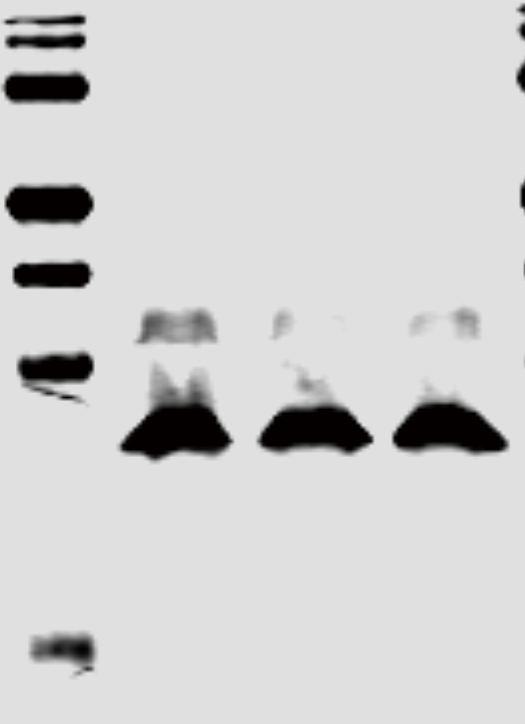

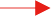


55 kDa

40 kDa

130 kDa

100 kDa


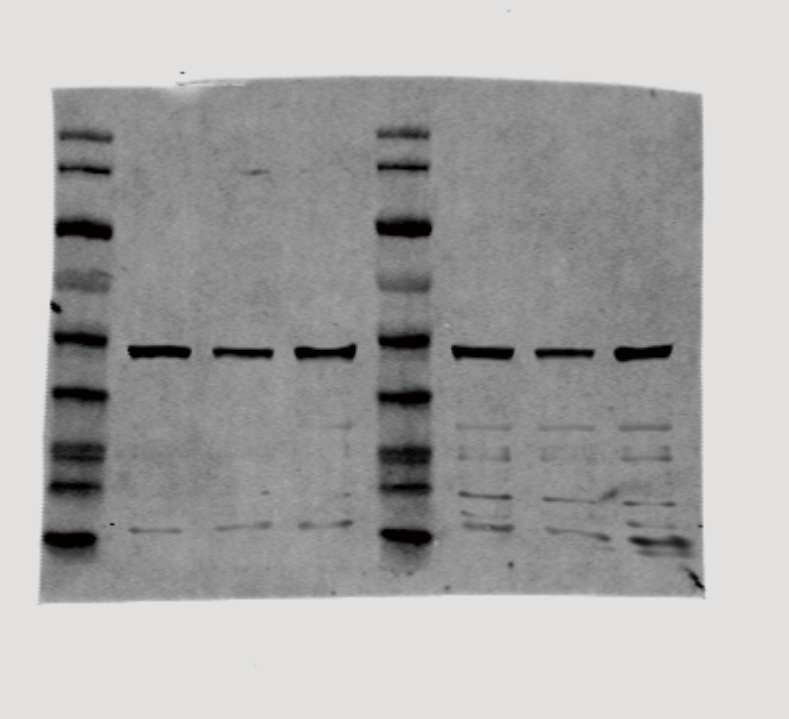

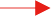

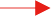

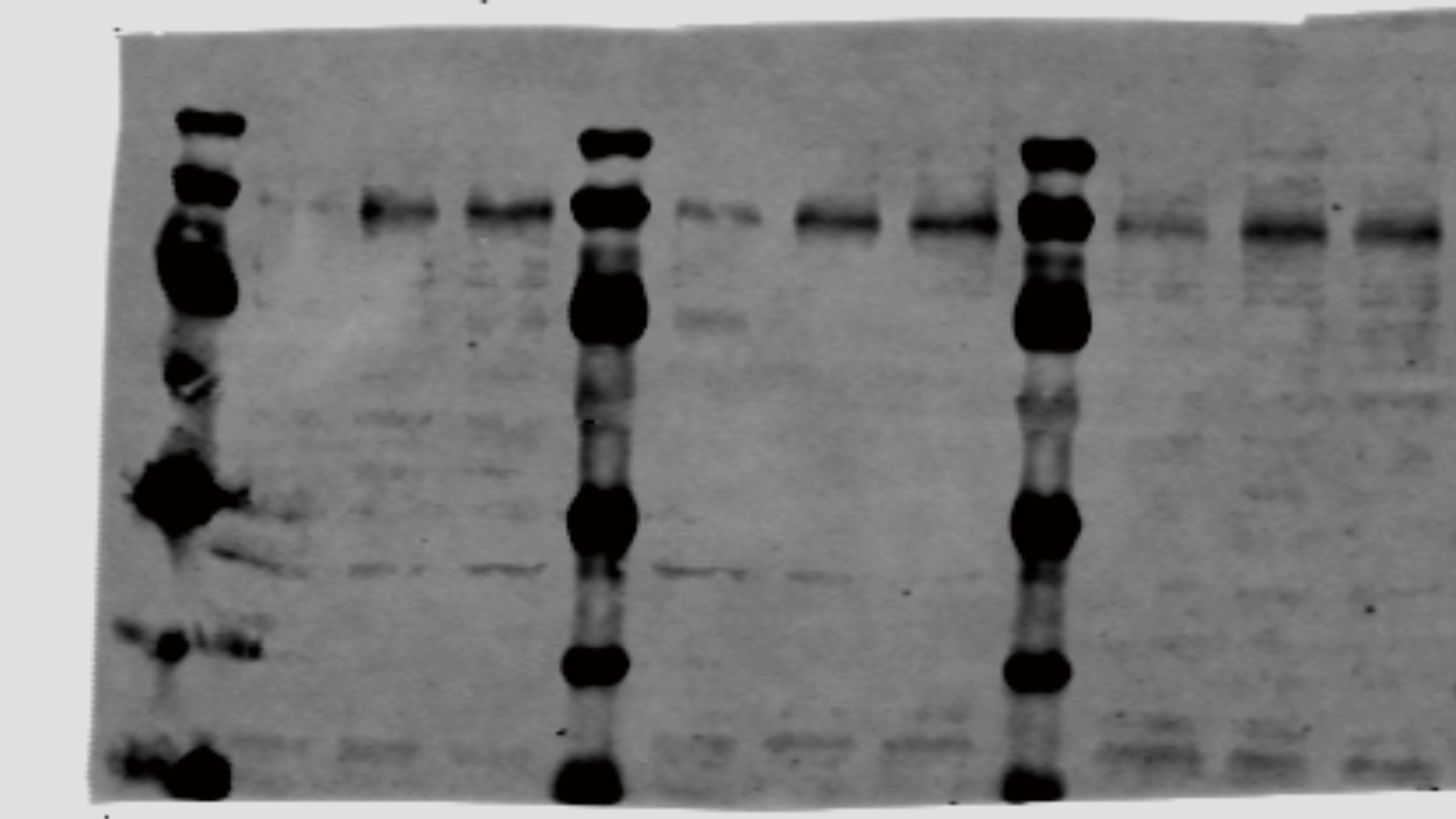

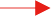

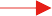


130 kDa

100 kDa


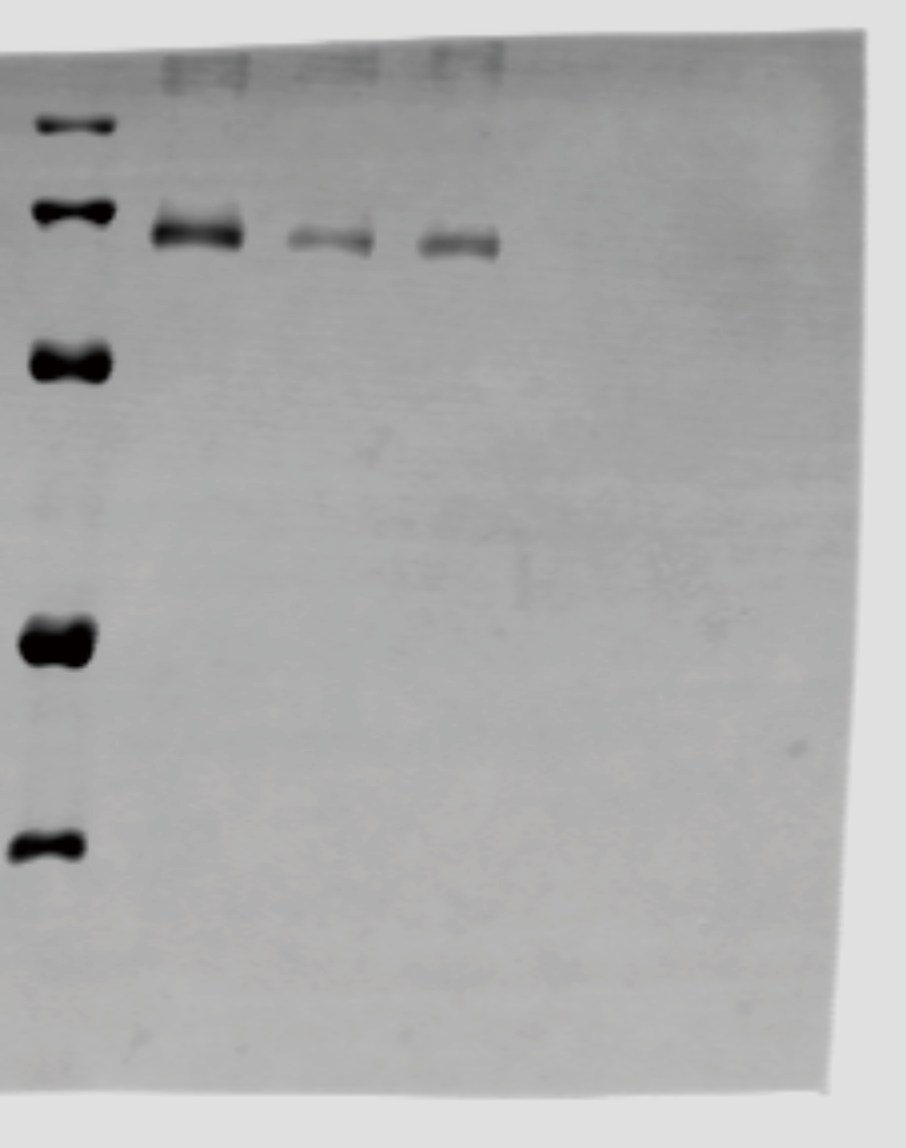

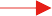

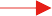


25 kDa

55 kDa


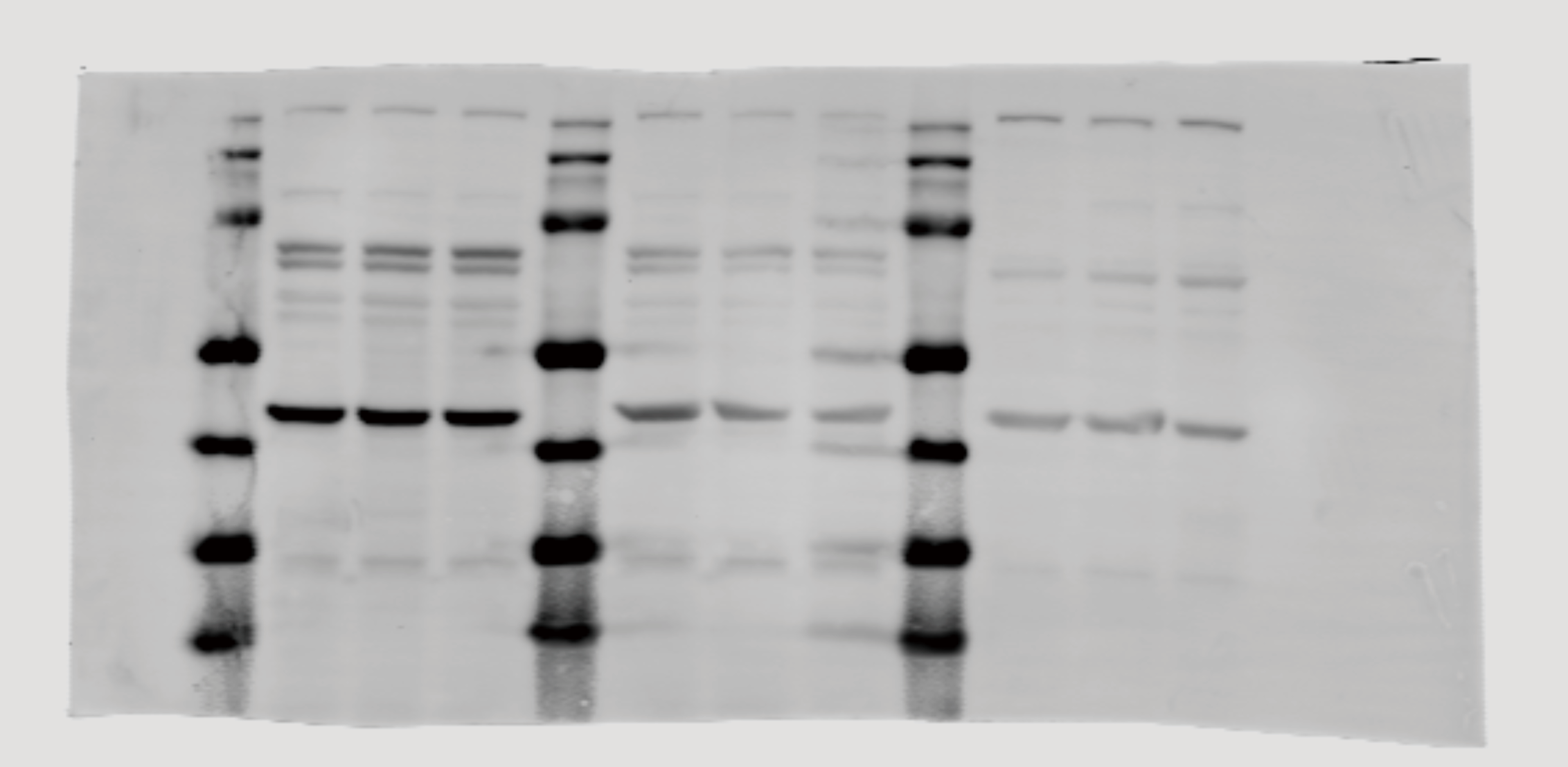

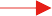


40 kDa

# Figure 3J A549

55 kDa

40 kDa

SKA3 43kDa

55 kDa

40 kDa

PHD2 46kDa OH-HIF-1α 110 kDa

130 kDa

100 kDa

130 kDa

100 kDa

HIF-1α 120 kDa Actin 42 kDa

55 kDa

40 kDa

# Supplemental Figure 2E A549-LMs

55 kDa

40 kDa

SKA3 43kDa PHD2 46kDa

55 kDa

40 kDa

130 kDa

100 kDa

OH-HIF-1α 110 kDa

130 kDa

100 kDa

HIF-1α 120 kDa Actin 42 kDa

55 kDa

40 kDa

# Figure 3K A549

Ctrl-NC + - + - Si-SKA3 - + -

| Ctrl-NC | + | - | + | - | Ctrl-NC | + | - | + | - |
| --- | --- | --- | --- | --- | --- | --- | --- | --- | --- |
| Si-SKA3 | - | + | - |  | Si-SKA3 | - | + | - |  |
| MG132 | - | - | + | + | MG132 | - | - | + | + |

MG132 - - + +

55 kDa

40 kDa SKA3 43kDa

130 kDa

100 kDa

HIF-1α 120 kDa

55 kDa

40 kDa Actin 42 kDa

# Supplemental Figure 2F A549-LMs

| Ctrl-NC | + | - | + | - | Ctrl-NC | + | - | + | - | Ctrl-NC | + | - | + | - |
| --- | --- | --- | --- | --- | --- | --- | --- | --- | --- | --- | --- | --- | --- | --- |
| Si-SKA3 | - | + | - |  | Si-SKA3 | - | + | - |  | Si-SKA3 | - | + | - |  |
| MG132 | - | - | + | + | MG132 | - | - | + | + | MG132 | - | - | + | + |

55 kDa

40 kDa SKA3 43kDa

130 kDa

100 kDa

HIF-1α 120 kDa

55 kDa

40 kDa Actin 42 kDa

# Figure 3L A549

Ctrl-NC Si-SKA3 Ctrl-NC Si-SKA3

CHX(min) 0 5 10

20 40 60

0 5 10

20 40 60

CHX(min) 0 5 10

20 40 60

0 5 10

20 40 60

130 kDa

100 kDa

HIF-1α 120 kDa

55 kDa

40 kDa

Actin 42 kDa

# Supplemental Figure 2G A549-LMs

|  | | | Ctrl-NC |  |  | Si-SKA3 |  |  |  |  | Ctrl-NC |  | Si-SKA3 |
| --- | --- | --- | --- | --- | --- | --- | --- | --- | --- | --- | --- | --- | --- |
| CHX(min) | 0 | 5 | 10 20 | 40 60 | 0 | 5 10 20 40 | 60 | CHX(min) | 0 | 5 | 10 20 40 60 | 0 | 5 10 20 40 60 |

130 kDa

100 kDa

HIF-1α 120 kDa

55 kDa

40 kDa Actin 42 kDa

# Figure 3M A549

IP: IgG

SKA3

55 kDa

40 kDa

IB:PHD2 46kDa

55 kDa

40 kDa

Input:PHD2 46kDa

40 kDa

Input:SKA3 43kDa

# Supplemental Figure 2H A549-LMs

IP: IgG

SKA3

55 kDa

40 kDa

55 kDa IB:PHD2 46kDa

40 kDa

55 kDa

40 kDa

Input:PHD2 46kDa

40 kDa

Input:SKA3 43kDa

# Figure 3N A549

IP: IgG

PHD2

IP: IgG

PHD2

130 kDa

IB:HIF-1α 120kDa

55 kDa

40 kDa IB:PHD2 46kDa

130 kDa

100 kDa

55 kDa

40 kDa

Input:HIF-1α 120kDa

Input:PHD2 46kDa

# Supplemental Figure 2I A549-LMs

IP: IgG

PHD2

IP: IgG

PHD2

130 kDa IB:HIF-1α 120kDa

55 kDa

40 kDa IB:PHD2 46kDa

130 kDa

100 kDa

55 kDa

40 kDa

Input:HIF-1α 120kDa

Input:PHD2 46kDa

# Figure 3P A549

IP: IgG

HIF-1α

IP: IgG

HIF-1α

IgG

HIF-1α

130 kDa

100 kDa

IB：HIF-1α 120 kDa

IB：ubiquitin

130 kDa

100 kDa

Input:HIF-1α 120 kDa

# Supplemental Figure 2K A549-LMs

IP: IgG

HIF-1α

IP: IgG

HIF-1α

IgG

HIF-1α

130 kDa

100 kDa

IB：HIF-1α 120 kDa

IB：ubiquitin

130 kDa

100 kDa

Input:HIF-1α 120 kDa

# Figure 3Q A549

IP: IgG

HIF-1α

IB：Ub-K48

Input:SKA3 43kDa

Input:HIF-1α 120 kDa

Input:β-Actin 43 kDa

# Supplemental Figure 2L A549-LMs

IP: IgG

HIF-1α

IB：Ub-K48

Input:SKA3 43kDa

Input:HIF-1α 120 kDa

Input:β-Actin 43 kDa

# Supplemental Figure 3A A549

HIF-1α 120 kDa β-Tublin 55 kDa Lamin B1 66kDa

130 kDa

100 kDa

55 kDa

40 kDa

100 kDa

55 kDa

# Supplemental Figure 3A A549-LMs

HIF-1α 120 kDa β-Tublin 55 kDa Lamin B1 66kDa

130 kDa

100 kDa

55 kDa

40 kDa

100 kDa

55 kDa

# Figure 4A A549

SKA3 43kDa HIF-1α 120 kDa

130 kDa

100 kDa

55 kDa

40 kDa

Actin 42 kDa

# Figure 4A A549-LMs

SKA3 43kDa

HIF-1α 120 kDa Actin 42 kDa

55 kDa

40 kDa

130 kDa

100 kDa

55 kDa

40 kDa

# Supplemental Figure 4A

SKA3 43kDa Actin 42 kDa

55 kDa

40 kDa

55 kDa

40 kDa

# Supplemental Figure 4F

HK2 102kDa GLUT3 54kDa

130 kDa

100 kDa

70 kDa

PDK1 47 kDa

PKM2 58kDa LDHA 37kDa Actin 42 kDa

70 kDa

55 kDa

40 kDa

40 kDa

35 kDa

# Figure 5B

Normoxia Hypoxia

+ - + -

- + - +

Normoxia Hypoxia

+ - + -

- + - +

Normoxia Hypoxia

+ - + -

- + - +

55 kDa

40 kDa

p53 53 kDa

HIF-1α 120 kDa

130 kDa

100 kDa

55 kDa

40 kDa

β-actin 42 kDa

# Figure 5E A549

p53 53kDa SKA3 43kDa

PHD2 46kDa

HIF-1α 120 kDa

VHL 24 kDa

OH-HIF-1α120 kDa

Actin 42 kDa

55 kDa

40 kDa

55 kDa

40 kDa

55 kDa

40 kDa

130 kDa

100 kDa

25 kDa

55 kDa

40 kDa

# Figure 5F A549-LMs

p53 53kDa SKA3 43kDa PHD2 46kDa HIF-1α 120 kDa

VHL 24 kDa

OH-HIF-1α120 kDa

Actin 42 kDa

55 kDa

40 kDa

55 kDa

55 kDa

40 kDa

40 kDa

130 kDa

100 kDa

25 kDa

55 kDa

40 kDa

# Supplemental Figure 5B

MDM2 90kDa

# Supplemental Figure 5C

MDM2 90kDa

100 kDa

Actin 42 kDa

100 kDa

Actin 42 kDa

40 kDa

40 kDa

# Supplemental Figure 5D A549 Supplemental Figure 5E A549-LMs

00 kDa

MDM2 90kDa

40 kDa

Actin 42 kDa

100 kDa

40 kDa

MDM2 90kDa

Actin 42 kDa

# Supplemental Figure 5F

IB: Ubiquitin

# Supplemental Figure 5G

100 kDa

MDM2 90kDa

MDM2 90kDa

Actin 42 kDa

40 kDa 55 kDa

p53 53 kDa

IB: Ubiquitin

100 kDa

40 kDa

Actin 42 kDa p53 53 kDa

55 kDa

40 kDa

# Supplemental Figure 5H

IB: Ubiquitin

# Supplemental Figure 5I

100 kDa

MDM2 90kDa

MDM2 90kDa

55 kDa

55 kDa

p53 53 kDa

p53 53 kDa

55 kDa

40 kDa

Actin 42 kDa

55 kDa

Actin 42 kDa

IB: Ubiquitin

100 kDa

40 kDa

# Figure 6A A549

p53 53kDa

SKA3 43kDa

HIF-1α 120 kDa

Actin 42 kDa

55 kDa

40 kDa

55 kDa

40 kDa

130 kDa

100 kDa

55 kDa

40 kDa

# Figure 6A A549-LMs

55 kDa

p53 53kDa SKA3 43kDa HIF-1α 120 kDa Actin 42 kDa

130 kDa

100 kDa

40 kDa 55 kDa

40 kDa

55 kDa

40 kDa

# Supplemental Figure 6C A549

HK2

PDK1

PKM2

GLUT3

Actin LDHA

HK2

GLUT3

Actin LDHA

PDK1

PKM2

# Figure 7B A549

HIF-1α 120 kDa

130 kDa

100 kDa

55 kDa

40 kDa

SKA3 43kDa

55 kDa

40 kDa

Actin 42 kDa

# Figure 7B A549-LMs

HIF-1α 120 kDa

SKA3 43kDa

Actin 42 kDa

130 kDa

100 kDa

55 kDa

40 kDa

55 kDa

40 kDa

55 kDa

# Figure 7H HT-A549

SKA3 43kDa

130 kDa

100 kDa

HIF-1α 120 kDa

Actin 42 kDa

55 kDa

55 kDa

# Figure 7H HT-PC9

SKA3 43kDa

130 kDa

100 kDa

HIF-1α 120 kDa

55 kDa

Actin 42 kDa

# Supplemental Figure 7C A549

HIF-1α 120 kDa

100 kDa

40 kDa

SKA3 43kDa

40 kDa

Actin 42 kDa

# Supplemental Figure 7C A549-LMs

100 kDa

HIF-1α 120 kDa

SKA3 43kDa

Actin 42 kDa

40 kDa

40 kDa

# Supplemental Figure 7D

SKA3 43kDa

HIF-1α 120 kDa

p53 53kDa

Actin 42 kDa

100 kDa

55 kDa

40 kDa

40 kDa

40 kDa
